# ﻿Insights to the taxonomy and phylogeny of the genus *Ptilagrostis* worldwide (Poaceae, Stipeae) with a key to species identification, checklist and outlines for further studies

**DOI:** 10.3897/phytokeys.249.128729

**Published:** 2024-11-15

**Authors:** Marta Krzempek, Ewelina Klichowska, Marcin Nobis

**Affiliations:** 1 Institute of Botany, Faculty of Biology, Jagiellonian University, Gronostajowa 3, Kraków 30–387, Poland Jagiellonian University Kraków Poland

**Keywords:** Distribution, false needlegrass, micromorphology, morphology, phylogeny, *Ptilagrostis*, taxonomy

## Abstract

*Ptilagrostis* (false needlegrass) is a genus of high-mountain grasses distributed in Central and North-East Asia, as well as in North America. The phylogenetic position of the genus *Ptilagrostis* within the Stipeae is well defined based on micromorphological patterns of lemma epidermis and moleculs. However, there is a lack of a comprehensive taxonomic revision of the genus in its entire distribution range. In this study, we performed comprehensive analyses using integrative taxonomic methods, aiming at both micromorphological and macromorphological analyses, and molecular analyses based on SNPs obtained from DArT genome-wide sequencing, in which we considered all taxa representing the genus in order to establish phylogenetic relationships between its members. We analysed all species possessing the characteristic ‘*Ptilagrostis* pattern’ of the lemma epidermis, with a particular reference to species possessing the terminal segment of the awn (seta) covered by short (up to 1 mm long) hairs that, until now, were treated as representatives of the genus *Achnatherum*. Following with the result of our molecular, morphological and anatomical analyses, the genus *Ptilagrostis* is represented by 15 species, one subspecies and five varieties organised in three well supported phylogenetic clades corresponding to the three sections: *Ptilagrostis*, *Barkworthia* and *Chenella*. In this paper, we provide an original key to identifying false needlegrass species, together with a checklist containing the intrageneric species-organisation. In addition, for each species, we present the data regarding nomenclatural types, morphological description, and information on the geographical distribution, habitat preferences and altitudinal ranges. We proposed two new varieties within the genus *Ptilagrostis*, P.glabrifoliavar.himalayensis and P.concinnavar.xizangensis, and the new section Chenella comprising three species with awns scabrous or covered by very short hairs up to 0.3 mm long. Additionally, we transfer *Stipachingii* to *Ptilagrostischingii*, Achnatherumchingiivar.laxum to P.chingiivar.laxum, and Ptilagrostisconcinnasubsp.schischkinii to P.junatoviivar.schischkinii. Lectotypification was made for three taxa, Stipamongholicavar.minutiflora, *P.czekanowskii*, and *P.tibetica*.

## ﻿Introduction

Grasses (Poaceae) are one of the most prevalent flowering plants, thriving on every continent worldwide (Gibson 2009; Hodkinson 2018). With an impressive presence, they rank as the fifth most abundant family among angiosperms, boasting a rich diversity of approximately 11,000 species spread across over 700 genera (Gibson 2009; Bhatt and Thaker 2021). Within the family, the Stipeae emerges as a noteworthy and widely distributed group. Species representing the tribe are found in grassland ecosystems across nearly all continents, excluding Antarctica (Tzvelev 1977; Romaschenko et al. 2012; Nobis et al. 2020; Barkworth 2007; Cialdella et al. 2010, 2013; Everett et al. 2009). Recognised as a monophyletic and well-defined taxon, Stipeae contributes significantly to the global diversity of grasses (Soreng et al. 2022). There are approximately 680 species within Stipeae (Barkworth 2007), organised into 21 to 28 genera (Romaschenko et al. 2008; Peterson et al. 2019; Soreng et al. 2022).

One of the genera representing Stipeae and comprised of high mountain species occurring on fresh and wet meadows, swards, alpine steppes, rocky grasslands, and screes is *Ptilagrostis* Griseb. (false needlegrass) (Tzvelev 1976; Wu and Phillips 2006; Johnston 2006; Zhang et al. 2016a, 2016b, 2017; Tzvelev and Probatova 2019; Nobis et al. 2019a). In the middle of the 19^th^ century, Grisebach (1852) described the genus *Ptilagrostis*, into which he transferred *Stipamongholica* Turcz. ex Trin. The representatives of the genus *Ptilagrostis* are characterised by having erect stems, spikelets with solitary florets, short, plumose and geniculately-bent awns, and lemmas discontinuously covered by long hairs, usually scabrous and hairless in the middle part (Tzvelev 1976; Freitag 1985; Barkworth 1983; Romaschenko et al. 2012; Tzvelev and Probatova 2019; Nobis et al. 2019a). The current species-organisation within the genus *Ptilagrostis* is based on a combination of macromorphology, micromorphological patterns of the lemma epidermis, and molecular analyses. To date, species representing the genus Ptilagrostis are divided into two sections, namely sect. Ptilagrostis and sect. Barkworthia M. Nobis, A. Nobis & A. Nowak (Nobis et al. 2015).

Micromorphological patterns of the lemma epidermis (LEP) are regarded as conservative and important for understanding evolutionary relationships within Stipeae (Tzvelev 1977; Barkworth and Everett 1987; Romaschenko et al. 2012; Nobis and Nobis 2013; Nobis et al. 2019a, 2020). Within this tribe, two types of LEP are found. The first one, called the maize-like epidermal pattern, is characterised by numerous, square to rounded silica bodies and short fundamental cells, and occurs in representatives of achnatheroid grasses (Romaschenko et al. 2012, 2014; Nobis et al. 2019a, 2020). The second, called saw-like epidermal pattern, is characterised by elongated fundamental cells and reniform, ovate, oblong to elongated silica bodies sometimes associated with cork cells. This type of pattern occurs in stipoid grasses, such as *Stipa* L., *Orthoraphium* Nees, *Neotrinia* (Tzvelev) M. Nobis, P.D. Gudkova & A. Nowak, *Trikeraia* Bor, *Piptatherum* P. Beauv. and *Ptilagrostis*, although there are slight differences among particular genera (Romaschenko et al. 2012, 2014; Nobis et al. 2019a, 2019b, 2020). Based on the LEP analysis, several species have recently been transferred from the genera *Stipa*, *Piptatherum* and *Ptilagrostis* to *Achnatherum* P. Beauv. and from the genus *Achnatherum* to *Neotrinia* or *Ptilagrostis* (Nobis and Nobis 2013; Banfi et al. 2018; Nobis et al. 2019a, 2019b, 2020). However, patterns of the lemma micromorphology are still understudied in many species, and further research is required to identify their generic affiliation. To date, within the genus *Ptilagrostis*, lemma micromorphology has been analysed in eight species: *P.alpina* (F. Schmidt) Sipliv., *P.concinna* (Hook. f.) Roshev., *P.contracta* Z.S. Zhang & W.L. Chen, *P.duthiei* (Hook.f.) M.Nobis & P.D.Gudkova, *P.malyschevii* Tzvelev, *P.mongholica* (Turcz. ex Trin.) Griseb., *P.porteri* (Rydb.) W.A. Weber and *P.yadongensis* Keng & Tang (Barkworth 1983; Romaschenko et al. 2012, 2014; Nobis and Nobis 2013; Nobis et al. 2019a, 2019b, 2020; Zhang et al. 2017). Species belonging to the genus *Ptilagrostis* are characterised by having elongated fundamental cells with sinuate to lobate sidewalls, frequent silica bodies with constricted side walls and cork cells (Romaschenko et al. 2012, 2014; Nobis and Nobis 2013; Nobis et al. 2019a). The pattern of lemma micromorphology confirmed both the affiliation of *Ptilagrostispelliotii* (Danguy) Grubov to *Achnatherum*, as well as *Achnatherumduthiei* (Hook. f.) P.C. Kuo & S.L. Lu and *Stipabhutanica* Noltie to *Ptilagrostis* (Nobis et al. 2019a, 2020).

Characters of leaf anatomy play an important role in taxonomy within the Poaceae. Examples include: the outline of the leaf cross-section, number of ribs, presence of bulliform cells, arrangement of sclerenchyma in relation to the vascular bundles and number of vascular bundles (Renvoize 1985; López and Devesa 1991; Conert 1998; Namaganda et al. 2009; Martínez-Sagarra et al. 2017). Their application aims to improve the taxonomy of morphologically very similar species. Leaf anatomy was utilised mainly to examine the genus *Festuca* L. and other taxonomically challenging genera within the Poaceae, such as *Stipa*, *Anthoxanthum* L. or *Sesleria* Scop. (e.g., Pimentel and Sahuquillo 2003; Kuzmanović et al. 2009; Namaganda et al. 2009; Martínez-Sagarra et al. 2017; Gudkova et al. 2023). Analysis of the morphological structures of leaves has also been applied in some species of the genus *Ptilagrostis*, including *P.concinna*, *P.junatovii* Grubov, *P.malyschevii*, *P.mongholica* and *P.porteri* (Malyschev 1965; Tzvelev 1974; Barkworth 1983).

Recently, molecular studies have shed light on the phylogenetic relationships of the Stipeae and have also led to further changes in the taxonomy of the genus *Ptilagrostis*. The latest studies, based on the ITS and cpDNA sequences, were conducted by Romaschenko et al. (2008, 2012, 2014), Hamasha et al. (2012), and Zhang et al. (2017). They analysed 13 species representing the genus *Ptilagrostis*, however, in different combinations and with different sets of species in particular studies. Based on the results of molecular analyses, Hamasha et al. (2012) transferred *Ptilagrostispelliotii* (Danguy) Grubov to *Achnatherum*, whereas Peterson et al. (2019) transferred *P.kingii* to *Ptilagrostiella*. However, in most cases, analyses failed to resolve intrageneric relationships between taxa since some of the species were organised in polytomies (Hamasha et al. 2012; Zhang et al. 2017) or the findings obtained from plastid and nuclear sequences were not consistent (Romaschenko et al. 2014). Thus, further research that takes into account traditional macro- and micromorphological studies combined with modern wide-genome analyses is needed.

Previous studies on the genus *Ptilagrostis* were often limited to specific geographic regions, selected species or subsets of species. In this study, we aim to fill this gap by summarising all of the *Ptilagrostis* species worldwide. Because there is a lack of current comprehensive treatment of taxa representing this genus, the main goals of this study are to provide: i) morphological and molecular analysis (latest based on SNPs derived from genome-wide DArT sequencing) of the species representing *Ptilagrostis* with the particular reference to the species having upper segments of the awn scabrous or covered with up to 1 mm long hairs; ii) micromorphological analysis of the lemma epidermal structures of the members of this genus; iii) analysis of vegetative leaves’ cross-sections; iv) an identification key for all members of the genus; v) taxonomic and nomenclatural summary that also includes morphological description, notes on habitat preferences and distribution of particular false needlegrasses; (vi) intrageneric species organisation.

## ﻿Methods

### ﻿Plant material

The research was conducted utilising plant material preserved in the following herbaria: Academy of Science, Uzbekistan Central Herbarium (**TASH**), Botanische Staatssammlung München Herbarium (**M**), Herbarium of the Institute of Botany, Jagiellonian University (**KRA**), Institute of Botany, Chinese Academy of Sciences Chinese National Herbarium in Beijing (**PE**), Kunming Institute of Botany, Chinese Academy of Sciences Herbarium (**KUN**), Ludwig-Maximilians-Universität München Herbarium (**MSB**), Missouri Botanical Garden Herbarium (**MO**), Herbarium of the Institute of Applied Ecology, Academia Sinica in Shenyang, China (**IFP**), P.N. Krylov Herbarium of Tomsk State University (**TK**), Herbarium of the Institute of Botany, Kyrgyz Academy of Sciences in Bishkek (**FRU**), Herbarium of the Institute of Botany, Kazakh Academy of Sciences in Almaty (**AA**), Herbarium of the Moscow State University (**MW**), Central National Herbarium known also as CNH or Calcutta herbarium in India (**CAL**), Museum of Evolution in Uppsala (**UPS**), New York Botanical Garden Herbarium (**NY**), Royal Botanic Garden Edinburgh Herbarium (**E**), Royal Botanic Gardens Herbarium in Kew (**K**), The Herbarium at the Natural History Museum, London (**BM**), University of Colorado Museum Herbarium (**COLO**), Utah State University Intermountain Herbarium (**UTC**), V.L. Komarov Botanical Institute Herbarium in St. Petersburg (**LE**) (acronyms of the herbaria are used according to Index Herbariorum, Thiers 2024). We reviewed over 400 herbarium specimens representing all species of *Ptilagrostis* during these studies.

### ﻿Morphological measurements and multivariate analyses

The herbarium material was examined using biometric analysis. Measurements were conducted using a stereomicroscope (Nikon SMZ800) with a graduated scale eyepiece and ruler. In total, we used 342 selected, well-developed and undamaged specimens for morphological measurements. In detail, we examined 281 specimens of Ptilagrostissect.Ptilagrostis, species and the number of specimens for each are as follows: *P.alpina* (8), *P.arcuata* Z.S. Zhang & W.L. Chen (4), *P.concinna* (25), P.concinnavar.xizangensis M. Nobis & Krzempek (2), P.dichotomaKeng ex Tzvelevvar.dichotoma (38), P.dichotomavar.roshevitsiana Tzvelev (5), P.glabrifoliaX.Y. Zhang & W.L. Chenvar.glabrifolia (5), P.glabrifoliavar.himalayensis M. Nobis & Krzempek (2), P.junatoviivar.junatovii (30), P.junatoviivar.schischkinii (Tzvelev) M. Nobis & Krzempek (1), *P.luquensis* P.M. Peterson, Soreng & Z.L. Wu (4), *P.malyschevii* (84), P.mongholicasubsp.mongholica (63), P.mongholicasubsp.porteri (Rydb.) Barkworth (6) and *P.tibetica* (Mez) Tzvelev (8). Moreover, we included 62 specimens of false needlegrasses having awn with hairs on seta up to 1 mm long, including 20 specimens representing sect. Barkworthia with *P.bhutanica* (Noltie) M. Nobis (11) and *P.yadongensis* (9), and 42 specimens representing sect. Chenella, described below, with specimens of *P.chingii* (Hitchc.) M. Nobis & Krzempek (27), *P.contracta* Z.S. Zhang & W.L. Chen (6) and *P.duthiei* (9). Moreover, we included six specimens of *Ptilagrostiellakingii* (Bol.) Romasch. as an outgroup. List of examined specimens is presented in Suppl. material 1. Each specimen was analysed on the basis of 28 quantitative characters (length of the lemma and palea, length of lemma lobes, callus length, callus base length and width, length of hairs on the dorsal part of the callus, length of hairs on the ventral part of the callus, awn length, length of lower segment of the awn (column), length of terminal segment of the awn (seta), width of the awn base, length of hairs on the lower segment of the awn (in the middle part of the segment), length of hairs on the terminal segment of the awn (near geniculation), length of culms, length of vegetative leaves, width of the leaves, number of vascular bundles on the leaf cross-section, length of ligule on the lower culm sheath, length of ligule on the middle culm sheath, length of ligule on the upper culm sheath, length of the longest ligules on the external leaf-sheaths on the vegetative shoot, length of the longest ligules on the internal leaf-sheaths on the vegetative shoot, length of bracts below the panicle, length of panicle, length of the lower pedicles within the panicle, length of the lower glume, length of the upper glume, number of spikelets in lower pedicle within the panicle) and nine qualitative traits (character of the lower, middle and upper culm sheaths (glabrous/pubescent), character of leaf-sheaths on the vegetative shoots (glabrous/pubescent), width of panicle (narrow/wide), character of pedicles (glabrous/pubescent), character of the abaxial and adaxial surface of leaves (glabrous, scabrous, pilose), presence of hairs on the top of the anthers). In addition, three ratios were measured: the length of the lemma to the length of the palea, the length of hairs on the lower segment of the awn to the length of hairs on the terminal segment of the awn and the length of the lower glume to the length of upper glume. In accordance with the principles of numerical taxonomy, every individual specimen was treated as an operational taxonomic unit (OTU) (Sokal and Sneath 1963). Of 40 characters studied, the 10 most informative traits (i.e., those having the strongest factor loadings (>0.60) that allowed the best distinction of the *Ptilagrostis* species) were selected in the Principal Component Analysis (PCA) of the entire dataset and 18 key morphological characters, that led to distinguish examined taxa, were used in the UPGMA analysis (Table 1). Moreover, to visualise the differences in the species group that have a terminal segment of the awn (seta) covered with up to 1 mm long hairs and belonging to sections *Chenella* and *Barkworthia*, 11 characters allowed for the best distinction for analysed species, and at the same time having the strongest factor loadings (>0.60), were chosen for the final PCA. Due to generally high morphological similarity among particular *Ptilagrostis* representatives, we decided to also use qualitative characters in addition to the quantitative characters. Thus, for the Principal Coordinates Analysis (PCoA) we selected 12 of the most important characters (11 quantitative and one qualitative), enabling better differentiation of the studied species. The characteristics selected for analysis are presented in Table 1. The findings derived from all biometric studies are summarised in the key to species identification and in morphological descriptions of the taxa. In order to reveal significant differences among the means of characters across investigated species having up to 1 mm long hairs on the seta, a Kruskal-Wallis test was performed for all characters included in PCA. Subsequently, post-hoc evaluations were conducted using a multiple comparison test. The analyses were performed in Statistica 13 (TIBCO Software, USA) and PAST v. 4.03 (Hammer et al. 2001).

**Table 1.** Morphological characters used in the numerical analyses provided for all the taxa representing *Ptilagrostis*. Analyses marked with the asterix (*) were done for the *Ptilagrostis* species having seta covered with hairs up to 1 mm long.

Abbreviation	Character	PCA	UPGMA	PCA*	PCoA*
PL	Length of palea (mm)	+	+	+	+
L/P	Ratio: length of lemma to the length of palea	+	+	+	+
LL	Length of the lobes (mm)		+	+	+
AL	Length of the awn (mm)	+	+	+	+
UL	Length of hairs on the terminal segment of the awn (seta) (mm)	+	+	+	+
HR	Length of hairs on the lower segment of the awn (column) to length of hairs on the terminal segment of the awn (seta) ratio (mm)	+	+		
CL	Length of culm (cm)	+	+	+	+
LW	Width of the leaf (mm)		+	+	+
CN	Number of vascular bundles		+	+	+
IL	Length of the longest ligules on the internal leaf-sheaths on the vegetative shoot	+	+		
PeL	Length of the lower pedicle within the panicle (cm)	+	+	+	+
LP	Length of panicle	+			
GL	Length of the lower glume (mm)		+	+	+
GU	Length of the upper glume (mm)	+			
GL/U	Ratio: length of lower glume to length of upper glume		+	+	+
PW	Width of the panicle		+		+
HA	Presence of hairs on the top of the anthers		+		
HL	Presence of hairs on the lower segment of the awn		+		
HM	Presence of hair on the middle part of the lemma		+		
HP	Presence of hairs on the pedicles		+		
PB	Presence of bracts below the panicle		+		
No. of characters examined		10	19	11	12

### ﻿Micromorphology analysis

The upper surface of the lemma epidermis was subjected to micromorphological observations. Samples were obtained from the middle part of the panicle and were examined from the base to the top. The presence, location and shape of prickles and hooks, the distribution and length of macro-hairs, the length and shape of long cells and the shape of silica bodies were examined. Using a JCF-1100E ion sprayer (JEOL, Japan), the dried material was coated with gold and then photographed under various magnifications on a Hitachi S-4700 scanning electron microscope. Measurements were taken using ImageJ software (LOCI, University of Wisconsin, USA). Terminology was adopted from Nobis et al. (2019a, 2020). Studied samples are indicated in Suppl. material 1.

### ﻿Vegetative leaf cross-section analysis

Cross-sections through the middle part of the leaf blade of the studied species were made using a razor blade, followed by microscopic observations under a Nikon Eclipse 80i compound microscope. Leaf blade length and width, number of vascular bundles and ribs, location and appearance of sclerenchyma, and presence of hairs were analysed. Specimens of the following species are represented in the analysis: *P.alpina* (8), *P.arcuata* (3), *P.bhutanica* (9), *P.chingii* (27), *P.concinna* (15), *P.contracta* (2), *P.dichotoma* (35), *P.duthiei* (5), *P.malyschevii* (81), *P.mongholica* (57) and *P.yadongensis* (8) were reviewed. Studied samples are indicated in Suppl. material 1.

### ﻿Genomic library preparation and DArT sequencing

Molecular analyses were based on 31 samples belonging to *P.alpina*, *P.arcuata*, *P.bhutanica*, *P.chingii*, *P.concinna*, *P.contracta*, P.dichotomavar.dichotoma, P.dichotomavar.roshevitsiana, *P.duthiei*, *P.glabrifolia*, *P.junatovii*, *P.luquensis*, *P.malyschevii*, *P.mongholica* and *P.yadongensis* (see Suppl. material 1). Two additional samples of *Ptilagrostiellakingii* were selected for outgroup comparison. Whole genomic DNA was isolated using a Genomic Mini AX Plant Kit (A&A Biotechnology, Poland). NanoDrop One (Thermo Scientific, USA) was used to perform the quantification check. Following the DArTseq methodology, each sample was diluted up to a concentration of 50–100 ng/μL. Purified DNA (1–2 μg for each sample) was shipped to Diversity Arrays Technology Pty ltd (Canberra, Australia) for sequencing and marker identification.

DArTseq is a hybrid of DArT complexity reduction techniques with next-generation sequencing technologies, tuned for each organism and application to pick the most appropriate complexity reduction strategy (Sansaloni et al. 2011; Kilian et al. 2012; Cruz et al. 2013). Based on the results of testing several enzyme combinations for complexity reduction for *Ptilagrostis*, Diversity Arrays Technology Pty Ltd. chose the *Pst*I-Mse I enzyme combination. This section was performed according to the procedures previously published (Baiakhmetov et al. 2020; Nobis et al. 2023; Sinaga et al. 2024). All DNA samples were processed in digestion/ligation reactions as described by Kilian et al. (2012), but replacing a single *Pst*I-compatible adaptor with two different adaptors corresponding to two different restriction enzyme overhangs. The *Pst*I-compatible adapter was designed to include Illumina flowcell attachment sequence, sequencing primer sequence, and “staggered”, varying length barcode region, similar to the sequence reported previously (Elshire et al. 2011). The reverse adapter contained a flowcell attachment region and *Mse*I-compatible overhang sequence. Only “mixed fragments” (*Pst*I-*Mse*I) were effectively amplified by PCR using an initial denaturation step of 94 °C for 1 min, followed by 30 cycles with the following temperature profile: denaturation at 94 °C for 20 s, annealing at 58 °C for 30 s, and extension at 72 °C for 45 s, with an additional final extension at 72 °C for 7 min. After PCR, equimolar amounts of amplification products from each sample of the 96-well microtiter plate were bulked and applied to c-Bot (Illumina, USA) bridge PCR, followed by sequencing on Hiseq2500 (Illumina, USA). The sequencing (single read) was run for 77 cycles. Sequences generated from each lane were analysed utilising proprietary DArT analytical pipeline methods. The poor-quality sequences were filtered away from fastq files, with more stringent selection criteria for the barcode region than for the rest of the sequence. Thanks to this, the assignments between the sequences and specific samples during the “barcode split” were reliable. During the marker calling step, ca. 2.5 mln sequences per barcode/sample were identified. As a result, short read sequences were obtained, which, after removing the restriction site-associated adapter, were 20–69 bp long, depending on the quality.

### ﻿SNP data analysis

For the downstream analyses, we applied co-dominant single nucleotide polymorphisms (SNP) markers, which were analysed using the RStudio package “dartR” (Gruber et al. 2018) and “devtools” (Wickham et al. 2022). Data filtering included the following steps: 1) removing all monomorphic loci, 2) removing loci identified (=called) in less than 95% of all individuals, 3) removing loci with reproducibility below a predetermined threshold (<1), 4) filtering sequence tags contained more than one SNP, to keep randomly selected one of them, and 5) filtrating loci based on the criteria of a minor allele frequency (MAF) (threshold 1%). Maximum Likelihood (ML) tree was generated based on 12,502 SNP loci (processed using R) for 33 samples of seventeen taxa (*P.alpina*, *P.arcuata*, *P.bhutanica*, *P.chingii*, *P.concinna*, *P.contracta*, P.dichotomavardichotoma, P.dichotomavar.roshevitsiana, *P.duthiei*, *P.glabrifolia*, *P.junatovii*, *P.luquensis*, *P.malyschevii*, *P.mongholica*, *P.yadongensis* and *Ptilagrostiellakingii* as outgroup). The genlight object was converted to the FASTA file (package dartR), and heterozygous locations were replaced with standard ambiguity codes. The FASTA file was then analysed using MEGA version 11.0.13 (Tamura et al. 2021), with the GTR (General Time Reversible) model chosen as the best-fitting substitution model based on AIC values and the bootstrap method as the phylogenetic test with 1,000 replications.

## ﻿Results

### ﻿Numerical analysis

**Figure 1.**
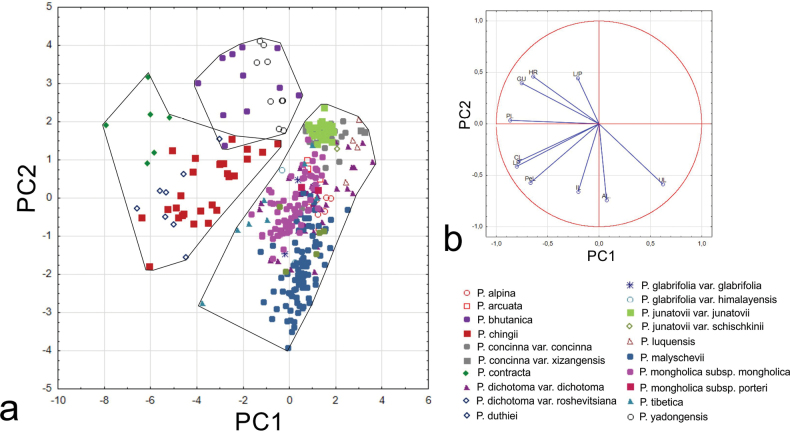
Scatter plot of (**a**) the Principal Component Analysis (PCA) performed on 10 quantitative characters (**b**) with projection of the variables on the factor plane PC1 × PC2 for all *Ptilagrostis* species and all specimens examined. List of specimens examined is presented in Suppl. material 1.

**Figure 2.**
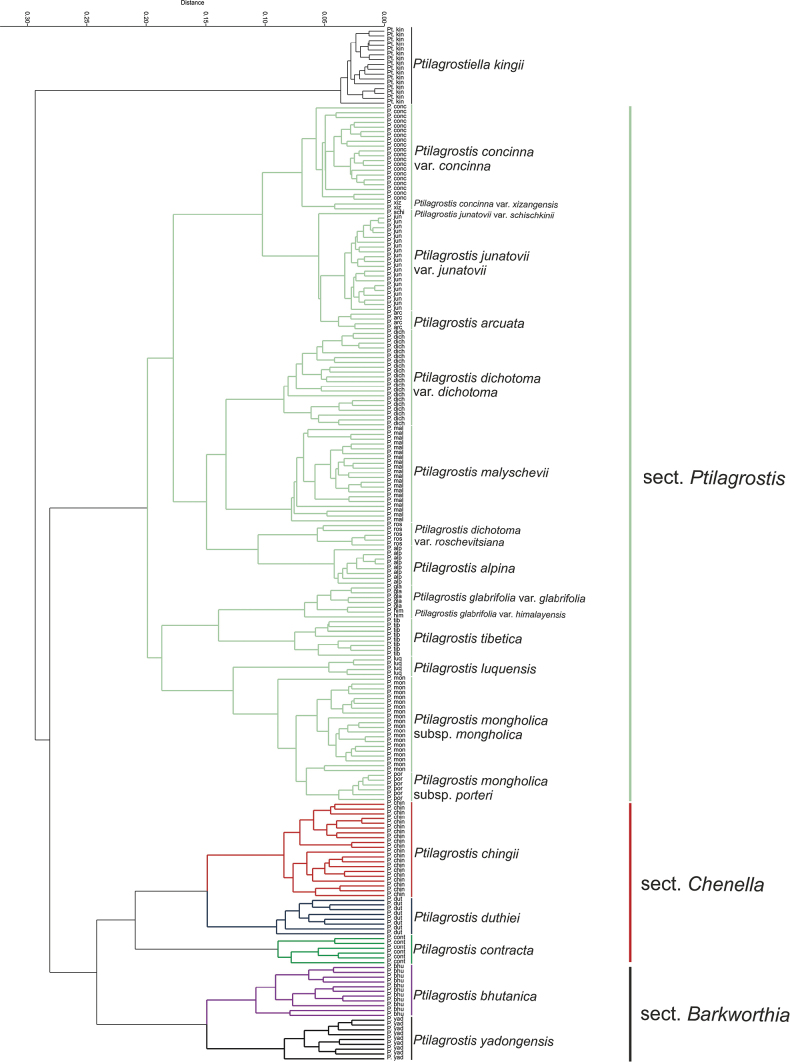
Cluster analysis (UPGMA) of selected specimens of all *Ptilagrostis* species. List of specimens examined is presented in Suppl. material 1.

Principal Component Analysis (PCA) based on 10 most informative quantitative characters (PL, L/P, AL, UL, HR, CL, IL, PeL, LP, GU) for all species representing *Ptilagrostis*, revealed that the first three principal components accounted for 77.14% of all character variation (Table 2) (PC1–39.11%, PC2–25.32%, PC3–12.71%). A high correlation (≥ 0.7) with the first axis is displayed by four characters: PL, CL, LP and GU, while the one, AL, is highly correlated with the second axis (Table 2). The most significant values of H statistics were observed for AL and UL. The scattered diagram of principal components defined by the first two axes PC1 vs. PC2 constitutes the best two-dimensional representation of the data and shows within the cloud of points the three groups (Fig. 1a). The first group located in the right part of the plot, comprises the taxa representing sect. Ptilagrostis, whereas in the left part of the diagram there are two discreet groups comprising OTUs of the five species *P.bhutanicaP.chingii*, *P.contracta*, *P.duthiei* and *P.yadongensis* that represent sections *Barkworthia* and *Chenella*. These species are characterised by the upper segment of the awn (seta) covered with short (up to 1 mm long) hairs. A similar organisation of *Ptilagrostis* representatives divided into three main clades (Fig. 2) is revealed by the cluster analysis (UPGMA) based on 19 quantitative and qualitative morphological characters (Table 1). For better readability we presented in the diagram up to 20 specimens per examined taxon (Fig. 2; complete result is presented in Suppl. material 2: fig. S1). *Ptilagrostiellakingii* differed from all analysed false needlegrasses by having the first segment of the awn covered by short, ca. 0.2 mm long, and hard prickles as opposed to hairs 0.4–3.0 mm long and soft prickles. *Ptilagrostis* species are divided into three clades. The first clade includes OTUs of *P.bhutanica* and *P.yadongensis* that represent sect. Barkworthia, while the second comprises the OTUs of *P.chingii*, *P.contracta* and *P.duthiei* that are members of the sect. Chenella. These two clades are sister to the third clade that is most numerous in species and includes all the remaining Ptilagrostis species and comprises sect. Ptilagrostis. These OTUs are organised into two subsequent subclades. The first includes *P.alpina*, *P.arcuata*, P.concinnavar.concinna, P.concinnavar.xizangensis, P.dichotomavar.dichotoma, P.dichotomavar.roshevitsiana, P.junatoviivar.junatovii, P.junatoviivar.schischkinii and *P.malyschevii*. The second subclade includes P.glabrifoliavar.glabrifolia, P.glabrifoliavar.himalayensis, *P.luquensis*, P.mongholicasubsp.mongholica, P.mongholicasubsp.porteri and *P.tibetica*.

**Table 2.** Results of the Principal Component Analysis (PCA) of all studied species of *Ptilagrostis* for ten morphological characters (the highest factor loadings are bolded); Kruskal-Wallis test with H and p values. For character abbreviations, see Table 1.

Character	PC1	PC2	PC3	H value	p value
PL	-**0.86**	0.04	0.01	224.80	<0.05
L/P	-0.21	0.45	-**0.72**	229.92	<0.05
AL	0.07	-**0.74**	-0.45	**270.88**	<0.05
UL	0.62	-0.59	0.02	**250.31**	<0.05
HR	-0.61	0.47	-0.26	131.44	<0.05
CL	-**0.79**	-0.36	0.32	197.82	<0.05
IL	-0.21	-**0.66**	-0.58	228.23	<0.05
LP	-**0.80**	-0.41	0.24	216.45	<0.05
PeL	-**0.67**	-0.56	0.02	191.36	<0.05
GU	-**0.75**	0.40	-0.07	218.86	<0.05
PL	-**0.86**	0.04	0.01	224.80	<0.05

**Figure 3.**
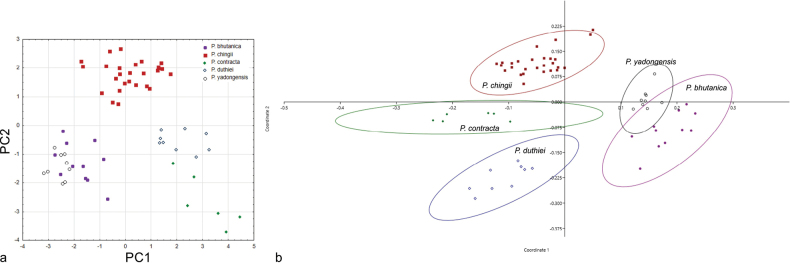
Scatter plot of (**a**) the Principal Component Analysis (PCA) performed on eleven quantitative characters with projection of the variables on the factor plane PC1 × PC2 for *Ptilagrostis* species with seta covered with short hairs up to 1 mm long (**b**) the Principal Coordinates Analysis (PCoA) performed on one qualitative and eleven quantitative characters with projection of the variables on the factor plane PC1 × PC2.

Separate PCA and PCoA analyses were carried out to clarify the variation among *Ptilagrostis* species with terminal awn segment (seta) covered with hairs up to 1 mm long (Fig. 3). The PCA analysis, based on 11 quantitative characters (PL, L/P, AL, LL, UL, CL, LW, CN, PeL, GL, GL/U), with the highest scores of the first three principal components provides the greatest contribution to explaining differences within and between groups. Variation within examined species is best explained by four of 11 traits (≥ 0.7, Table 3). The first three components explain 76.83% of all trait variation (PC1–34.77%, PC2–27.60%, PC3–14.46%). A high correlation with the first axis is displayed by two characters: PL and CL, while the two characters, AL and GL, are highly correlated with the second axis (Table 3). The PCA scatter plot of the first two axes showed distinctly separate clusters comprising OTUs in four groups (Fig. 3a). The PCoA analysis performed using one qualitative and 11 quantitative characters shows a separation of five groups of OTUs corresponding to each of the examined species (Fig. 3b). Significant differences were detected across all quantitative characters studied also according to the Kruskal-Wallis test (Table 3). The most significant values of H statistics were observed for L/P, LL, CL, LW and CN. The results of multiple comparison post-hoc tests are presented in Table 4. Various characters were identified as significant depending on the taxon. All the examined characters proved effective in distinguishing at least one pair of taxa. The most distinguishable species combinations were *P.chingii* – *P.yadongensis* and *P.duthiei* – *P.yadongensis* differed in nine and eight characters, respectively. Five characters were able to distinguish among the species combinations of *P.bhutanica* – *P.chingii*, *P.bhutanica – P.contracta* and *P.contracta* – *P.chingii*, four characters to distinguish among *P.bhutanica* – *P.duthiei*, *P.bhutanica* – *P.yadongensis*, *P.chingii – P.contracta* and *P.chingii – P.duthiei*. None of the characters examined effectively distinguished the *P.contracta* – *P.duthiei* pair of species (Table 4).

**Table 3.** Results of the Principal Component Analysis (PCA) of the studied Ptilagrostis species representing sect. Barkworthia and sect. Chenella, for 12 morphological characters (the highest factor loadings are bolded); Kruskal-Wallis test with H and p values. For character abbreviations, see Table 1.

Character	PC1	PC2	PC3	H value	p value
PL	**0.89**	0.05	0.03	36.42	<0.05
L/P	-0.65	-0.50	-0.36	**41.11**	<0.05
LL	0.30	0.61	0.62	**50.76**	<0.05
AL	0.20	-**0.72**	0.30	25.20	<0.05
UL	0.47	-0.50	0.37	26.25	<0.05
CL	**0.87**	0.34	0.00	**43.01**	<0.05
LW	0.52	-0.65	-0.35	**44.56**	<0.05
CN	0.68	-0.50	-0.40	**49.72**	<0.05
PeL	0.68	0.36	-0.08	27.52	<0.05
GL	0.31	-**0.74**	0.37	34.66	<0.05
GL/U	-0.44	-0.43	0.65	26.56	<0.05

**Table 4.** The results of multiple comparison post-hoc tests: + – significant, p < 0.05, ns – not significant (abbreviations: *Ptilagrostisbhutanica* – bhu, *P.chingii* – chin, *P.contracta* – con, *P.duthiei* – dut, *P.yadongensis* – yad). For character abbreviations, see Table 1.

Character	post-hoc test									
	bhu-chin	bhu -con	bhu -dut	bhu -yad	chin-con	chin-dut	chin-yad	con-dut	con-yad	dut-yad
PL	+	+	+	ns	ns	ns	+	ns	+	+
L/P	+	+	+	ns	ns	ns	+	ns	ns	+
LL	+	+	ns	ns	ns	+	+	ns	ns	ns
AL	ns	ns	ns	ns	+	ns	+	ns	ns	ns
UL	ns	ns	ns	+	ns	ns	+	ns	ns	+
CL	+	+	+	ns	ns	ns	+	ns	+	+
LW	+	ns	ns	ns	+	+	ns	ns	+	+
CN	ns	ns	ns	+	+	+	ns	ns	+	+
PeL	ns	ns	+	ns	ns	ns	+	ns	ns	+
GL	ns	+	ns	+	+	+	+	ns	ns	ns
GL/U	ns	ns	ns	+	ns	ns	+	ns	+	+
No. of significant differences	5	5	4	4	4	4	9	0	5	8

### ﻿Micromorphology of the *lemma* epidermis

**Figure 4.**
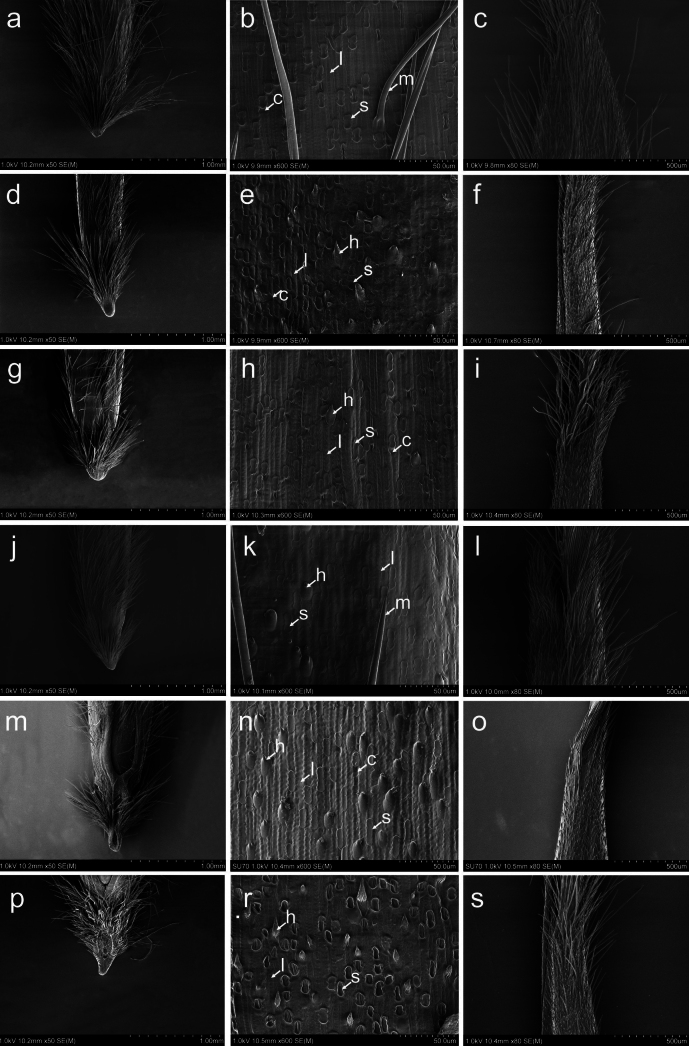
SEM morphology of the floret of *Ptilagrostistibetica* (**a–c**), *P.bhutanica* (**d–f**), *P.chingii* (**g–i**), *P.contracta* (**j–l**), *P.duthiei* (**m–o**) and *P.yadongensis* (**p–s**). Callus and the lower part of the lemma (**a, d, g, j, m, p**), epidermal pattern of the middle part of lemma (**b, e, h, k, n, r**), top of lemma (**c, f, i, l, o, s**). Abbreviations: c = cork cell, h = hook, l = long cell, s = silica body, m = macro-hair.

**Figure 5.**
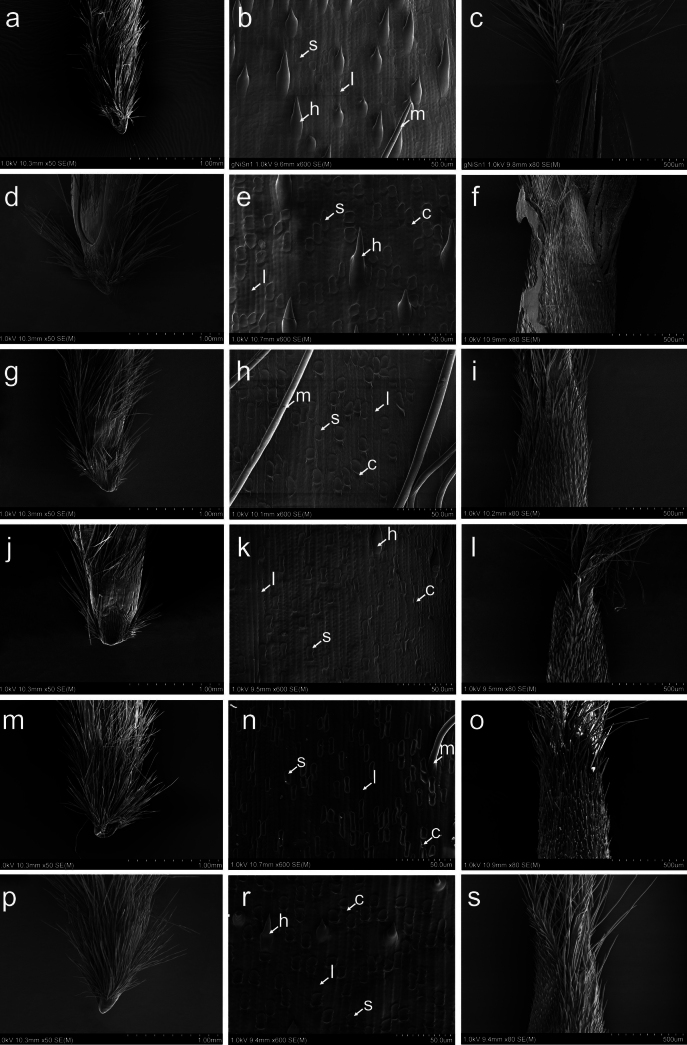
SEM morphology of the floret of *Ptilagrostisconcinna* (**a–c**), *P.dichotoma* (**d–f**), *P.junatovii* (**g–i**), *P.luquensis* (**j–l**), P.mongholicasubsp.mongholica (**m–o**) and P.mongholicasubsp.porteri (**p–s**). Callus and the lower part of the lemma (**a, d, g, j, m, p**), epidermal pattern of the middle part of lemma (**b, e, h, k, n, r**), top of lemma (**c, f, i, l, o, s**). Abbreviations: c = cork cell, h = hook, l = long cell, s = silica body, m = macro-hair.

All of the studied species representing sections *Barkworthia*, *Chenella* and *Ptilagrostis* possessed a saw-like lemma epidermal pattern. The majority of cells that build the upper (abaxial) lemma epidermis are long cells with a rectangular, oblong shape (Figs 4b, e, h, k, n, r, 5b, e, h, k, n, r). The side walls of the long cells are raised with Ω-shaped indentations. Silica bodies are rectangular to elongated in shape with sinuate edges constricted one, two or (less commonly) three times. Silica bodies are usually adjacent to cork cells, these latter being more or less frequently distributed than the silica bodies. Hooks are heterogeneously distributed, frequently near the base and the apex of the lemma, and usually absent in the middle section. Moving closer to the lemma apex, the hooks turn into prickles. Macro-hairs are straight and cylindrical (Figs 4a, d, j, m, p, 5a, d, j, m, p). In all the species studied, the lemma, beyond its central part, is covered with macro-hairs. The only exceptions are *P.glabrifolia* and *P.tibetica*, which have hairs distributed throughout the lemma. The hairs reach the top of the lemma, forming there an irregular corolla of hairs (Figs 4c, f, i, l, o, s, 5c, f, i, l, o, s).

### ﻿Leaf cross-section analysis

**Figure 6.**
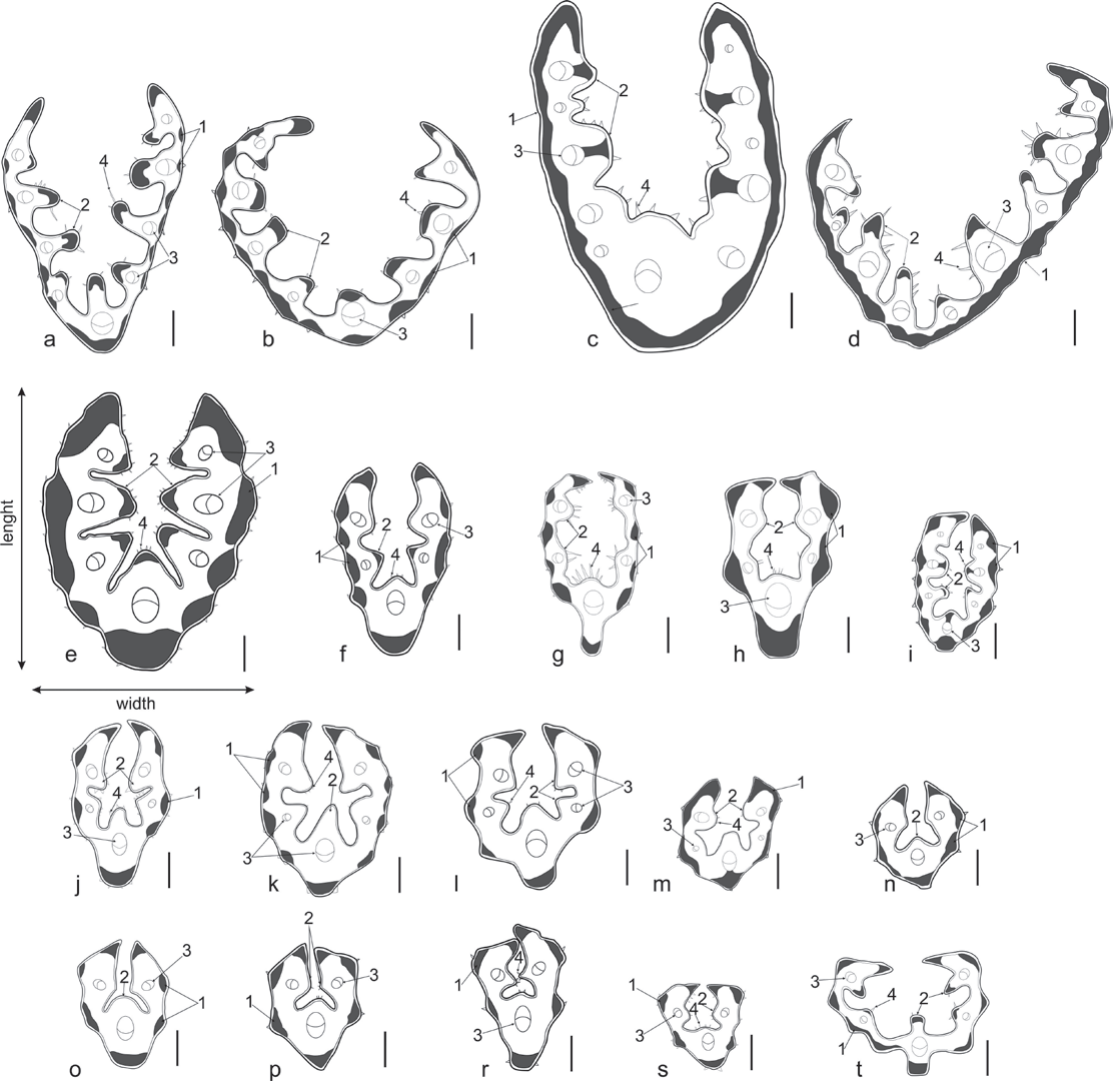
Cross-sections through vegetative leaves of *P.duthiei* (**a, b**), *P.contracta* (**c, d**), *P.malyschevii* (**e**), *P.alpina* (**f**), *P.chingii* (**g, h**), *P.bhutanica* (**i**), *P.junatovii* (**j**), *P.arcuata* (**k**), *P.concinna* (**l**), *P.yadongensis* (**m, n**), *P.luquensis* (**o**), P.mongholicasubsp.mongholica (**p**), *P.dichotoma* (**r**), *P.tibetica* (**s**), *P.glabrifolia* (**t**). Abbreviations: (1) sclerenchyma strand, (2) ribs, (3) vascular bundles, (4) prickle-hairs. Scale bars: 100 µm. List of studied specimens is presented in Suppl. material 1.

The examined *Ptilagrostis* species differ in the number of vascular bundles present in the vegetative leaf and the number of ribs on its adaxial (upper) surface. Species in sect. Barkworthia had between three and seven vascular bundles with *P.bhutanica* usually possessed seven (rarely five) (Fig. 6i), and *P.yadongensis* with three or five (Fig. 6m, n). Species belonging to sect. Chenella usually had the highest number of vascular bundles. *Ptilagrostisduthiei* had nine (less frequently 7, 8 or 11; Fig. 6a, b), while the specimens of *P.contracta* had 11–14 vascular bundles (Fig. 6c, d). The only exception in section Chenella was *P.chingii* with five (rarely three) vascular bundles (Fig. 6g, h). Among the core Ptilagrostis species (sect. Ptilagrostis), *P.malyschevii* (Fig. 6e) and *P.alpina* (Fig. 6f) have the largest number of vascular bundles. Seven (rarely five) vascular bundles were recorded in specimens of *P.malyschevii* (Fig. 6e). In contrast, five vascular bundles were most common in *P.alpina*, with seven occurring occasionally (Fig. 6f). The leaves of *P.malyschevii* are noticeably wider in comparison with the remaining species of sect. Ptilagrostis. *Ptilagrostisconcinna* (Fig. 6l), *P.arcuata* (Fig. 6k) and *P.junatovii* (Fig. 6j) are characterised by having a very similar pattern of leaf cross-sections with the presence of three to five vascular bundles, however, having three is more common in *P.arcuata* than in the cases of the other species mentioned. Three vascular bundles were present in both P.mongholicasubsp.mongholica (Fig. 6p) and P.mongholicasubsp.porteri. *Ptilagrostisdichotoma* (Fig. 6r) had three or rarely five vascular bundles. Almost all specimens of *P.glabrifolia* (Fig. 6t) had five vascular bundles with one exception that had three. Specimens of *P.tibetica* mostly had three (rarely 5) vascular bundles (Fig. 6s), whereas *P.luquensis* had three or five vascular bundles (Fig. 6o). In all studied species, short hairs or prickle-hairs were observed on the adaxial surface of leaves (on the ribs), while the abaxial surface was covered usually by more or less densely distributed prickles and thus, they were scabrous. Some specimens of *P.chingii* occur with glabrous or almost glabrous abaxial surface of leaves (as opposed to normally being scabrous). *Ptilagrostisjunatovii* and *P.luquensis* usually has glabrous leaves or rarely slightly scabrous. Whereas *P.concinna*, *P.glabrifolia* and *P.contracta*, have always glabrous and smooth leaves.

The widest leaves were noted in *P.contracta* and *P.duthiei* (0.6–1.2 mm; Table 5), while the narrowest in *P.arcuata*, *P.chingii*, *P.dichotoma*, *P.glabrifolia*, *P.luquensis*, P.mongholicasubsp.mongholica, P.mongholicasubsp.porteri, *P.tibetica*, and *P.yadongensis* (0.2–0.5 mm). In the remaining analysed species, the width of leaves ranged from 0.3 to 0.7 mm (Table 5).

**Table 5.** Morphological characters of leaves of the analysed *Ptilagrostis* species.

Species	Width of the leaf (mm)	Number of vascular bundles	Character of abaxial leaf surface	Character of sclerenchyma layer
*P.alpina*	0.3–0.5	5(–7)	slightly scabrous	discontinuous
*P.arcuata*	0.2–0.3	3–5	scabrous	discontinuous
*P.bhutanica*	0.4–0.6(–0.7)	(5–)7	scabrous or rarely glabrous	discontinuous
*P.chingii*	(0.2–)0.3–0.4 (–0.5)	3–5	scabrous or rarely glabrous	discontinuous
*P.concinna*	0.3–0.6	(3–)5	glabrous and smooth	discontinuous or occasionally continuous
*P.contracta*	(0.6–)0.7–1.2	11–14	glabrous and smooth	continuous or slightly discontinuous (torn)
*P.dichotoma*	(0.2–)0.3–0.4	3(–5)	scabrous rarely slightly scaberulous to almost glabrous (but not smooth)	discontinuous
*P.duthiei*	(0.5–)0.6–1.0	(7–)9–11	glabrous or less frequently minutely scabrous	discontinuous
*P.glabrifolia*	0.25–0.4	(3–)5	glabrous and smooth	discontinuous
*P.junatovii*	0.3–0.7	(3–)5	glabrous, rarely somewhat scaberulous	discontinuous
*P.luquensis*	0.2–0.4	3(–5)	glabrous, rarely somewhat scaberulous	discontinuous
*P.malyschevii*	0.4–0.6(–0.7)	(5–)7	scabrous	discontinuous
P.mongholicasubsp.mongholica	0.3–0.5	3	scabrous	discontinuous
P.mongholicasubsp.porteri	0.3–0.5	3	scabrous	discontinuous
*P.tibetica*	0.2–0.35	3(–5)	scabrous	discontinuous
*P.yadongensis*	0.3–0.4(–0.5)	3–5	scabrous or rarely glabrous	discontinuous

Most of the studied species had discontinuous sclerenchyma strands (Fig. 6a, b, e–n). Occasionally, an almost continuous, thin layer of sclerenchyma was observed in *P.concinna*. The only species consistently with a continuous or slightly discontinuous (torn) layer of sclerenchyma was *P.contracta* (Fig. 6c, d). A somewhat similar sclerenchyma layer was observed in *P.malyschevii*, which also has a thick layer of it, but it never merges into a continuous ring (Fig. 6e). The main morphological differences in leaves’ cross-sections between all *Ptilagrostis* species are summarised in Table 5.

### ﻿Phylogenic analyses

**Figure 7.**
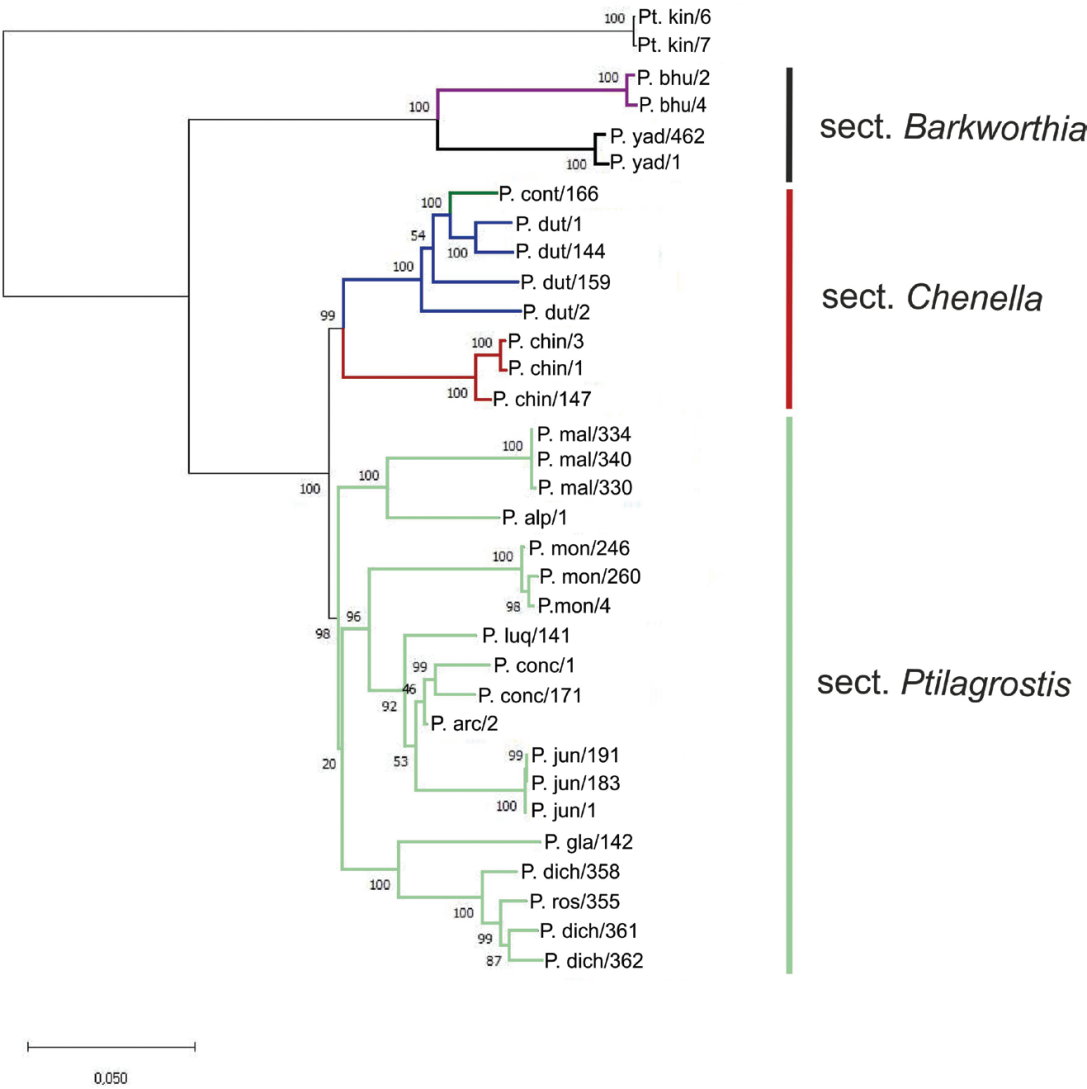
Maximum Likelihood tree based on 12,502 SNP markers derived from DArT sequencing of 16 taxa, including 15 taxa of *Ptilagrostis* and an outgroup (*Ptilagrostiellakingii*). Numbers on branches are bootstrap values. Abbreviations: *Ptilagrostisalpina* – P. alp, *P.arcuata* – P. arc, *P.bhutanica* – P. bhut, *P.chingii* – P. chin, *P.concinna* – P. conc, *P.contracta* – P. cont, P.dichotomavar.dichotoma – P. dich, P.dichotomavar.roshevitsiana – P. ros, *P.duthiei* – P. dut, *P.glabrifolia* – P. gla, *P.junatovii* – P. jun, *Ptilagrostiellakingii* – Pt. kin, *P.luquensis* – P. luq, *P.malyschevii* – P. mal, *P.mongholica* – P. mon, *P.yadongensis* – P. yad. List of samples is given in Suppl. material 1.

The Maximum Likelihood analyses based on SNPs derived from DArT sequencing revealed the arrangement of analysed representatives of *Ptilagrostis* into three distinct clades with high (98–100%) bootstrap support (Fig. 7). The first clade consists of two well supported (100% bootstrap) sister subclades representing *Ptilagrostisbhutanica* and *P.yadongensis*, which belong to the section Barkworthia. These species differ from all the remaining members of *Ptilagrostis* by having paleae distinctly shorter than lemmas. All other species of *Ptilagrostis* are grouped within large clade, divided in two sister subclades ‘*Chenella*’ and ‘*Ptilagrostis*’. The first consists of three taxa: *P.contracta*, *P.duthiei* and *P.chingii*, characterised by having awns minutely pilose on setas and the robust, usually over 60 cm tall culms (sect. Chenella). Within this subclade, specimens of *P.chingii* are well separated from the remaining samples, representing *P.duthiei* and *P.contracta*. Based on our results, the last two species are grouped together and cannot be clearly distinguished. The subclade ‘*Ptilagrostis*’ consists of nine species, *P.malyschevii*, *P.alpina*, *P.mongholica*, *P.luquensis*, *P.concinna*, *P.arcuata*, *P.junatovii*, *P.dichotoma*, and *P.glabrifolia*. Species of sect. Ptilagrostis are arranged into three well-supported (bootstrap > 96%) subclades.

**Figure 8.**
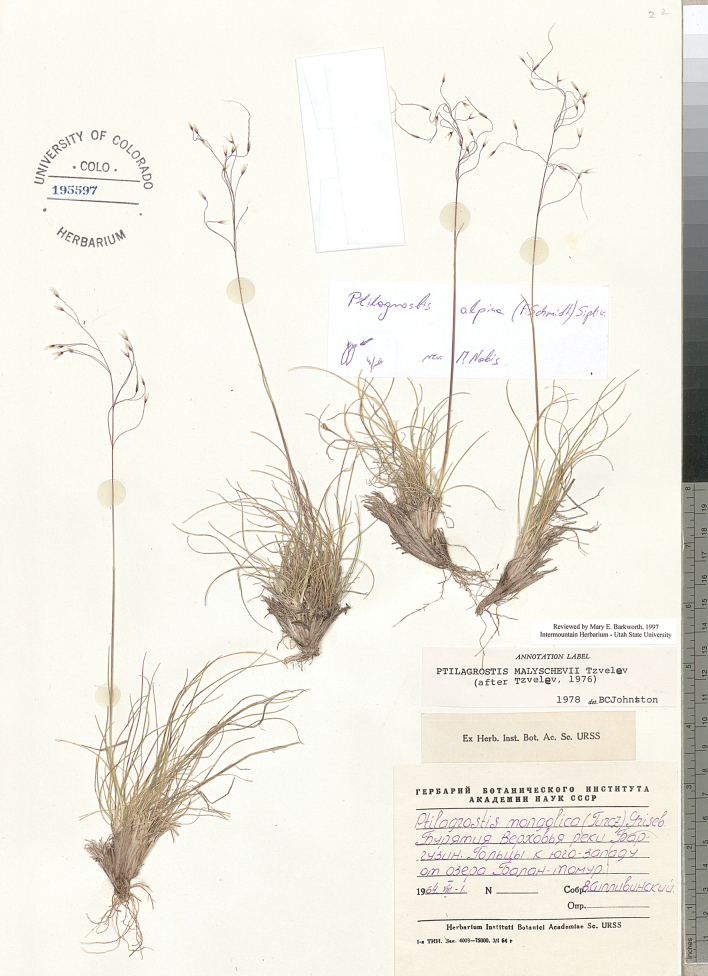
*Ptilagrostisalpina*, general habit.

### ﻿Discussion

*Ptilagrostis* is a genus with Tertiary (Miocene-Pliocene) origin, distributed almost exclusively in central and north-eastern Asia, from the Himalayas to northeastern Siberia (Tzvelev 1976, 1977; Freitag 1985; Peterson et al. 2005; Wu and Phillips 2006; Nobis and Nobis 2013; Romaschenko et al. 2014), with the Qinghai-Tibet plateau being the centre of its diversity (Zhang et al. 2017). Additionally, specimens representing *Ptilagrostis* migrated similarly as some other representatives of grasses (e.g. *Patis* Ohwi or *Eremopyrum* (Ledeb.) Jaub. & Spach) from eastern Asia via a land bridge across the Bering Strait during the Pleistocene glaciations to central North America (Johnston 2006; Romaschenko et al. 2014). Currently, only one taxon of *Ptilagrostis*, P.mongholicasubsp.porteri (=*P.porteri*), is mentioned in the North American flora (Peterson et al. 2019). The second one, namely *P.kingii*, has been recently transferred to *Ptilagrostiella* based on morphological and molecular evidence (Romaschenko et al. 2014, Peterson et al. 2019).

Some species within *Ptilagrostis* are relatively similar to each other, resulting in a limited number of morphological characters to distinguish among them. The key character most often used to differentiate particular species is the presence or absence of a tuft of hairs at the apex of anthers. However, a survey of the large number of materials has shown that specimens with glabrous anthers are occasionally also observed within specimens representing ‘bearded-anthers’-species (Zhang and Chen 2024; see also comments in the Synopsis, below). There are five species groups within *Ptilagrostis* in which particular species are morphologically similar. However, most of the species representing particular groups are well separated geographically. Examples of such geographical vicariants are *P.alpina*-*P.malyschevii*; *P.concinna*-*P.junatovii*, *P.mongholica*-*P.dichotoma* within different areas of Asia, and P.mongholicasubsp.mongholica-P.mongholicasubsp.porteri between Asia and North America. The ranges of the mentioned above high-mountain and/or cold-adapted pair of species are currently well separated due to the contraction caused by the long-term warming in the Holocene. However, it seems that local secondary contact between selected *Ptilagrostis* members was possible during glaciations, similarly as it took place in other plant species within Central Asia (Nobis et al. 2023; Vintsek et al. 2022; Wróbel et al. 2024a, 2024b), and occasional gene exchange between them could have occurred. Nevertheless, to confirm this hypothesis within particular species of *Ptilagrostis*, further studies are required.

Currently, the factor highly contributing to the species-differentiation within co-occurring species of the genus is hybridisation, which is generally a common phenomenon in plants, and often observed within grasses (Nobis et al. 2016, 2019c, 2020, 2022; Baiakhmetov et al. 2020, 2021; Sinaga et al. 2024, Wróbel et al. 2024a). Due to the generally numerous populations and the relatively wide distribution ranges for some species of *Ptilagrostis*, gene exchange among selected species has a high likelihood of occurrence. One example of such an interspecific gene flow may be *P.arcuata* (a putative hybrid species from a cross among *P.concinna* and either *P.dichotoma* or *P.luquensis*). Specimens that are possibly hybrids between *P.dichotoma* and *P.tibetica* have also been examined (e.g. *B. Dickoré 9758* and *10819*, see comments below in Synopsis). However, no hybridisation events in *Ptilagrostis* have been detected or confirmed using the molecular approach so far. This challenge will be an area of focus for our future studies.

The taxonomic and phylogenetic importance of lemma micromorphology within the Stipeae has been confirmed by many studies (Barkworth and Everett 1987; Brito 2005; Romaschenko et al. 2012; Nobis et al. 2019a, 2020; Tkach et al. 2021), and micromorphological patterns of the lemma are considered as conservative and significant, particularly at the level of genus. All of the *Ptilagrostis* species studied are characterised by having a saw-like pattern (Romaschenko et al. 2012), and more precisely *Ptilagrostis*-like LEP (Nobis et al. 2019a). In the middle part of the lemma, they have abundant silica-bodies with sinuate side-walls and numerous cork cells, however with sparse or more often without hooks (Nobis et al. 2019a, 2019b). Based on the result of studies of lemma epidermal patterns, generic affiliation of some species previously included in *Stipa* or *Achnatcherum* have been established (Nobis et al. 2016, 2019b, 2020). The epidermal patterns described above has been shown to be phylogenetically much more important than characters of the awn that were previously thought to be the key character (Nobis et al. 2019a).

Leaf cross-sections provide valuable taxonomic insights and help to differentiate species, especially those in some of the most taxonomically difficult genera, such as e.g. *Festuca*, *Stipa*, *Muhlenbergia* Schreb., and others. (Peterson et al. 1989; Martínez-Sagarra et al. 2017; Gudkova et al. 2013, 2023). However, these data were usually omitted in *Ptilagrostis* species descriptions and identification keys or mentioned only exceptionally (Malyschev 1965; Tzvelev 1974; Barkworth 1983). We found that leaf anatomy is one of the most important characters for proper identification of the species representing the sect. Barkworthia and sect. Chenella. All *Ptilagrostis* species are characterised by leaves with convoluted blades. The sclerenchyma layer and number of vascular bundles are the most distinctive characters of leaf anatomy in false needlegrasses. The sclerenchyma layer forms a continuous ring only in *P.contracta* and occasionally in *P.concinna*, while in other species it is disrupted. The highest number of vascular bundles, from (7–)9 to 11(–14), were found in *P.contracta* and *P.duthiei*. Among the remaining species, the number of vascular bundles was lower ranging from three or five. The exceptions were *P.bhutanica* and *P.malyschevii* that almost always have seven bundles.

Phylogenetic analyses revealed that *Ptilagrostis* is a well-distinguished and strongly supported clade within the Stipeae, closely related to *Neotrinia* and *Orthoraphium* (Romaschenko et al. 2012, 2014; Hamasha et al. 2012; Nobis et al. 2019a, 2020). However, both of these genera distinctly differ morphologically from *Ptilagrostis* representatives (Nobis et al. 2019a, 2019b). *Neoriniasplendens* (Trin.) M. Nobis, P.D. Gudkova & A. Nowak is distinguished by the general habit of the plant with dense tufts and tall stems, long and rigid leaves and elongated panicle with numerous flowers. Whereas *Orthoraphiumroylei* Nees possesses a unique lemma with deflexed, apical prickles (Nobis et al. 2019b). Moreover, previously performed molecular analyses of cpDNA and ITS sequences clearly separated these genera from *Ptilagrostis* (Romaschenko et al. 2008, 2012, 2014; Hamasha et al. 2012; Zhang et al. 2017). In our molecular analyses, including 14 of 15 currently distinguished false needlegrasses, the examined species were organised in three well-distinguishable clades that correspond to the three sections, *Barkworthia*, *Chenella* and *Ptilagrostis*. The species making up the clade of sect. Barkworthia (*P.bhutanica* and *P.yadongensis*) are well distinguished both morphologically and molecularly from the remaining *Ptilagrostis* species. Both aforementioned taxa have narrow panicles and paleas much shorter than lemmas.

A second clade (corresponding to sect. Chenella) included *P.duthiei*, *P.chingii* and *P.contracta*. Both *P.duthiei* and *P.chingii* have initially been described as representatives of *Stipa* (Hooker 1897; Hitchcock 1930). However, based on the morphology of the awn, they were later transferred to *Achnatherum* (Keng 1976; Kuo and Lu 1987). According to Wu and Phillips (2006), *P.duthiei* and *P.chingii* are morphologically fairly similar, and the only feature to distinguish them from each other is the length of the anthers (2.8–4.5 vs. 2–2.5 mm respectively). However, our studies revealed that anther lengths overlap one another. Anther length of *P.duthiei* is 3–4 mm, while anther length of *P.chingii* is 2.0–3.3 mm. Our data has shown that the best characters to distinguish these species are the width of vegetative leaves and number of vascular bundles on the cross-sections of leaves. In *P.duthiei*, leaves are noticeably wider and have more vascular bundles than in *P.chingii*. However, the species descriptions by Wu and Phillips (2006) indicated that leaf width is 0.5–1 mm in both species resulted in frequent misidentification of the two species. The third species of sect. Chenella, *P.contracta*, was recently described (Zhang et al. 2017) and is a strongly supported member within sect. Chenella most similar to *P.duthiei*. Our studies show that these two closely related species are differentiated by a contracted (*P.contracta*) vs. open panicle (*P.duthiei*). In comparison to *P.duthiei*, *P.contracta* also has slightly wider vegetative leaves, 0.5–1.2 mm wide, with 9–14 vascular bundles visible on the cross-section, and continuous or slightly discontinuous (torn) ring of sclerenchyma. Our molecular analyses reveal that our one representative of *P.contracta* is paraphyletic within a clade with representatives of *P.duthiei*. Further analysis with additional representatives of *P.contracta* are needed to more clearly define the phylogenetic relationships between the two species.

The third clade included all the remaining species belonging to the sect. Ptilagrostis. Within this clade, three further subclades comprising morphologically similar species can be distinguished. The first one, *Ptilagrostisalpina*-*P.malyschevii* subclade, consists of a pair of morphologically very similar species. Both of them have open panicles, fairly broad leaves with five to seven vascular bundles. Another subclade consists of *P.dichotoma* and *P.glabrifolia* (and *P.tibetica*, absent in our molecular, but present in morphological analysis) comprising morphologically very similar species, characterised by having open panicles, and filiform leaves with three or five vascular bundles. Central, the most numerous in species subclade includes: *P.concinna*, *P.junatovii*, *P.arcuata*, *P.luquensis* and *P.mongholica*. The species organisation within this subclade is somewhat puzzling. *Ptilagrostisconcinna*, *P.junatovii* and *P.arcuata* are characterised by clearly contracted panicles, which enables them to be easily distinguished from the other species of this subclade. The presence of putative hybrids (*P.arcuata*) and possible gene flow events among species of this subclade seems to be the most probable explanation for this arrangement. However, further analyses on the population level using tools of integrative taxonomy are needed to reveal the evolutionary history of this group of taxa.

### ﻿An identification key to species of *Ptilagrostis*

**Table:** 

1	Upper segment of the awn (seta) minutely pilose to scabrous, hairs near the geniculation 0.2(–0.3) mm long; culms usually ≥ 50 cm tall (sect. Chenella)	**2**
–	Upper segment of the awn (seta) pilose, hairs near the geniculation (0.3–)0.5–3.0 mm long; culms usually ≤ 50 cm tall	**4**
2	Vegetative leaves narrow, filiform, (0.2–)0.3–0.4(–0.5) mm wide; leaf vascular bundles 3–5; lemma apical lobes 0.5–1.0 mm long	***P.chingii***
–	Vegetative leaves wider, (0.5–)0.6–1.2 mm wide; leaf vascular bundles 9 (rarely 7, 11 or more); lemma apical lobes 0.2–0.3(–0.6) mm long	**3**
3	Panicle open; leaf cross-section with discontinuous (interrupted) sclerenchyma strand; anthers 3–4 mm long	***P.duthiei***
–	Panicle contracted; leaf cross-section with continuous sclerenchyma strand; anthers 2.5–3.0 mm long	***P.contracta***
4	Lemma and palea distinctly unequal, lemma 0.5–2.0 mm longer than palea; awn upper segment with 0.3–0.9 mm long hairs (sect. Barkworthia)	**5**
–	Lemma and palea equal or subequal; awn upper segment with 1–3 mm long hairs (sect. Ptilagrostis)	**6**
5	Awn upper segment near geniculation with hairs 0.6–0.9 mm long; glumes distinctly unequal, lower glume 1.8–2.5 mm longer than the upper glume; lemma 0.7–1.3 mm longer than palea; leaf vascular bundles 3–5	***P.yadongensis***
–	Awn upper segment near geniculation with hairs 0.3–0.5 mm long; glumes subequal, the lower 0.2–0.5(–0.8) mm longer than the upper), lemma 1.2–2.0 mm longer than palea; leaf vascular bundles 7	***P.bhutanica***
6	Glumes 2.6–3.5(–4.0) mm long; floret (lemma+callus) 2.2–2.7(–3.0) mm long; awn 7–13 mm long; anthers 1.0–1.4 mm long, glabrous at the apex	***P.luquensis***
–	Glumes 4.0–12.5 mm long; floret 3–8 mm long; awn 7–52 mm long; anthers 1.2–4.0 mm long, glabrous or hairy at the apex	**7**
7	Panicle contracted, 1–2 cm wide; panicle branches 0.3–2.8 cm long, suberect or narrowly ascending	**8**
–	Panicle open, 3–10 cm wide; panicle branches 2–6 cm long, spreading	**10**
8	Leaves filiform, 0.2–0.3 mm in wide, slightly scabrous; awn 8–14 mm long, without or with hardly visible geniculation; upper segment of the awn (seta) usually arcuate	***P.arcuata***
–	Leaves 0.3–0.7 mm in wide, glabrous and smooth; awn 9–20 mm long, geniculate, upper segment of the awn (seta) straight, arcuate or flexuous	**9**
9	Membranous bracts at base of panicle present and persistent; awn 8–15 mm long	***P.concinna***
–	Membranous bracts at base of panicle absent, impermanent, caducous or sporadically residual; awn 12–20 mm long	***P.junatovii***
10	Lemma covered by scattered long hairs throughout	**11**
–	Lemma covered by long hairs basally, glabrous in the middle, and pilose or scabrous apically	**12**
11	Leaves distinctly scabrous; leaf vascular bundles in cross-section 3 (rarely 5); glumes purple near base, straw coloured near apex; anthers glabrous	***P.tibetica***
–	Leaves glabrous and smooth; leaf vascular bundles in cross-section 5 (rarely 3); glumes dark purple almost throughout; anthers with a tuft of hairs at apex (rarely glabrous)	***P.glabrifolia***
12	Anthers apex glabrous (plants of north-central Asia and North America)	**13**
–	Anthers apex with a tuft of hairs (plants of south-central Asia)	**14**
13	Awn (13–)15–26(–33) mm long; floret (3.5–)4.0–5.5(–6.0) mm long (plants from Asia)	**P.mongholicasubsp.mongholica**
–	Awn 10–20 mm long; floret 3.0–4.0(–4.7) mm long (plants from N America)	**P.mongholicasubsp.porteri**
14	Vegetative leaves (0.4–)0.5–0.6(–0.7) mm in diameter; leaf vascular bundles 5–7; awn 15–52 mm long	**15**
–	Vegetative leaves 0.2–0.3(–0.4) mm in diameter, filiform; leaf vascular bundles 3 (rarely 5); awn 9–20 mm long	***P.dichotoma***
15	Panicle usually with 15–25 spikelets; panicle branches glabrous; awn (20–)28–52 mm long; floret 4.0–5.2 mm long; glumes (4.5–)5.0–6.5(–7.0) mm long; leaf vascular bundles 7 (rarely 5)	***P.malyschevii***
–	Panicle with 7–13 spikelets; panicle branches scabrous; awn 15–30 mm long; floret 3.4–4.5 mm long; glumes 4.3–5.2 mm long; leaf vascular bundles 5 (rarely 7)	***P.alpina***

### ﻿Synopsis of *Ptilagrostis*

#### ﻿ Ptilagrostis

Taxon classificationPlantaePoalesPoaceae

Griseb., Flora Rossica 4(13): 447. 1852.

9952D5A5-EABA-55D7-A4F8-AFDEC1859663

##### Type.

*Ptilagrostismongholica* (Turcz. ex Trin.) Griseb. [basionym: *Stipamongholica* Turcz. ex Trin.].

#### ﻿ Ptilagrostis sect. Ptilagrostis

Taxon classificationPlantaePoalesPoaceae

A1296367-6BCE-5A97-AB48-2ADB2D10A9A8

##### Type.

*P.mongholica* (Turcz. ex Trin.) Griseb.

##### Description.

Species belonging to this section are characterised by equal or subequal glumes, lemmas and paleas comparable in length, and awns covered by hairs > 1 mm long (usually 1–3 mm long).

#### ﻿1. Ptilagrostis alpina

Taxon classificationPlantaePoalesPoaceae

(F. Schmidt) Sipliv., Spisok Rastenij Gerbarija Flory SSSR 18: 60. 1970.

6B9BB84A-689E-557C-B281-B1D4D78EC68A

Suppl. material 2: fig. S2

Lasiagrostis alpina F. Schmidt, Reisen im Amur-Lande 73. 1868. **Basionym.**
≡ Stipaalpina (F. Schmidt) Roshev., Izvestiya Imperatorskogo Botanicheskogo Sada Petra Velikago 14(Suppl. 2): 50. 1915.
≡ Stipamongholicafo.alpina (F. Schmidt) Roshev., Flora Aziatskoi Rossii 1(12): 132. 1916.
≡ Stipaalpina (F. Schmidt) Petrov, Flora Iakutiae 1: 136. 1930.

##### Type.

‘in protologue: Auf dem kahlen Berge an der Burej-Quelle, 3 Juli’. Lectotype designated by Tzvelev 1976: 566, Ad fluv. Amur, fontes Burejae, 4 July 1859, *F. Schmidt* s.n. (LE01009402!).

##### Description.

***Perennial plants***, densely tufted, with a few culms and numerous vegetative shoots; culms 15–35 cm tall. ***Leaves of vegetative shoots***: sheaths glabrous; ***ligules*** lanceolate, the longest 0.8–1.9 mm; ***blades*** slightly scabrous, filiform, convolute, green, pale green to greyish, 0.3–0.5 mm in diameter, with 5(–7) vascular bundles. ***Cauline leaves***: sheaths glabrous or minutely scabrous; ***ligules*** on the lower sheaths lanceolate. ***Panicle*** open, (3–)5–12(–15) cm long and 3–8 cm wide, with 7–13 spikelets; ***branches*** scabrous. ***Glumes*** subequal, purple, (3.6–)4.2–5.2 mm long, lanceolate. ***Floret*** (=anthecium, =lemma + callus) 3.4–4.5 mm long. ***Callus*** 0.3–0.5 mm long, densely pilose; callus base obtuse. ***Lemma*** coriaceous, pale-green, purplish or brownish, covered from the bottom up to 1/3 of its length, by dense ascending to appressed hairs, hairless in the mid-length and with hairs at apex; ***lemma apex*** with two lobes. ***Awn*** 15–28(–30) mm long, unigeniculate; ***the lower segment of the awn* (*column*)** 5–10 mm long, twisted, with 1.2–1.6 mm long hairs; ***terminal segment of the awn* (*seta*)** straight, 10–17 mm long, with 1.2–1.6 mm long hairs, gradually decreasing in length towards the apex. ***Anthers*** 1.3–2.5 mm long, with a tuft of hairs at the apex.

##### Phenology.

Flowering from July to August.

##### Figures.

Figs 6f, 8, https://www.gbif.org/species/4149846.

##### Distribution.

Eastern Asia: Russia: eastern Siberia nad Russin Far East, NE China?, Japan? (Tzvelev 1976; Probatova 1985; Probatova et al. 2006; Tzvelev and Probatova 2019).

##### Habitat.

Grasslands and stony slopes, at 900–2200 m elev.

##### Selected studied specimens of *P.alpina*.

Russia • Ad fluv. Amur, fontes Bure­jae; 4 July 1862 [1859?]; *F. Schmidt* s.n. (syntype: LE01009403) • Khabarovskiy territory, Ayano-Mayskiy district; headwater of river Magej (180 km to S of village Nelkan); valley, edge of the seasonal melted glacier, conspicuous; 900 m elev.; 10 Aug 1978; *S. Kharkevich, T. Buch 1089* (MO-4886943, NY) • Buryatiya, Verkho­vya reki, Barguzin, Goltsy k yugo-zapadu ot ozera Balan-tomur; 1 Aug 1964; *V. Siplivinskiy* s.n. (COLO195597) • Sakhalin, Vostochno-Sakhalinskie gory, gornyi massiv g. Vodorazdelnaya (1428.7 m) - g. Nevelskogo (1397.8 m); 19 Jul 1988; *I.B. Vyshin, V.J. Barkalov* s.n. (LE) • Tsentral. Sakhalin, yug Vostochno-Sakha­linskogo Khrebta, izvestnyakovaya g. Vaida, v verkh. r. Vitnitsy; travyanistyi kru­toi sklon pod skalami; 20 Aug.2006; *V.J. Barkalov, V.V. Yakubov* s.n. (LE).

#### ﻿2. Ptilagrostis arcuata

Taxon classificationPlantaePoalesPoaceae

Z.S. Zhang & W.L. Chen, Phytotaxa 269(2): 232. 2016.

D5FB4AB2-E810-5BF0-B395-CDC6FD7DDD34

Suppl. material 2: fig. S3

=? P.pugeensis X.Y Zhang & W.L. Chen, Botanical Journal of the Linnean Society 206: 76. 2024. Type: China. Sichuan: Puge County, 267 Luoji Mountain, 27.579°N, 102.361°E, 4017 m, 1 Oct 2014, *Z.S. Zhang & L.L. Li 380-1* (holotype: PE).

##### Type.

China, Yunnan: Dêqên, Bai-Ma mountain, 4366 m, 15 Sep 2014, *Z.S. Zhang & L.L. Li 217* (holotype PE; isotype: US, K1222374!, K1222375!)

##### Description.

***Perennial plants***, densely tufted, with a few culms and numerous vegetative shoots; culms 15–50(–60) cm tall. ***Leaves of vegetative shoots***: sheaths glabrous; ***ligules*** lanceolate; ***blades scabrous***, filiform, convolute, green, pale green to greyish, 0.2–0.3 mm in diameter, with 3–5 vascular bundles. ***Cauline leaves***: sheaths glabrous or minutely scabrous; ***ligules*** on the lower sheaths lanceolate. ***Panicle*** contracted, 5.5–11 cm long and 1–2 cm wide; ***branches*** glabrous. ***Glumes*** subequal, purple, 5.0–6.6 mm long, lanceolate. ***Floret*** (lemma + callus) (4.0–)4.5–5.7 mm long. ***Callus*** 0.4–0.5 mm long, densely pilose; callus base obtuse. ***Lemma*** coriaceous, pale-green, purplish or brownish, covered from the bottom up to 1/3 of its length, by dense ascending to appressed hairs, hairless in the mid-length and with hairs at apex; ***lemma apex*** with two lobes. ***Awn*** 8–14 mm long, unigeniculate; ***the lower segment of the awn* (*column*)** 4–5 mm long, twisted, with 1.2–2.0 mm long hairs; ***terminal segment of the awn* (*seta*)** straight, 7–9 mm long, with 1.2–1.9 mm long hairs, gradually decreasing in length towards the apex. ***Anthers*** 1.2–2.1 mm long, with a tuft of hairs or sporadically glabrous at the apex.

##### Phenology.

Flowering from August to October.

##### Figures.

Fig. 6k; additional figures in Zhang et al. (2016a: figs 1, 2); https://powo.science.kew.org/taxon/urn:lsid:ipni.org:names:77157281-1.

##### Distribution.

China: Sichuan, Yunnan, Xizang and Nepal (Zhang et al. 2016a; Zhang and Chen 2024).

##### Habitat.

Alpine meadows, thickets, grassy mountain slopes, moors by the river, at 3900–4600(–4900) m elev.

##### Note.

It is not excluded that *P.arcuata* is a product of spontaneous hybridisation between *P.concinna* and *P.luquensis* or *P.dichotoma*. The origin of this taxon needs further study. Recently Zhang and Chen (2024) described a new taxon, *P.pugeensis*, morphologically similar to *P.arcuata*, and known from only one locality. The distinctiveness of this taxon requires molecular confirmation.

##### Selected studied specimens of *P.arcuata*.

China • SE Tibet, Xizang: Nyaingentanglha Shan. Yangbajain - Damxung, NW of Lhasa, Valley SE of Nyaingentanglha Feng, *Kobresia* spp. - moor by the river, alpine, elev. 4880 m, 30°18'N, 90°38'E, 11 Aug 1989, *B. Dickoré 3761* (MSB-152861) • Namchabarwa NW slope, Village “Pei No. 4” - Nam La Tso, lower alp. moist *Kobresia* meadow and *Rhododendron* dwarf-scrub among gneiss boulders, elev. 4430 m, 29°35'N, 94°59'E, 15 Sep 1989, *B. Dickoré 5352* (MSB-152913).

#### ﻿3. Ptilagrostis concinna

Taxon classificationPlantaePoalesPoaceae

(Hook. f.) Roshev., Flora Unionis Rerumpublicarum Sovieticarum Socialisticarum 2: 75. 1934.

C6D4520A-D413-5A15-87ED-42CEEE85D273

Suppl. material 2: fig. S4

Stipa concinna Hook. f., The Flora of British India 7(22): 230. 1897(1896). **Basionym**

##### Type.

Sikkim Himalaya, in the Tibetan region, 14-16000 ft, 11 Sept. 1849, *J.D. Hooker s.n.* (holotype: K!; isotypes: LE00009287!, CAL 2351!, GOET006941!, W!, P02240692)

##### Description.

***Perennial plants***, densely tufted, with a few culms and numerous vegetative shoots; culms (5–)10–30(–40) cm tall. ***Leaves of vegetative shoots***: sheaths glabrous; ***ligules*** lanceolate; ***blades*** glabrous and smooth, convolute, green, pale green to greyish, 0.3–0.6 mm in diameter, with (3–)5 vascular bundles. ***Cauline leaves***: sheaths glabrous or minutely scabrous; ***ligules*** on the lower sheaths lanceolate. ***Panicle*** contracted, 2–6 cm long and 1–2 cm wide; ***branches*** glabrous, lowest branches enclosed by a sheathing lanceolate membranous bract. ***Glumes*** subequal, purple, 4.0–7.7 mm long, lanceolate. ***Floret*** (lemma + callus) 3.3–4.5(–5.0) mm long. ***Callus*** 0.3–0.6 mm long, densely pilose; callus base obtuse. ***Lemma*** coriaceous, pale-green, purplish or brownish, covered from the bottom up to 1/3 of its length, by dense ascending to appressed hairs, hairless in the mid-length and with hairs at apex; ***lemma apex*** with two lobes. ***Awn*** (8–)10–13(–15) mm long, unigeniculate; ***the lower segment of the awn* (*column*)** 3.0–5.0(–6.5) mm long, twisted, with 1.0–1.9 mm long hairs; ***terminal segment of the awn* (*seta*)** straight, 7.0–8.0(–9.5) mm long, with 1.0–1.9 mm long hairs, gradually decreasing in length towards the apex. ***Anthers*** 1.5–2.5 mm long, with a tuft of hairs or rarely glabrous at the apex.

##### Phenology.

Flowering from July to September.

##### Figures.

**Figure 9.**
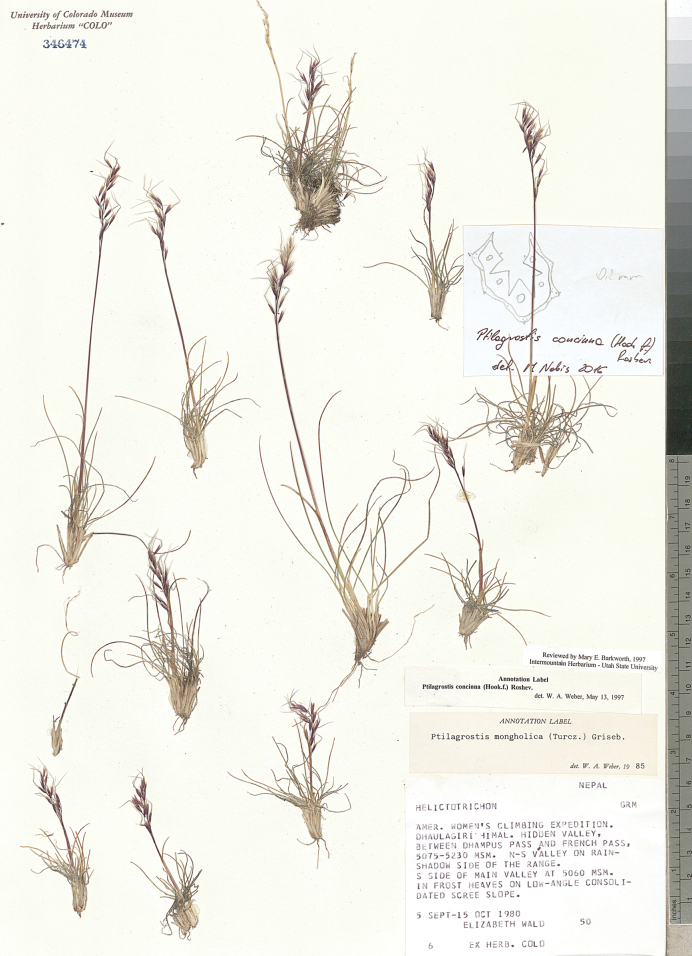
*Ptilagrostisconcinna*, general habit.

Figs 5a–c, 6l, 9; additional figures in Wu et al. (2007: fig. 279); https://www.gbif.org/species/4149833, https://powo.science.kew.org/taxon/urn:lsid:ipni.org:names:418941-1, http://www.efloras.org/object_page.aspx?object_id=95534&flora_id=2.

##### Distribution.

South and south-eastern Central Asia, in China, India and Nepal (Tzvelev 1968; Freitag 1985; Wu and Phillips 2006; Nobis et al. 2019a).

##### Habitat.

It grows on alpine mats, meadows, moist grassy places, swamps, shrubs and *Kobresia* moors, at 3500–5500 m elev.

##### Note.

Within the range of *P.concinna*, specimens with anthers glabrous at the apex are sporadically noted. Sometimes, specimens with glabrous and bearded anthers co-occur in the same locality or even in the same population. Specimens with glabrous anthers are observed also within other *Ptialgrostis* species that are characterised by having anthers bearded at the apex such as *P.concinna*, *P.junatovii* or *P.glabrifolia*. Tzvelev (1974) was the first who distinguished specimens with glabrous anthers as Ptilagrostisconcinnasubsp.schischkinii. Later on, this taxon was raised to the rank of species by Czerepanov (1981). Further molecular studies are required to determine the systematic position of these ‘glabrous-anthers’ specimens. Until then, such specimens may be distinguished, in our opinion, at most in the rank of variety. It must be mentioned, however, that after careful examination of the specimen, described by Tzvelev (1974) as Ptilagrostisconcinnasubsp.schischkinii, we found that it has no bracts at the bottom of the panicle, thus morphologically it is more similar to *P.junatovii* than to *P.concinna*. Its occurrence (northern Central Tian Shan) is also more closely located to the range of *P.junatovii* rather than *P.concinna*. Thus, we here decided to transfer it to the previous species in the rank of variety (see below). In the meantime, during the revision of the specimens of *P.concinna*, we found within the range of this species, the specimens with glabrous anthers, and well-developed bracts at the bottom of panicle. This morphotype is here recognised as a variety of *P.concinna*:

#### ﻿ Ptilagrostis concinna var. xizangensis

Taxon classificationPlantaePoalesPoaceae

M.Nobis & Krzempek var. nov.

35F85075-0893-56F9-B7AD-62624A632B79

urn:lsid:ipni.org:names:77351829-1

##### Diagnosis.

From the nominal variety it differs in having anthers glabrous at the apex (instead of bearded).

##### Type.

China • Xizang, SE.Tibet, Nyaingentanglha Shan. Yangbajain – Damxung, NW of Lhasa, S slope of Nyainqentanglha Feng, high alpine *Kobresiapygmaea*-turf on steep S-facing slope, elev. 5290 m, 30°20'N, 90°34'E, 13.08.1989. *B. Dickoré 3931* (holotype: MSB-152847, Suppl. material 2: fig. S5, isotypes: KRA528809, MSB-152860 – specimen mounted in the upper-left corner of the sheet).

##### Selected studied specimens of P.concinnavar.concinna.

China • Sichuan, reg. bor.: Dongrergo; in prato alpino aperto; elev. ca. 4800 m; 2 Jul 1922; *H. Smith 3728* (BM001031161) • Sikang, Kangting (Tachienlu) distr., Tapaoshan; ad rupes; elev. 4600–4800 m; 22 Aug 1934; *H. Smith 11474* (V-038934) • Sich­uan, Sikang, Taofu (Dawo) distr., Haitzeshan; in rupibus; elev. 4700 m; 31 Aug 1934; *H. Smith 11687* (V-047430) • Xinjiang, Kun-lun, Kashgarya, morenovyi vo­doazdel mezhdy r. r. Atraknr i Tyuzytek; mkhovaya tundra; na vysote 4500–5000 m; 20 Jul.1942; *V.I. Serpukhov 5454* (LE) • Kun-lun, verkhovya r. Tuznaf, v 3–4 km vost. per. Sarnk (217 km Tibetskoi shoce); 4800 m; 4 Jun 1959; *A.A. Yuna­tov, Yuan I-fen 320* (LE) • Tibet (Xizang), Bassein Yan-tsy-tszyana (r. Goluboi), pereval Cha-mu-bug-la; 15700 ft; 26 Jul 1900; *V.O. Ladygin* s.n. (LE). – India • Kash­mir, Apharwat; elev. 13,300 ft.; 12 Aug 1956; *O. Polunin 56/207* (BM001191546). Additional specimens studied are listed in Suppl. material 1.

#### ﻿4. Ptilagrostis dichotoma

Taxon classificationPlantaePoalesPoaceae

Keng ex Tzvelev, Rasteniia Tsentral’noi Azii 4: 43. 1968.

56C193FE-9385-550B-B802-8ECCD7B928D8

Suppl. material 2: fig. S6

≡ Ptilagrostisdichotoma Keng, Claves Generum et Specierum Graminearum Primarum Sinicarum Appendice Nomenclatione Systematica 213. 1957 [nom. inval., without Latin description].

##### Type.

China: Gansu and Qinghai border [in regione opp. Labrang], *Y.C. Wu 478* (holotype: NJ, isotype: LE!)

##### Description.

***Perennial plants***, densely tufted, with a few culms and numerous vegetative shoots; culms (12–)15–50(–74) cm tall. ***Leaves of vegetative shoots***: sheaths glabrous; ***ligules*** lanceolate; ***blades*** scabrous rarely slightly scaberulous to almost glabrous (but not smooth), filiform, convolute, green, pale green to greyish, (0.2–)0.3–0.4 mm in diameter, with 3(–5) vascular bundles. ***Cauline leaves***: sheaths glabrous or minutely scabrous; ***ligules*** on the lower sheaths lanceolate. ***Panicle*** open, 4–18 cm long and 3–10 cm wide; ***branches*** glabrous or rarely scabrous. ***Glumes*** subequal, purple, (3.5–)4.0–5.5(–7.0) mm long, lanceolate. ***Floret*** (lemma + callus) 3.5–5.2 mm long. ***Callus*** 0.3–0.6 mm long, densely pilose; callus base obtuse. ***Lemma*** coriaceous, pale-green, purplish or brownish, covered from the bottom up to 1/3 of its length, by dense ascending to appressed hairs, hairless in the mid-length and with hairs at apex; ***lemma apex*** with two lobes. ***Awn*** (9.0–)11.0–15.5(–20.0) mm long, unigeniculate; ***the lower segment of the awn* (*column*)** (3.0–)4.0–6.5(–8.0) mm long, twisted, with (1.3–)1.7–2.4(–3.0) mm long hairs; ***terminal segment of the awn* (*seta*)** straight, 7–10 mm long, with 1.4–2.0(–3.0) mm long hairs, gradually decreasing in length towards the apex. ***Anthers*** 1.3–2 mm long, with a tuft of hairs at the apex.

##### Phenology.

Flowering from July to September.

##### Figures.

**Figure 10.**
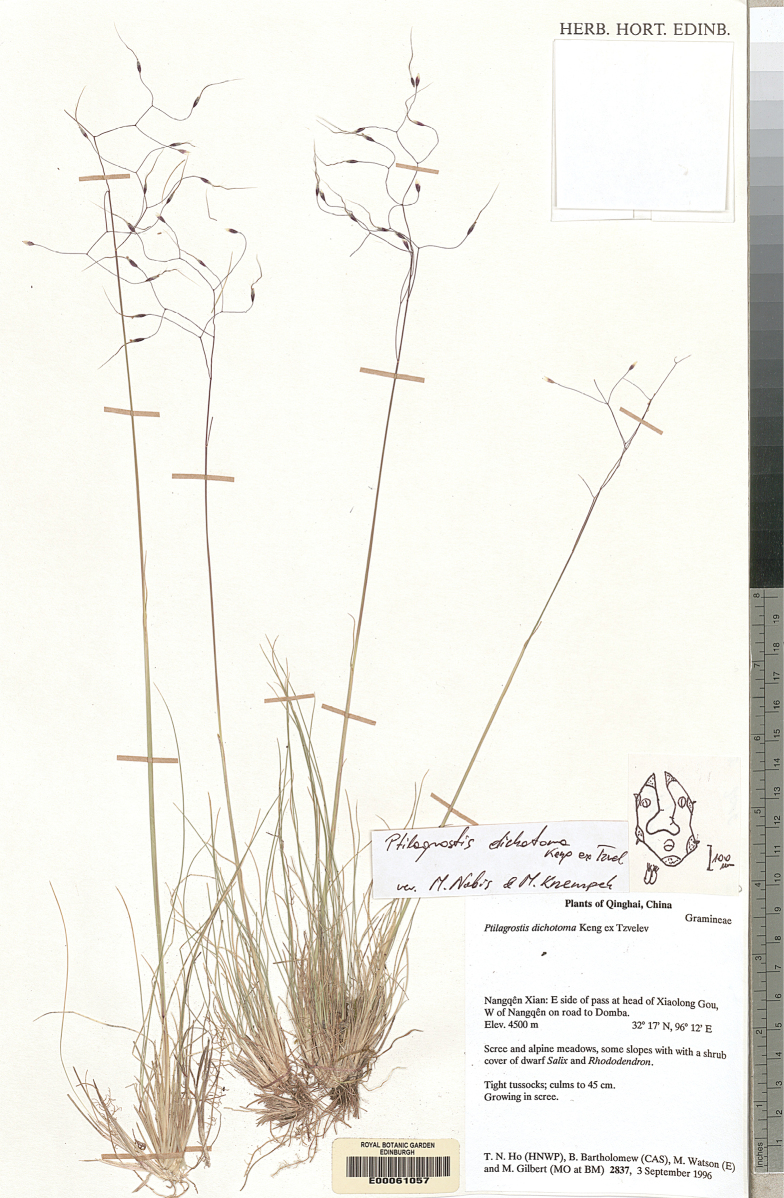
*Ptilagrostisdichotoma*, general habit.

Figs 5d–f, 6r, 10; additional figures in Wu et al. (2007: fig. 280); https://www.gbif.org/species/7325743, http://www.efloras.org/object_page.aspx?object_id=95535&flora_id=2.

##### Distribution.

The species occurs in southern Central Asia, in mountain areas of China, India, Nepal, Bhutan, and Myanmar (Wu and Phillips 2006; Nobis et al. 2019a).

##### Habitat.

Alpine meadows, bogs, mats, under shrubs, and forests, at 3000–5000 m elev.

##### Note.

This taxon is very similar to *P.mongholica* in having very narrow, filiform leaves. However, they differ from each other in length of awns (shorter in *P.dichotoma*) and characters of anthers (bearded at the top in *P.dichotoma* and glabrous in *P.mongholica*). Within the northern part of the range of *P.dichotoma*, specimens with slightly scabrous panicle branches (not glabrous as in typical specimens of nominal variety P.dichotomavar.dichotoma) were described by Tzvelev (1968) as:

**Ptilagrostisdichotomavar.roshevitsiana Tzvelev**, Rastenia Tsentral’noi Azii 4: 43. 1968. [≡ *Ptilagrostisroshevitsiana* (Tzvelev) L.B. Cai, Acta Phytotaxonomi­ca Sinica 43(1): 65–67. 2005]. **Type.** China occidentalis: prov. Kansu: in regione media sylvarum jugi Austro-Tetungensis, ca. 2800 m, 24 July 1880, *N.M. Przewalski* s.n. (holotype: LE!, Suppl. material 2: fig. S7). However, besides character of panicle branches (glabrous vs. scabrous), the two varieties mentioned above do not differ in any additional morphological character.

##### Selected studied specimens of P.dichotomavar.dichotoma.

Bhutan • Upper Mo Chu, E bank of Tharizam Chu; shady ground by stream under *Juniperus & Salix*; elev. 4080 m; 28°01'N, 89°35'E; 25 Sep 1984; *I. W. J. Sinclair & D. G. Long 5323* (E00619019). – China • Qinghai, Maqin (Maqên) Xian, Dawu Xiang, SE of Ma­qin (Maqên); on slopes, alpine meadow with Hedysarum and N facing slope with very dense turf and frequent dwarf shrubs, mostly *Potentilla*; elev. 3920 m; 34°24'11"N, 100°23'34"E; 29 Aug 1993; *T. N. Ho, B. Bartholomew, M. Gilbert 527* (MO) • Maqin (Maqên) Xian, Xihalong Guo, between Jungong (Gyumgo) and Maqin (Maqên) on S side of the Huang He; among shrubs in NW facing *Picea* woodland, *Picea* forest on N & E slopes, *Juniperus* forest on W facing slopes, alpine meadow along the valley between and at top of altitude range covered; elev. 3500–3600 m; 34°38'56"N, 100°36'38"E; 23 Jul 1993; *T. N. Ho, B. Bar­tholomew, M. Gilbert 265* (E00619219) • Yunnan, Baima Snow Mountain, Diqing; hillside meadow; elev. 4600 m; 25 Sep 1986; *H. Sun, Z. Qian 0809* (KUN0323197) • Xizang, Cona County, Mountain Pass; elev. 4500 m; 18 Jul 1975; *C. Zhengyi, Q. Du 75-997* (KUN0323186) • Sichuan, reg. bor.-occid.: ca. 45 km ad bor.-orient. ver­sus a Sankar-von-ma; in uliginosis; elev. ca. 4100 m; 4 Sep 1922; *H. Smith 4338* (MO-4366934) • Xisheng, Baizhu, Chayu County; elev. 3600–3700 m; 8 Sep 1982 (KUN0382062). Additional specimens studied are listed in Suppl. material 1.

##### Selected studied specimens of P.dichotomavar.roshevitsiana.

China • Qinghai, Huang-yuan Hsien, O-Yo; expanded northern slope; 8 Aug 1944; *Keng 5194* (H2012/01581 3) • Maqin (Maqên) Xian, Xihalong Guo, between Jungong (Gyumgo) and Maqin (Maqên) on S side of the Huang He; among shrubs in NW facing *Picea* woodland, *Picea* forest on N & E slopes, *Juniperus* forest on W facing slopes, alpine meadow along valley between and at top of altitude range covered; elev. 3500–3600 m; 34°38'56"N, 100°36'38"E; 23 Jul 1993; *T. N. Ho, B. Bartholomew, M. Gilbert 265* (MO-4648135) • Sichuan, reg. bor.: Dongrergo; in prato herboso-fruticoso; elev. 4300–4400 m; 20 Jul 1922; *H. Smith 3798* (MO-4366943, V-038623).

#### ﻿5. Ptilagrostis glabrifolia

Taxon classificationPlantaePoalesPoaceae

X.Y. Zhang & W.L. Chen, Botanical Journal of the Linnean Society 206: 79. 2024.

6AE1E82B-DE5D-51BF-AFA1-3369DFBA4CC1

##### Type.

China. Xizang: Markam 341 County, Lawu Mountain, 4326 m, 16 Sep 2021, *X.Y. Zhang & W.H. Li 395* (holotype: PE).

##### Description.

***Perennial plants***, densely tufted, with a few culms and numerous vegetative shoots; culms 20–50 cm tall. ***Leaves of vegetative shoots***: sheaths glabrous; ***ligules*** lanceolate; ***blades*** glabrous and smooth, filiform, convolute, green, pale green to greyish, 0.25–0.4 mm in diameter, with (3–)5 vascular bundles. ***Cauline leaves***: sheaths glabrous or minutely scabrous; ***ligules*** on the lower sheaths lanceolate. ***Panicle*** open, 5–11 cm long and 4–8 mm wide; ***branches*** glabrous. ***Glumes*** subequal, purple, 5.2–7.0 mm long, lanceolate. ***Floret*** (lemma + callus) (3.5–)4.6–5.7 mm long. ***Callus*** 0.4–0.6 mm long, densely pilose; callus base obtuse. ***Lemma*** coriaceous, pale-green, purplish or brownish, covered from the bottom up to 1/3 of its length, by dense ascending to appressed hairs, hairless in the mid-length and with hairs at apex; ***lemma apex*** with two lobes. ***Awn*** (10.0–)12.0–17.5(–20.0) mm long, unigeniculate; ***the lower segment of the awn* (*column*)** 6–7(–8) mm long, twisted, with 1.2–2.1 mm long hairs; ***terminal segment of the awn* (*seta*)** straight, 10.0–13.0(–14.0) mm long, with 1.0–1.7 mm long hairs, gradually decreasing in length towards the apex. ***Anthers*** 1.5–2.1 mm long, with a tuft of hairs or glabrous at the apex.

##### Phenology.

Flowering from July to September.

##### Figures.

Fig. 6t; additional figures in Zhang and Chen (2024: fig. 4).

##### Distribution.

China: Sichuan, Yunnan, Xizang, Nepal (Zhang and Chen 2024).

##### Habitat.

Alpine meadows, alpine Rhododendron thickets, at 3400–4400 m elev.

##### Note.

*Ptilagrostisglabrifolia* is the most similar to *P.tibetica*; both species have lemmas pubescent throughout, but *P.glabrifolia* has completely glabrous leaves of vegetative shoots (instead of distinctly scabrous as in *P.tibetica*), glumes almost up to the apex dark purple vs. glumes purple only in the lower half and light brown to straw-coloured in the upper half, respectively. In contrast to *P.tibetica*, *P.glabrifolia* has anthers bearded on the apex or there are at least solitary hairs (Zhang and Chen 2024). However, during our studies, we found two sheets with specimens of *Ptilagrostis* morphologically corresponding to the description of *P.glabrifolia*, but differing in having anthers completely glabrous on the apex. This morphotype is here recognised as a variety of *P.glabrifolia*:

#### ﻿ Ptilagrostis glabrifolia var. himalayensis

Taxon classificationPlantaePoalesPoaceae

M.Nobis & Krzempek var. nov.

B9B53A0E-4C73-5375-89D0-3A9BED4EE3B4

urn:lsid:ipni.org:names:77351830-1

##### Diagnosis.

The new variety differs from P.glabrifoliavar.glabrifolia in having anthers glabrous at the apex.

##### Type.

**Figure 11.**
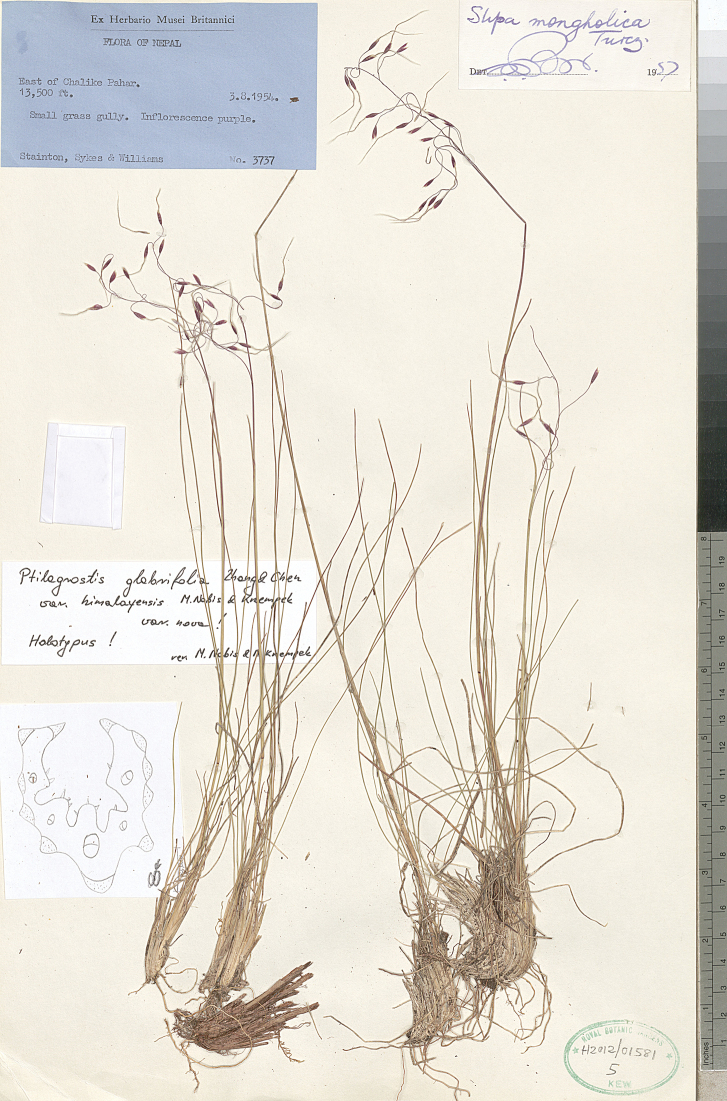
The holotype of Ptilagrostisglabrifoliavar.himalayensis.

Nepal • East of Chalike Pahar, elev. 13,500 ft., 3 Aug 1954, *Stainton, Sykes & Williams 3737* (holotype: K – H2012/0158/5, Fig. 11). Paratype: China: Yunnan, Deqin, east slope of Baima Mountain, elev. 4300–4500 m, 14 Jul 1981, *Green Team 3019* (KUN319284).

##### Selected studied specimens of P.glabrifoliavar.glabrifolia.

Nepal • Mahari­gaon; growing on grass slopes among Scripus on south aspect; elev. 15,000 ft.; 20 Jul 1952; *O. Polinin, W. R. Sykes, L. H. J. Williams 226* (H2012/0158/7) • East of Chalike Pahar; elev. 13,500 ft.; 3 Aug 1954; *Stainton, Sykes & Williams 3737* (E00619018) • Ganja La (N side); foot of scree, tussock grass growing among moss-covered boulders, with *Potentillafruticosa*, *Rhodiola* & *Kobresia* spp.; elev. 4300 m; 19 Jul 1986; G. & S. Miehe 5924 (H2012/0158/6).

#### ﻿6. Ptilagrostis junatovii

Taxon classificationPlantaePoalesPoaceae

Grubov, Botanicheskie Materialy Gerbariia Botanicheskogo Instituta Imeni V.L. Komarova Akademii Nauk SSSR 17: 3–4. 1955.

305785B4-AACB-56D5-9A3F-D82653865195

Suppl. material 2: fig. S8

##### Type.

Mongolia: Changai, jugum Tarbagatai, Dzagastuin-Daba, in partis subalpinis cariceto-cobresietis, inter fruticulos *Betularotundifolia* (MNR, Arachangayskii aimak, Tsakhir somon, khr. Tarbagatai, pereval Tszagastuin daba, vysokogornyi poyas, zarosli *Betularotundifolia*, po protalinam zanyatym osokovo-kobrezievym lugom, 8 Aug 1951; *A.A. Yunatov* s.n. (holotype LE!, isotype LE!).

##### Description.

***Perennial plants***, densely tufted, with a few culms and numerous vegetative shoots; culms (10–)15–25(–30) cm tall. ***Leaves of vegetative shoots***: sheaths glabrous; ***ligules*** lanceolate; ***blades*** glabrous and smooth rarely somewhat scaberulous, convolute, green, pale green to greyish, 0.3–0.7 mm in diameter, with (3–)5 vascular bundles. ***Cauline leaves***: sheaths glabrous or minutely scabrous; ***ligules*** on the lower sheaths lanceolate. ***Panicle*** contracted, 4–10 cm long and 1–2 cm wide; ***branches*** glabrous. ***Glumes*** subequal, purple, (5.0–)6.0–7.5 mm long, lanceolate. ***Floret*** (lemma + callus) (4.0–)4.5–6 mm long. ***Callus*** 0.4–0.7 mm long, densely pilose; callus base obtuse. ***Lemma*** coriaceous, pale-green, purplish or brownish, covered from the bottom up to 1/3 of its length, by dense ascending to appressed hairs, hairless in the mid-length and with hairs at apex; ***lemma apex*** with two lobes. ***Awn*** (12–)14–20 mm long, unigeniculate; ***the lower segment of the awn* (*column*)** 5.0–7.0 mm long, twisted, with 1.0–1.5 mm long hairs; ***terminal segment of the awn* (*seta*)** straight, 7.0–11.0 mm long, with 1.0–1.3 mm long hairs, gradually decreasing in length towards the apex. ***Anthers*** ca. 1.3–2.2 mm long, with a tuft of hairs or rarely glabrous at the apex.

##### Phenology.

Flowering from July to September.

##### Figures.

**Figure 12.**
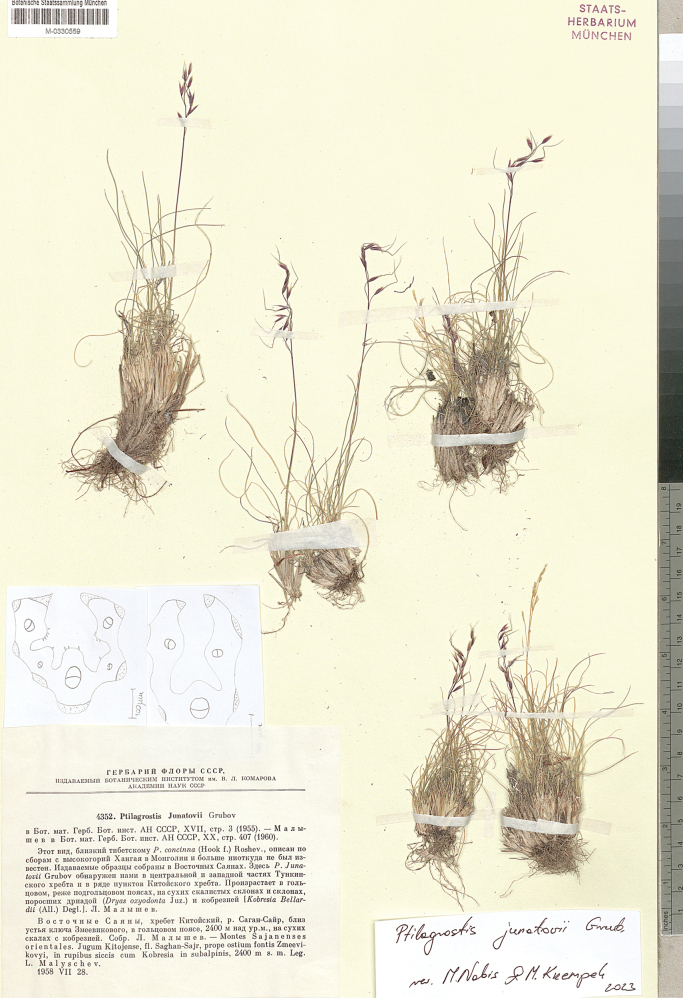
*Ptilagrostisjunatovii*, general habit.

Figs 5g-i, 6j, Fig. 12; additional figures in Wu et al. (2007: fig. 279); https://www.gbif.org/species/4149793, http://www.efloras.org/object_page.aspx?object_id=95534&flora_id=2.

##### Distribution.

The species occurs in alpine habitats in the mountain areas of North Central Asia, in Russia, Kazakhstan, Mongolia and northern China (Tzvelev 1976, Tzvelev and Probatova 2019; Wu and Phillips 2006).

##### Habitat.

Alpine mats, stony slopes, gravels, at 2200–3500(–4500) m elev.

##### Note.

In Tian Shan Mts, within the south-western part of the range of *P.junatovii*, specimens with glabrous anthers are sporadically noted. Nevertheless, besides this character, these specimens (which also do not have membranaceus bracts at the bottom of the panicle) do not differ from the typical representatives of *P.junatovii* in any additional morphological character (see also note under description of *P.concinna*). Such specimens are distinguished here as a distinct variety of *P.junatovii*.

#### ﻿ Ptilagrostis junatovii var. schischkinii

Taxon classificationPlantaePoalesPoaceae

(Tzvelev) M.Nobis & Krzempek, comb. et stat. nov.

3B8218A3-5C04-58BD-9791-5FBD83CE4B7C

urn:lsid:ipni.org:names:77351831-1

Suppl. material 2: fig. S9

Ptilagrostis concinna subsp. schischkinii Tzvelev, Novosti Sistematiki Vysshchikh Rastenii 11: 8. 1974. **Basionym.**
≡ Ptilagrostisschischkinii (Tzvelev) Czerep., Sosudistye Rasteniia SSSR 379. 1981.

##### Type.

Tien Shan: Semirechensk distr., Przhevalskii post, Kokdzhar River, subalpine meadows and slopes, 2700 m, 26 Jul 1912, *B. Sapozhnikov & B. Shishkin* s.n. (holotype: LE!, with Tzvelev’s notes: ‘Ptilagrostisconcinna(Hook f.)Roshev.subsp.tianschanica m. subsp. nova! Typus! N. Tsvelev, 1972’ and ‘Ptilagrostisconcinna(Hook f.)Roshev.subsp.schischkinii Tzvel. subsp. nova, Typus! VI.1972, N. Tzvelev’).

##### Selected studied specimens of P.junatoviivar.junatovii.

Russia • Za­padnyy Altay, khr. Ivanovskiy, ver. Vyshe Ivanovskaya; kamenistaya tundra; elev. 2600 m; 10 Jul 1981; *Yu.A. Kotukhov* s.n. (LE) • Montes Sajanenses, Ori­entales, Jugum Kitojense, fl. Saghan-Sajr, prope ostium fontis Zmeevikovyi; in rupibus siccis cum Kobresia in subalpinis; elev. 2400 m; 28 Jul 1958; *L. Malyschev 4352* (NY) • Gorna Altaisk Auton. Oblast. Kuraisky Range, north­east of Ak-Tash; alpine cirque basin and screes, above mercury mine area; elev. 2400–3000 m; 15 Jul 1987; *D. Murray, W.A. Weber, I. Krasnoborov 394* (COLO434876). – Mongolia • Zap. Prikhubs., pravoberezhe Khomon-gola, v 25 km k cev. ot Rinchin-Lkhuzhby, zap. sklon Sula-Uly, lug, 2600–2700 m, 8 Aug 1972, *V. Grubov et al. 825* (LE). Arkhaigaiskii aimak, g. Khan-undur; yuzhnui sk­lon, kobreznik; 9 Aug 1974; *Baizra[?]… et al. 6160* (LE). Additional specimens studied are listed in Suppl. material 1.

##### Selected studied specimens of P.junatoviivar.schischkinii.

China • Eastern Tian-Shan, N slope, upper Danu-gol [Danugou] river, W of Manas River, 7–8 km S of Danugou Pass; elev. 3450 m; *Kobresia*-meadow; 22 Jul 1957; *Guan 507* (LE, KRA634251).

#### ﻿7. Ptilagrostis luquensis

Taxon classificationPlantaePoalesPoaceae

P.M. Peterson, Soreng & Z.L. Wu, Sida 21(3): 1356, 1358, f. 1. 2005.

0DFC8D85-AEF4-52DA-AF6B-97060BE8C0D5

##### Type.

China • Gansu Prov: Luqu Co.: Ca. 30–40 km N of Gansu/Sichuan border on road from Chengdu to Lanzhou, ca. 20 km S of Waxu and 10 km E of Gahai, ca. 230 km SSW of Lanzhou at km post 394, 34°24'N, 102°17'E, 3440 m, 18 Sep 1997, *R. J. Soreng, P. M. Peterson & H. Sun 5383* (holotype: US; isotypes: HNWP, K!, KUN!, MO, PE).

##### Description.

***Perennial plants***, densely tufted, with a few culms and numerous vegetative shoots; culms 5–23 cm tall. ***Leaves of vegetative shoots***: sheaths glabrous; ***ligules*** lanceolate; ***blades*** abaxially glabrous and smooth (somewhat scaberulous only along keel), filiform, convolute, green, pale green to greyish, 0.2–0.4 mm in diameter, with 3(–5) vascular bundles. ***Cauline leaves***: sheaths glabrous or minutely scabrous; ***ligules*** on the lower sheaths lanceolate. ***Panicle*** loosely contracted to open, 2–6 cm long and 2–4 cm wide; ***branches*** glabrous and smooth. ***Glumes*** subequal, whitish with the base purplish, 2.6–3.5(–4.0) mm long, lanceolate. ***Floret*** (lemma + callus) 2.2–2.7(–3.0) mm long. ***Callus*** 0.2–0.3 mm long, densely pilose; callus base obtuse. ***Lemma*** coriaceous, pale-green, purplish or brownish, covered from the bottom up to 1/3 of its length, by dense ascending to appressed hairs, hairless in the mid-length and with hairs at apex; ***lemma apex*** with two lobes, 0.4–0.6 mm long. ***Awn*** 6.0–10.0(–13.0) mm long, unigeniculate; ***the lower segment of the awn* (*column*)** 2.0–4.0(–5.0) mm long, twisted, with 1.5–1.9 mm long hairs; ***terminal segment of the awn* (*seta*)** straight, 5.0–8.0 mm long, with 1.0–1.5(–1.7) mm long hairs, gradually decreasing in length towards the apex. ***Anthers*** 1.0–1.4 mm long, glabrous at the apex.

##### Phenology.

Flowering from August to September.

##### Figures.

**Figure 13.**
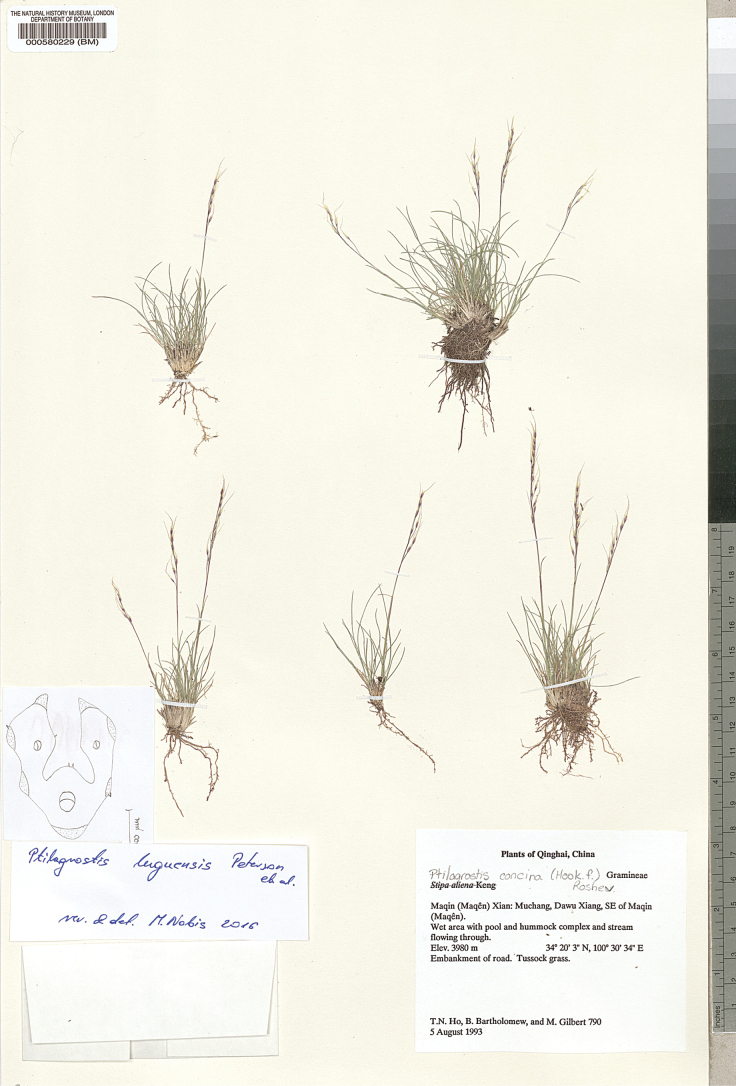
*Ptilagrostisluquensis*, general habit.

Figs 5–l, 6o, 13; additional figures in Peterson et al. (2005: fig. 1); https://www.gbif.org/occurrence/3946801294.

##### Distribution.

Mountain areas of Central Asia: China: Gansu, Qinghai, Sichuan, Xizang (Peterson et al. 2005; Wu and Phillips 2006).

##### Habitat.

Alpine meadows, at 3300–4800 m elev.

##### Selected studied specimens of *P.luquensis*.

China • Gansu, Luqu Co., ca. 30–40 km N of Gansu-Sichuan boarder on rd. from Chengdu to Lanzhou, ca. 20 km S of Waxu and 10 km E of Gahai, ca. 230 km SSW Lanzhou, k.p. 394; elev. 3440 m; 34°10'N, 102°25'E; Grassy nearly level plains surrounded by low grassy hills, in dense thatch, mollisol soils, with *Poa*, *Calamagrostis*, *Koeleria*, *Roegne­ria*, *Stipa*, *Ptilagrostis*; 18 Sep 1997; *R.J. Soreng, P.M. Peterson, Sun Hang 5383* (KUN0096489 - isotype) • Qinghai, Maqin (Maqên) Xian, Muchang, Dawu Xiang, SE of Maqin (Maqên); wet area with pool and hummock complex and stream flowing through, embankment of road; elev. 3980 m; 34°20'3"N, 100°30'34"E; 5 Aug 1993; *T.N. Ho, B. Bartholomew, M. Gilbert 790* (BM000580229, KRA628866) • Maqin (Maqên) Xian, Dawu Xiang, along the Deleni He, S of Maqin (Maqên); steep slope with semi-consolidated scree and alpine meadow in alternating strips, on grassy slope; elev. 3050 m; 34°21'54"N, 100°11'34"E; 6 Aug 1993; *T.N. Ho, B. Bartholomew, M. Gilbert 810* (BM000577764) • Huang-yuan Hsien, Hara Kutur, 14 Aug 1944, *Keng 5468* (K H2012/01581 4) • Sichuan; elev. 4000 m; 27 Aug 1987; *Qinghai-Tibet Team 4390* (KUN0319289).

#### ﻿8. Ptilagrostis malyschevii

Taxon classificationPlantaePoalesPoaceae

Tzvelev, Novosti Sistematiki Vysshchikh Rastenii 11: 7. 1974.

8ECED193-1575-549E-BD95-49DF88106962

Suppl. material 2: fig. S10

= Stipamongholicavar.barbellata Roshev., Flora Azyatskoi Rossii 12: 132. 1916 (lectotype of this taxon was selected and distinguished by Tzvelev in October 1972 as the holotype of P.malyschevii).
≡ Ptilagrostismongholicavar.barbellata (Roshev) Roshev., Flora SSSR 2: 75. 1934.

##### Type.

Kyrgyzstan, Tsentralnyi Tian-Shan, Semirechenskaya obl., Pishpekskii u., severn. sklony u r. Buzulgan, 18 July 1908, *R.J. Roshevits 1244* (holotype: LE! with note ‘*Ptilagrostismalyschevii* m. sp. nova! Typus! X.1972, N. Tzvelev; isotype LE!).

##### Description.

***Perennial plants***, densely tufted, with a few culms and numerous vegetative shoots; culms (10–)15–45(–70) cm tall. ***Leaves of vegetative shoots***: sheaths glabrous; ***ligules*** lanceolate, the longest 2.3–4.5; ***blades*** scabrous, convolute, green, pale green to greyish, 0.4–0.6(–0.7) mm in diameter, with (5–)7 vascular bundles. ***Cauline leaves***: sheaths glabrous or minutely scabrous; ***ligules*** on the lower sheaths lanceolate. ***Panicle*** open, 5–15 cm long and 4–10 cm wide, with 15–25 spikelets (in young individuals fewer than 15); ***branches*** glabrous. ***Glumes*** subequal, purple, 4.4–5.0(–6.0) mm long, lanceolate. ***Floret*** (lemma + callus) 3.8–4.6(–5.3) mm long. ***Callus*** 0.3–0.7 mm long, densely pilose; callus base obtuse. ***Lemma*** coriaceous, pale-green, purplish or brownish, covered from the bottom up to 1/3 of its length, by dense ascending to appressed hairs, hairless in the mid-length and with hairs at apex; ***lemma apex*** with two lobes. ***Awn*** (20–)28–45(–52) mm long, unigeniculate; ***the lower segment of the awn* (*column*)** (6–)10–17(–20) mm long, twisted, with 1.2–2.0 mm long hairs; ***terminal segment of the awn* (*seta*)** straight, (10–)15–25(–32) mm long, with 1.0–1.9 mm long hairs, gradually decreasing in length towards the apex. ***Anthers*** 2–3 mm long, with a tuft of hairs at the apex.

##### Phenology.

Flowering from July to September.

##### Figures.

**Figure 14.**
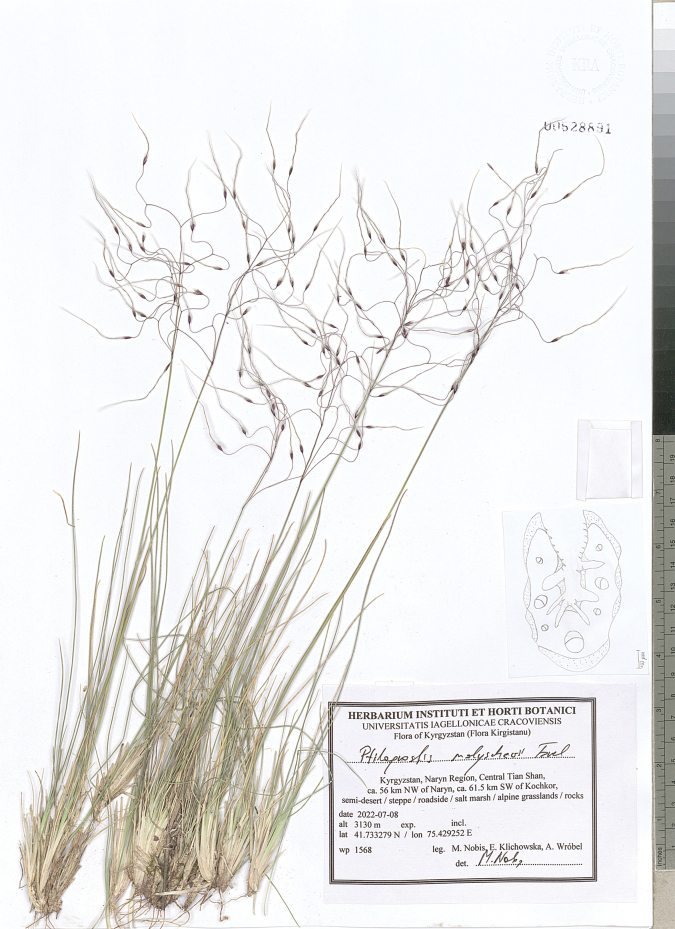
*Ptilagrostismalyschevii*, general habit.

Figs 6e, 14; additional figures in Tzvelev et al. (1974: fig. 1); Qin et al. (2004: fig. 118); https://www.gbif.org/species/4149793, http://www.efloras.org/object_page.aspx?object_id=95534&flora_id=2.

##### Distribution.

Mountain areas of north-central Central Asia, in Kyrgyzstan, Tajikistan, Kazakhstan, Russia, and China (Tzvelev 1974, 1976; Tzvelev and Probatova 2019).

##### Habitat.

Alpine mats, gravels and stony slopes, at (2600–)2900–4500(–5000) m elev.

##### Selected studied specimens of *P.malyschevii*.

China • Jilin, Highland Bai Shan; elev. 2600 m; 27 Jul 1963 (KRA528840, KRA528837, IFP15852001a0021, IFP15852001a0004) • Sichuan, grasslands between Labrang and Yellow River near camp, Wanrgon valley; among willow bushes; elev. 12200 ft.; 29 Jul 1926; *J.F. Rock 14530* (E00690600, NY). – Kazakhstan • Semirechensk, obl. Dzhar­kentsk. u. Gory Ketmen-tau. Yuzhnyy sklon, r. Kessyk ak-tas; zona alpíyskaya, syrovatyye luga; vys. 1400 sazh; 14 Aug 1917; *R. Abolino 5345* (TASH015592) • Semirechensk, obl. Dzharkentsk, u. Gory Ketmen-tau Pereval Ak-tas; zona alpíyskaya, kamenistyy lug; vys. 1500 sazh; 13 Aug 1917; *R. Abolino 3766* (TASH015593). – Kyrgyzstan • Issyk-Kul Region, Central Tian Shan, ca. 133.5 km ESE of Balykchy, ca. 25 km S of Barskoon; alpine grasslands; elev. 2790 m; 41.924582°N, 77.641257°E; 5 Jul 2022; *M. Nobis, E. Klichowska, A. Wróbel 1547* (KRA628874) • Issyk-kul Region, ca. 96 km to the W from the Is­syk-kul Lake and ca. 51 to the SE from Przewalsk, near the road A364; over the mountain pass; elev. 3491 m; 42°21'58.54"N, 79°1'27.63"E; 2 Aug 2016; *M. Nobis, A. Nobis 838* (KRA487167) • Naryn Region, Central Tian Shan, ca. 87.5 km SW of Naryn, ca. 58.8 km SW of At-Bashy; alpine grasslands; elev. 3112 m; 10 Jul 2022; 40.822641°N, 75.289191°E; *M. Nobis, E. Klichowska, A. Wróbel 1580* (KRA628893, KRA628894, KRA628895) • Central Tien Shan, ca. 76.5 km E of Kyzyl Suu, ca. 192 km SE of Almaty; alpine meadow; elev. 2810 m; 42°25'35.14"N, 78°56'53.41"E; 09 Jul 2015; M. Nobis, *A. Nowak 632* (KRA476208) • Central Tien Shan, ca. 52 km E of Przewalsk, ca. 197 km SE of Almaty; alpine grassland; elev. 3428 m; 42°25'37.73"N, 79°1'41.85"E; 09 Jul 2015; *M. Nobis, A. Nowak 636* (KRA481613) • Central Tian-Shan, ca. 18 km NE of Songköl, ca. 40.5 km SW of Kochkor; steppe; elev. 2892 m; 41°56'55.56"N, 75°25'46.59"E; 31 Jul 2016; leg. *M. Nobis, A. Nobis 829* (KRA522756). Addition­al specimens studied are listed in Suppl. material 1.

##### Note.

During the revision of herbarium materials in Shenyang (IFP), we found specimens collected in NE China that are morphologically intermediate between *P.malyschevii* and *P.alpina*. These specimens have short ligules, scabrous panicle branches and fewer flowers in the panicle that are typical for *P.alpina*, while other features such as long awns, large lemmas and glumes are characteristic for *P.malyschevii*. It is possible that *P.alpina* is also present in the area and hybridisation between the two species occurs there. Further studies are required to verify this hypothesis.

##### Selected specimens characterised by intermediate characters between *P.malyschevii* and *P.alpina* studied.

China • Antu County, Changbai Moun­tain reserve; 23 Jul 1986 (KRA528839, IFP15852001a0026) • 29 Jul 1975 (KRA528838, IFP15852001a0024).

#### ﻿9. Ptilagrostis mongholica

Taxon classificationPlantaePoalesPoaceae

(Turcz. ex Trin.) Griseb., Flora Rossica 4(13): 447. 1852.

F340102E-532A-5FB2-94A7-64428F2954C6

Suppl. material 2: fig. S11

Stipa mongholica Turcz. ex Trin., Mémoires de l’Académie Impériale des Sciences de Saint-Pétersbourg. Sixième Série. Sciences Mathématiques, Physiques et Naturelles. Seconde Partie: Sciences Naturelles 4,2(1): 42. 1836. **Basionym.**
≡ Achnatherummongholicum (Turcz. ex Trin.) Ohwi, Journal of Japanese Botany 17(7): 403. 1941.
≡ Lasiagrostismongholica (Turcz. ex Trin.) Trin. & Rupr., Species Graminum Stipaceorum 87. 1842.
≡ Oryzopsismongolica (Turcz. ex Trin.) Beal, Botanical Gazette 15(5): 111. 1890.

##### Type.

In pratis humidis torrentem Dschiginai in Okam influentem [E Sayan], 1830, *Turcz[aninov*] s.n. (lectotype: LE01009420!, designated [as holotype] by Tzvelev 1976: 556; syntypes: H, K, KFTA, LE (12 sheets!), LECB, US, W).

#### ﻿9a. Ptilagrostis mongholica subsp. mongholica

Taxon classificationPlantaePoalesPoaceae

AE8DDAFF-1C81-5DD4-8160-A9EC791CECD9

= Stipaczekanovskii Petrov, Flora Iakutiae 1: 136, f. 42. 1930; ≡ Ptilagrostisczekanowskii (Petrov) Sipliv., Spisok Rastenij Gerbarija Flory SSSR 18: 60. 1970. Type: Sibiria orient. ad fl. Olenek, inter Majgada superiorem et ostium fl. Alakit, 11 Jul 1874 [fr.], *A. Czekanowski et F. Muller* s.n. (lectotype, **designated here** , LE 01009404!; isolectotypes: LE 01009405 and 01009406);
= Stipamongholicavar.minutiflora V.S. Titov ex Roshev., Flora Aziatskoi Rossii 1(12): 131–132. 1916; ≡ Ptilagrostismongholicasubsp.minutiflora (V.S. Titov ex Roshev.) Tzvelev, Novosti Sistematiki Vysshchikh Rastenii 11: 7. 1974; ≡ Ptilagrostisminutiflora (V.S. Titov ex Roshev.) Czerep., Sosudistye Rasteniia SSSR 379. 1981]. Type: Russia: [Siberia], Enis. gub. Minus., y. Abakanskaya inorodnaya uprava, dol. rr. Ulenya i Karo, bolotnistyi lug, 1–3 Aug 1909, *V. Titov* s.n. (lectotype, **designated here**, LE01009407!, isolecto­type: LE 01009408!, TK (2 sheets!, including one with original, hand-written label; syntypes LE01009409! and LE01009410!

##### Description.

***Perennial plants***, densely tufted, with a few culms and numerous vegetative shoots; culms (10–)20–50(–60) cm tall. ***Leaves of vegetative shoots***: sheaths glabrous; ***ligules*** lanceolate; ***blades*** scabrous, convolute, green, pale green to greyish, 0.3–0.5 mm in diameter, with 3 vascular bundles. ***Cauline leaves***: sheaths glabrous or minutely scabrous; ***ligules*** on the lower sheaths lanceolate. ***Panicle*** open, 12–17 cm long and 5–9 cm wide; ***branches*** glabrous. ***Glumes*** subequal, purple, (4.5–)5.0–7.0(–7.8) mm long, lanceolate. ***Floret*** (lemma + callus) (3.5–)4.0–5.5(–6.0) mm long. ***Callus*** 0.3–0.7 mm long, densely pilose; callus base obtuse. ***Lemma*** coriaceous, pale-green, purplish or brownish, covered from the bottom up to 1/3 of its length, by dense ascending to appressed hairs, hairless in the mid-length and with hairs at apex; ***lemma apex*** with two lobes. ***Awn*** (13–)15–26(–33) mm long, unigeniculate; ***the lower segment of the awn* (*column*)** 5.0–13.0(–15.0) mm long, twisted, with 1.0–2.0 mm long hairs; ***terminal segment of the awn* (*seta*)** straight, 8.0–16.0(–22.0) mm long, with 1.2–1.6 mm long hairs, gradually decreasing in length towards the apex. ***Anthers*** 1.8–3 mm long, glabrous at the apex.

##### Phenology.

Flowering from July to September.

##### Figures.

**Figure 15.**
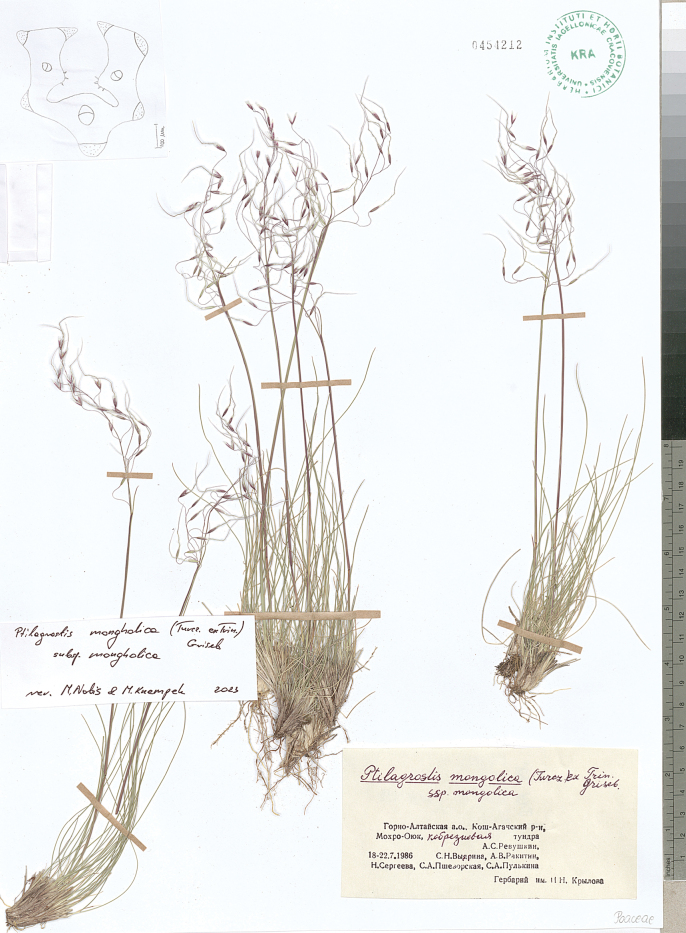
Ptilagrostismongholicasubsp.mongholica, general habit.

Figs 5m–o, 6p, 15; additional figures in Wu et al. (2007: fig. 280); https://www.gbif.org/species/2703369, http://www.efloras.org/object_page.aspx?object_id=95537&flora_id=2.

##### Distribution.

Alpine areas of north-central Central Asia, in Kazakhstan, Russia, China, and an island locality in Afghanistan (Tzvelev 1974, 1976; Wu and Phillips 2006; Tzvelev and Probatova 2019).

##### Habitat.

Ptilagrostismongholicasubsp.mongholica has wide ecological amplitude of habitat preferences. It grows on alpine mats, steppes, bogs, wet and dry meadows, rocky grasslands, at 2000–4800 m elev.

##### Note.

Within the taxon, a variety characterised by the presence of smaller glumes, smaller florets, and a greater number of flowers in a panicle (20–30) was distinguished by Titov ex Roshevitz (1916) and named S.mongholicavar.minutiflora. Later, it was raised to the rank of subspecies (Tzvelev 1974) and then to the rank of species (Czerepanov 1981; Tzvelev and Probatova 2019). This taxon was treated as endemic to Khakasia, however later there were confirmed collections also from Altai in Russia and Kazakhstan (Tzvelev and Probatova 2019). Bearing in mind that the morphological variability of *P.minutiflora* is located entirely within the smaller variability range of *P.mongholica* (number of flowers 15–28 vs. 8–22; glumes length 4.5–5 vs. 4.6–7.8 mm; floret length 3.3–4.0 vs. 3.8–6.0; awn length 13–20 vs. 15–33 mm, respectively), the independence of this taxon needs molecular confirmation (and comparison with representatives of *P.mongholicas. str.* at the population level). Based on our biometrical studies on the representatives of both taxa (including typical specimens), we consider S.mongholicavar.minutiflora to be conspecific with the nominal species. The first lectotypification of Stipamongholicavar.minutiflora was made by Tzvelev (1976: 556), however, there was no precise designation of the lectotype in the publication. On both sheets with specimens of S.mongholicavar.minutiflora at LE, there are Tzvelev’s labels stating ‘Ptilagrostisminutiflora (Titov ex Roshev.) Czer. 1981, Sosud. Rast SSSR: 379, Lectotypus!, V.1991, N. Tzvelev’.

##### Selected studied specimens of P.mongholicasubsp.mongholica.

Afghanistan • prov. Badakhshan, Wakhan, Darya-e Birgula-e Jelga Chelab Tal, Nw des Kol-e Chaqmaqtin; elev. 4200–4400 m; 37°15'N, 74°06'E; 20 Jul 1971; *O. Anders 7578* (MSB-186166). – China • Hebei, Chili, Hsiao-wu-tai-shan, Tien-lin-ssü; in prato alpino; elev. 2800 m; 22 Jul 1921; *H. Smith 1236* (V-038624) • Sichuan, reg. bor.: Dongrergo (Hsioeh-pau-ting); in silva mixta; elev. 4000 m; 20 Jun 1922; *H. Smith 3797* (V-040980). – Kazakhstan • Gorno-Altayskaya a. o., khr. Terektins­kiy, verkh. r. Karakol, r. Arykhem; lishaynikovaya tundra; 12 Jul 1983; *A. S. & T. S. Revushkiny, S. N. Vydrina, V. F. Balashova, N. I. Gordeyeva* s.n. (KRA455209). – Mongolia • Ajmak Bajanchongor, Somon Galuut, Changaj Mountains, Sant Val­ley (side from Cagan-Turutuingol); mountain steppe at the bottom of the valley; elev. 2600 m; 16 Jul 1974; *A. Pacyna* s.n. (KRA101114) • Ajmak Bajanchongor, Somon Galuut, Changai mountains, Olon-Nur valley; alpine grassland on the wa­tershed above the valley; elev. 2640 m; 6 Jul 1974; *A. Pacyna* s.n. (KRA101112) • Mungun-Moritu, Centr somona: Khentej, Dund-Bajdakagiju-Gol catchment; in NNEE part; valley depression, wet meadow; elev. 1650 m; 25 Jul 1978; F. Święs s.n. (KRA1011093). – Russia • Gorno-Altayskaya a.o., Kosh-Agachskiy r-n, Mokhro-Oyuk; kobrezyvaya tundra; 18–22 Jul 1986; *A. S. Revushkin, S. N. Vydring, A. V. Rakitin, N. Sergeyeva, S. A. Pshevorskaya, S. A. Pul’kina* s.n. (KRA451213) • Gor­no-Altaisk Autonomous Oblast, wet sedge-willow meadow between Tenga and Yabogan Pass; elev. 1100 m; 30 Aug 1978; *T.S. Elias, W. Weber, C.S. Tomb 4828* (NY) • Burjatia, distr. Bargusin, jugum Jushno-Mujsky ad fontes fl. Bargusin, in glareosis ripa sinistra fluminis Bargusin prope lac. Balan-Tomur; 7 Aug 1964; *V. Siplivinsky* s.n. (NY) • Altai, prope pug. Eschtu-kol; 27 Jul 1924; *B. Schischkin* s.n. (NY). Additional specimens studied are listed in Suppl. material 1.

#### ﻿9b. Ptilagrostis mongholica subsp. porteri

Taxon classificationPlantaePoalesPoaceae

(Rydb.) Barkworth, Systematic Botany 8(4): 417. 1983.

062B3931-1AB0-5C28-907C-6071BB986400

Stipa porteri Rydb., Bulletin of the Torrey Botanical Club 32(11): 599. 1905. **Basionym.**
≡ Ptilagrostisporteri (Rydb.) W.A. Weber, University of Colorado Studies: Series in Biology 23: 2. 1966.

##### Type.

USA: Rocky Mountains, *Hall & Harbour 648* [error for 646] (lectotype designated by Barkworth 1983: 714 in PH; isolectotypes: GH, MO-3055595, MO-305594, MO-5472475, MO-3055593, NY-431562, US-992164, US-992165, US-907470).

##### Description.

***Perennial plants***, densely tufted, with a few culms and numerous vegetative shoots; culms 20–50 cm tall. ***Leaves of vegetative shoots***: sheaths glabrous; ***ligules*** lanceolate; ***blades*** scabrous, convolute, green, pale green to greyish, 0.3–0.5 mm in diameter, with 3 vascular bundles. ***Cauline leaves***: sheaths glabrous or minutely scabrous; ***ligules*** on the lower sheaths lanceolate. ***Panicle*** open, rarely loosely contracted, 5–12 cm long and 2–6 cm wide; ***branches*** glabrous. ***Glumes*** subequal, purple, 4.5–6.0 mm long, lanceolate. ***Floret*** (lemma + callus) 2.5–4.0 mm long. ***Callus*** 0.2–0.5 mm long, densely pilose; callus base obtuse. ***Lemma*** coriaceous, pale-green, purplish or brownish, covered from the bottom up to 1/3 of its length, by dense ascending to appressed hairs, hairless in the mid-length and with hairs at apex; ***lemma apex*** with two lobes. ***Awn*** 10–23 mm long, unigeniculate; ***the lower segment of the awn* (*column*)** 4.0–6.0 mm long, twisted, with 1.0–1.7 mm long hairs; ***terminal segment of the awn* (*seta*)** straight, 8.0–12.0 mm long, with 1.2–1.6 mm long hairs, gradually decreasing in length towards the apex. ***Anthers*** 1.5–3.0 mm long, glabrous at the apex.

##### Phenology.

Flowering from July to August.

##### Figures.

**Figure 16.**
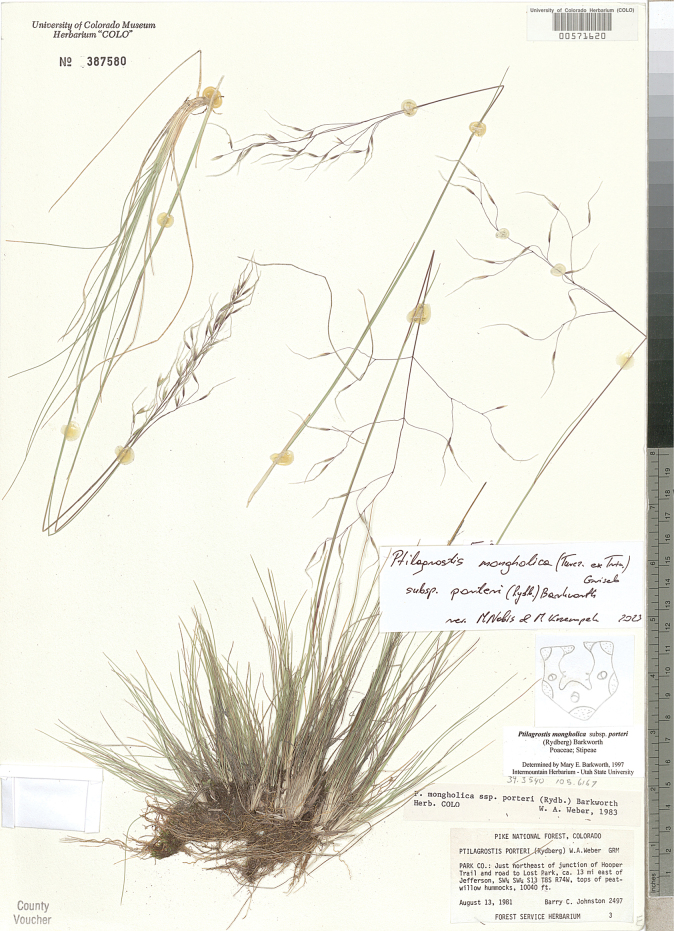
Ptilagrostismongholicasubsp.porteri, general habit.

Figs 5p–s, 16; additional figures in Barkworth (1983), Barkworth (2007: 144); Johnston (2006: 18–19); https://www.gbif.org/species/2703367.

##### Distribution.

North America: Colorado, New Mexico (Barkworth 1983; Soreng 2003; Johnston 2006).

##### Habitat.

Alpine habitats, in poorly-drained wetlands and wet meadows, at 2700–3650 m elev.

##### Note.

The taxon is the most similar to *P.mongholica*, however, it differs in having smaller glumes, lemmas and awns as well as by the general distribution range. Based on morphology (and also habitat preferences), P.mongholicasubsp.porteri is the taxon most similar to S.mongholicavar.minutiflora that was described 11 years later by Roshevitz (1916). We treat it here as conspecific with *P.mongholica*. However, further studies with the involvement of molecular methods are needed to reconstruct the phylogenetic relationships within this complex of taxa. Weber (2003) stressed that P.mongholicasubsp.porteri differs from the nominal subspecies in habitat requirements, and the first taxon is restricted to montane fens and willow carrs, while subsp. mongholica is a taxon of rocky sites, mountain grasslands, and alpine meadows. Based on our observations and analysis of the habitat descriptions on herbarium labels, P.mongholicasubsp.mongholica can grow in both dry and wet habitats (see above); thus, the habitat parameter does not seem to be a good enough character for the differentiation of these two taxa. Johnston (2006) mentioned that some populations of P.mongholicasubsp.porteri have open panicles, while the other is contracted. Because individuals with open and contracted panicles can be found, sometimes even in the same populations of P.mongholicasubsp.porteri, their taxonomic status should be a subject of further research.

##### Selected studied specimens of P.mongholicasubsp.porteri.

United States • Park Co., South Park, 4 miles W of Jefferson, near Fremont’s Knoll (see Pike Nat. Forest map); elev. 10,000 ft.; on peat hummocks in rich calcareous fen; 27 Jul 1989; *D.J. Cooper 1682* (COLO00769711) • Park Co., Long Gulch, north of the road to Lost Park, 11.3 mi E of Jefferson; SW¼ SW¼ S13 T8S R74W; elev. 10020–10030 ft.; 4 Oct 1982; *B.C. Johnston, L. Hendzel 2655* (COLO00571570, COLO00571588) • Park Co., South Park. Hummocky area on edge of rich fen, Forest Service land ¼ mi NW of Silverheels Ranch house; 6 Jul 1990; *D.J. Coo­per 1788* (COLO00571455) • Park Co., Lost Park, bottom just S of Lost Park Campground,;NW¼ NW¼ S12 T9S R73W; elev. 9840 ft.; 4 Oct 1982; *B.C. John­ston, L. Hendzel 2658* (COLO571471) • Park Co., Geneva Park, Sec. 13; T.6S., R. 75W.; elev. 9,700 ft.; on hummocks in meadow; 26 Jul 1966; *R. Gierisch, W.C. Hickey 3102* (COLO00571513) • Park Co., South Park, High Creek Fen, 10 mi. S of Fairplay; elev. 9300 ft.; on hummocks in moist part of calcareous fen, with *Salixcandida*, *S.brachycarpa*, *Pentaphylloidesfloribunda*, *Carexscirpoidea*, *Par­nassia parviflora*; 12 Aug 1996; *N. Lederer, W. Jennings, W. Marotti, P. Murphy 96-HC-1* (COLO00571497) • Park Co., South Park, on peat hummocks in rich fen, Albert Wahl Ranch at base of Kenosha Pass, 6 Sep 1990, *D.J. Cooper 1942* (COLO00571604). Park Co., just northeast of junction of Hooper Trail and road to Lost Park, ca. 13 mi east of Jefferson; SW¼ SW¼ S13 T8S R74W; tops of peat- willow hummocks; elev. 10040 ft.; 13 Aug 1981; *B.C. Johnston 2497* (COLO00571620) • Park Co., 11.3 mi. SE of Jefferson on road to Lost Park; on peaty hummocks of willow streamside, with *Salix*, *Betulaglandulosa* and *Potentillafruticosa*; 25 Sep 1966; *W.A. Weber 12984* (COLO00571521).

#### ﻿10. Ptilagrostis tibetica

Taxon classificationPlantaePoalesPoaceae

(Mez) Tzvelev, Rasteniia Tsentral’noi Azii 4: 45. 1968.

D997BFE8-CF20-5CA5-B92E-DC2F3A0E53A6

Suppl. material 2: fig. S12

Stipa tibetica Mez, Repertorium Specierum Novarum Regni Vegetabilis 17(13–18): 207. 1921. **Basionym.**

##### Type.

Tibet Occ. Regio alp., Lasiag[rostis] Mongholica Trin., 14,000 ft., *Hooker fil. & Thomson* s.n. (holotype: B destroyed; lectotype K – H2012/0158/8! (Herbarium Hookerianum (1867) – specimen in the middle part of the sheet) **designated here**, isolectotype LE00009272).

##### Description.

***Perennial plants***, densely tufted, with a few culms and numerous vegetative shoots; culms 20–45(–70.0) cm tall. ***Leaves of vegetative shoots***: sheaths glabrous; ***ligules*** lanceolate; ***blades*** scabrous, filiform, convolute, green, pale green to greyish, 0.20–0.35 mm in diameter, with 3(–5) vascular bundles. ***Cauline leaves***: sheaths glabrous or minutely scabrous; ***ligules*** on the lower sheaths lanceolate. ***Panicle*** open, 8–16 cm long and 5–10 cm wide; ***branches*** glabrous. ***Glumes*** subequal, purple, (4.5–)5.0–6.5 mm long, lanceolate. ***Floret*** (lemma + callus) 3.6–5.0 mm long. ***Callus*** 0.3–0.5 mm long, densely pilose; callus base obtuse. ***Lemma*** coriaceous, pale-green, purplish or brownish, covered from the bottom up to 1/3 of its length, by dense ascending to appressed hairs, hairless in the mid-length and with hairs at apex; ***lemma apex*** with two lobes. ***Awn*** 10–16 mm long, unigeniculate; ***the lower segment of the awn* (*column*)** 5–7 mm long, twisted, with 1.2–2.0 mm long hairs; ***terminal segment of the awn* (*seta*)** straight, 7–10 mm long, with 1.2–1.6 mm long hairs, gradually decreasing in length towards the apex. ***Anthers*** 2–3 mm long, glabrous at the apex.

##### Phenology.

Flowering from August to September.

##### Figures.

Figs 4a–c, 6s, 17.

**Figure 17.**
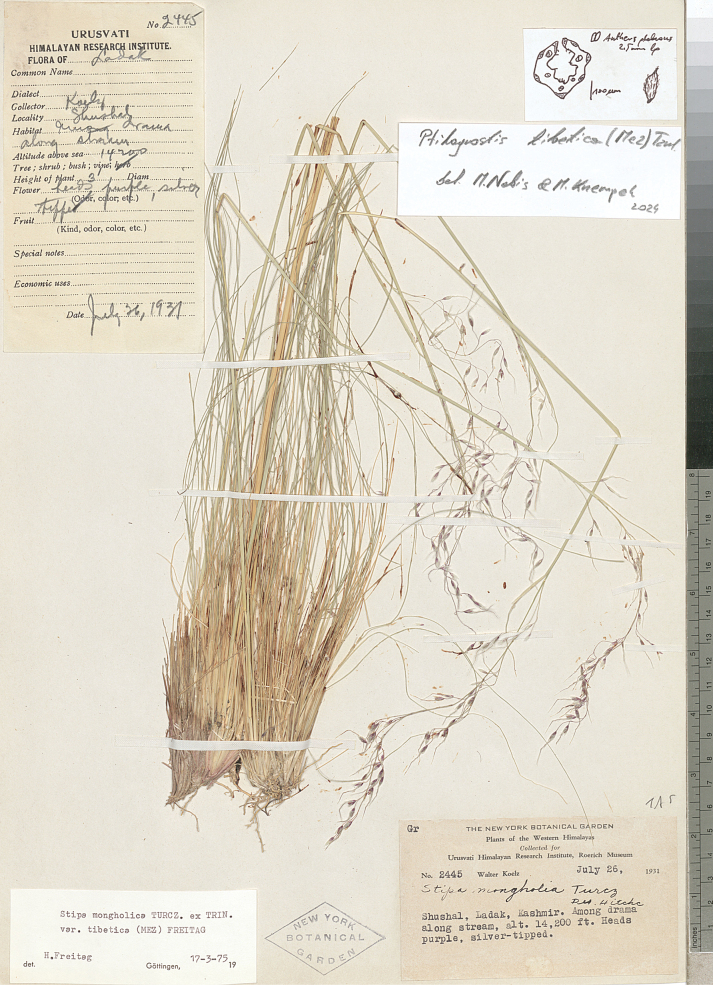
*Ptilagrostistibetica*, general habit.

##### Distribution.

Mountain areas of south and south-western Central Asia, in India, Nepal and China (Zhang and Chen 2024).

##### Habitat.

Alpine meadows and cryophilous steppes, at 4200–4800 m elev.

##### Selected studied specimens of *P.tibetica*.

India • Tibet Occ. Subchilum Kumaon; elev. 14500 ft.; *R. Strachey, J.E. Winterbottom* s.n. (H2012/0158/8) • NW India, Jammu and Kashmir State, Ladakh, Indus Vy: Zhung (Leh), Sha­grot to confluence of Purang and Kyammar Lungpa; elev. 4370 m; 33°36,4'N, 77°45,8'E; 4 Sep 2001; *L. Klimeš 1533* (KRA479095) • Ladakh, Rupshu, Tso Mori­ri, Zerlung Marlung; elev. 4540 m; 32°53'N, 78°16,5'E; 25 Aug 1999; *L. Klimeš 658* (KRA479075, KRA479096) • Shushal, Ladak; among drama along stream; elev. 14,200 ft.; 26 Jul 1931; *W. Koelz 2445* (NY) • Tsakzhun Tso, Ladak; along stream; elev. 15,000 ft.; 21 Jul 1931; *W. Koelz 2401* (NY). – Nepal • Dolpo, Maha­jung Khola, 6 miles E of Tingkyu; elev. 4800 m; 4 Aug 1973; *Grayhilson, Phillips 489* (H2012/0158/9).

##### Note.

Since *P.tibetica* grows together or in neighbouring localities with *P.dichotoma*, gene flow among these two taxa may sometimes occur. The result of such hybridisation can be specimens collected by Dickoré from Tibet, which are characterised by lemmas covered up to 2/3 by long hairs and long prickles above.

##### Studied specimens of potential hybrids *P.tibetica* × *P.dichotoma*.

China • S Tibet, Xizang: Tibetan Himalaya N of Bhutan, Kuru Chu, Hill SW of Lhozak Vy. junction; subalp, moist meadow; 28°18'N, 90°51’ E; elev. 4200 m; 22 Jul 1994; *B. Dickoré 9758* (MSB-152874) • Tsangpo tributary, Nangxian - Mainling, Lilung Chu Eastern branch (High Camp); alp. moist turf spots, screes and boulder fields, gneiss; 29°0'N, 93°59'E; elev. 4820 m; 11 Aug 1994; *B. Dickoré 10819* (MSB-152848).

#### ﻿ Ptilagrostis sect. Barkworthia

Taxon classificationPlantaePoalesPoaceae

M. Nobis, A. Nobis & A. Nowak

063489F2-F944-5E1A-8E6D-36CB928C3CA2

##### Type.

*P.yadongensis* Keng & Tang

##### Description.

Species belonging to this section are characterised by clearly unequal lemmas and paleas (lemma 0.7–2.0 mm longer than palea), awns with hair on the upper segment < 1 mm long (usually 0.2–1.0 mm long) and 2–3 times shorter than on column, and the glumes unequal.

#### ﻿11. Ptilagrostis yadongensis

Taxon classificationPlantaePoalesPoaceae

Keng & Tang, Journal of Southwest Agricultural University 4: 44. 1985.

58A76B52-5F91-5D6C-9073-720DCF63F22C

≡ Ptilagrostismacrospicula Cai, Acta Botanica Boreali-Occidentalia Sinica 23(11): 2018. 2003. superfl. name.
= Stipamilleri Noltie, Edinburgh Journal of Botany 56(2): 288. 1999; ≡ Ptilagrostismilleri (Noltie) M. Nobis & A. Nobis, Nordic Journal of Botany 31: 623. 2013.Type: India, Sikkim, Goichang, Lasha Chhu valley, 27°55'52″N, 88°36'17″E, 4555 m a.s.l., 19 Jul 1996, *EENS 349* (holotype: E!, isotype: BSHC).

##### Type:

China, Xizang: Yadong, 14 Sept. 1974, *Qinghai-Xizang Exped. 74–2496* (lapsus calami as 74–2469; holotype: HNWP, isotype: PE).

##### Description.

***Perennial plants***, densely tufted, with a few culms and numerous vegetative shoots; culms (7–)8–15(–20) cm tall, 1–2-noded distributed in the lower part of the culm and usually hidden within the leaf-sheath. ***Leaves of vegetative shoots***: sheaths glabrous; ***ligules*** lanceolate, on the external sheaths (1.0–)1.3–1.5(–1.6) mm long, whereas on the internal sheaths, (1.3–)1.6–2.0(–4.0) mm long; ***blades*** filiform, convolute, green to pale green or greyish, (5.7–)7.0–9.0 cm long, 0.3–0.4(–0.5) mm in diameter, with 3–5 vascular bundles, adaxial (upper) surface covered by 0.05–0.1 mm long hairs, abaxial surface scabrous or less frequently (some leaves) glabrous. ***Cauline leaves***: sheaths glabrous; ***ligules*** on the lower sheaths lanceolate (1.2–)1.4–1.7(–1.8) mm long, on the middle and upper sheaths (1.5–)1.7–2.6(–3.0) and (1.7–)2.0–3.7(–4.0) respectively; ***blades*** convolute, green, pale green or greyish, adaxial (upper) surface covered with 0.05–0.1 mm long hairs, abaxial (lower) surface scabrous. ***Panicle*** contracted, (3.5–)3.9–4.9(–5.3) cm long, at base enclosed by the sheath of the uppermost leaf; branches ascending, scabrous or almost so, single or paired, lower branch (1.9–)2.1–2.9(–3.5) cm long. ***Glumes*** unequal, the lower (1.5–)1.8–2.5(–2.7) mm longer than the upper, brownish or purplish, lower glume (10.0–)11.0–11.5(–12.3) mm long, upper glume (7.5–)8.5–10.0(–11.0) mm long, lanceolate. ***Floret*** (=anthecium, =lemma + callus) 5.0–6.3(–6.6) mm long. ***Callus*** (0.4–)0.5–0.6 mm long, densely pilose, on ventral part with hairs 0.4–0.5 mm long, on dorsal with 0.4 mm long hairs; callus base 0.4–0.5 mm long and 0.3–0.4 mm in diameter, obtuse. ***Lemma*** coriaceous, pale-green, purplish or brownish, covered from the bottom up to 1/3 of its length, by dense ascending to appressed hairs 0.4–0.5 mm long, hairless in the mid-length and with hairs at apex; ***lemma apex*** with unequal hairs (0.4–)0.6–0.8 mm long and with two apical lobes (0.3–)0.4–0.5(–0.6) mm long. ***Palea*** in (0.3–)0.7–1.3(–1.5) mm shorter than lemma in length. ***Awn*** (13–)15–17(–18) mm long, unigeniculate; ***the lower segment of the awn* (*column*)** (5–)6–7(–8) mm long, twisted, with (1.2–)1.4–1.7(–1.8) mm long hairs; ***terminal segment of the awn* (*seta*)** straight, 8–10 mm long with hairs shorter than those on columns, (0.6–)0.7–0.9 mm long, gradually decreasing in length towards the apex. ***Anthers*** ca. 0.7–1.4 mm long, glabrous at the apex.

##### Phenology.

Flowering from July to September.

##### Figures.

**Figure 18.**
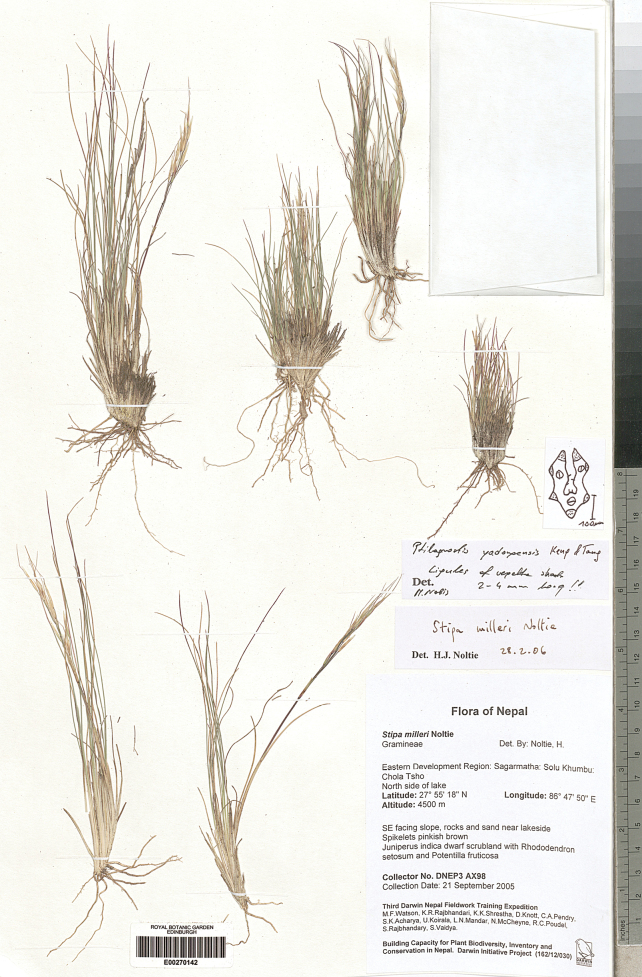
*Ptilagrostisyadongensis*, general habit.

Figs 4p–s, 6m, n, 18; additional figures in Noltie (1999: 286) and Cai (2003).

##### Distribution.

Bhutan, China: Xizang, Nepal (Noltie 1999; Wu and Phillips 2006; Nobis and Nobis 2013; Nobis et al. 2015, 2019b; Zhang et al. 2016b).

##### Habitat.

Alpine grasslands, open moraine screes, rocks and sands near lakesides, at 3500–4900 m elev.

##### Selected studied specimens of *P.yadongensis*.

Bhutan • Thimphu (Upper Mo Chu), Lemcheng; herbaceous vegetation regenerating after fire on S-fac­ing slop; elev. 4550 m; 27°58'N, 89°30'E; 16 Jul 2000; *G. & S. Miehe 00-211-11* (E00180489). – China • Xizang: S Tibet, Tibetan Himalaya, Everest E, Kama Chu, Pethang Ringmo Up, Kangchung Gl; 27°59'N, 87°6'E; elev. 4770 m; alp. dry *Ko­bresia pygmaea-Stipa* turf, cushions and open moraine scree, gneiss; 13 Oct 1989; *B. Dickoré 6361* (KRA528812, MSB-152846). – Nepal • Pandang Keipo (Up­per Langtang); elev. 4600 m; *Kobresianepalensis* mat with Gramineae, S-facing slope, grazed seasonally; 30 Sep 1986; *G & S. Miehe 13090* (KRA528815) • East­ern Development Region, Sagarmatha, Solu Khumbu, Chola Tsho, north side of lake; 27°55'18"N, 86°47'50"E; elev. 4500 m; SE facing slope, rocks and sand near lakeside, *Juniperusindica* dwarf scrubland with *Rhododendronsetosum* and *Potentillaruticose*; 21 Sep 2005; *M.F. Watson, K.R. Rajbhandari, K.K. Shres­tha, D. Knott, C.A. Pendry, S.K. Acharya, U. Koirala, L.N. Mandar, N. McCheyne, R.C. Poudel, S. Rajbhandary, S. Vaidya DNEP3 AX98* (E00270142). Additional specimens studied are listed in Suppl. material 1.

#### ﻿12. Ptilagrostis bhutanica

Taxon classificationPlantaePoalesPoaceae

(Noltie) M. Nobis, PhytoKeys 128: 109. 2019.

26FB5A6E-92DD-56CC-A0D2-96AD0BAB0DE9

Stipa bhutanica Noltie, Edinburgh Journal of Botany 56(2): 289. 1999. **Basionym.**

##### Type:

Bhutan. Ha: W side of Chelai La, 29 Sept. 1998, *H.J. Noltie, N. Pradhan, Sherub & T. Wangdi 349* (holotype: E!, isotype: THIM).

##### Description.

***Perennial plants***, densely tufted, with a few culms and numerous vegetative shoots; culms (6–)8–41(–54) cm tall, 2-noded distributed in the below the middle of the culm length, the upper one usually visible the lower hidden within the leaf-sheath. ***Leaves of vegetative shoots***: sheaths glabrous or less frequently scabrous; ***ligules*** lanceolate, on the external sheaths (0.9–)1.2–1.8(–2.3) mm long, whereas on the internal sheaths, (1.3–)1.6–2.5(–3.5) mm long; ***blades*** filiform, convolute, green, pale green to greyish, (6.1–)6.5–16.0(–25.5) cm long, 0.4–0.6(–0.7) mm in diameter, with (5–)7 vascular bundles, adaxial (upper) surface covered by 0.05–0.1 mm long hairs, abaxial (lower) surface scabrous (some leaves can be glabrous). ***Cauline leaves***: sheaths glabrous; ***ligules*** on the lower sheaths lanceolate, (1.0–)1.5–2.0 mm long, on the middle and upper sheaths (1.5–)1.8–2.5(–3.5) and (2.0–)2.4–3.0(–3.7) respectively; ***blades*** convolute, green, pale green or greyish, adaxial surface covered with short hairs, abaxial surface scabrous. ***Panicle*** contracted, (3.6–)5.0–9.5(–13.0) cm long; ***branches*** ascending, scabrous, single or paired, lower branch (1.8–)2.7–5.0(–6.0) cm long. ***Glumes*** subequal, the lower 0.2–0.5(–0.8) mm longer than the upper, dark purple, lower glume 7.2–9.4(–12.0) mm long, upper glume (7.0–)7.3–9.0(–11.5) mm long, lanceolate. ***Floret*** (lemma + callus) (5.8–)6.1–7.5(–8.4) mm long. ***Callus*** 0.4–0.6(–0.7) mm long, densely pilose, on ventral part with hairs 0.3– 0.5(–0.7) mm long, on dorsal with (0.3–)0.4–0.5 mm long hairs; callus base 0.4–0.5(–0.6) mm long and 0.3–0.4 mm in diameter, obtuse. ***Lemma*** coriaceous, pale-green, purplish or brownish, covered from the bottom up to 1/3 of its length, by dense ascending to appressed hairs 0.3–0.5(–0.7) mm long, hairless in the mid-length and with hairs at apex; ***lemma apex*** with unequal hairs (0.3–)0.5–1.2(–1.8) mm long and without apical lobes. ***Palea*** (1.0–)1.2–2.0(–2.3) mm shorter lemma in length. ***Awn*** (12.0–)13.5–15.0(–17.0) mm long, unigeniculate; ***the lower segment of the awn* (*column*)** (4.5–)5.0–6.0(–7.0) mm long, twisted, with (0.5–)0.6–0.9 mm long hairs; ***terminal segment of the awn* (*seta*)** straight, (7–)8–9(–10) mm long, with hairs shorter than those on columns, 0.3–0.5 mm long, gradually decreasing in length towards the apex. ***Anthers*** ca. 0.8–2.0 mm long, glabrous at the apex.

##### Phenology.

Flowering from July to September.

##### Figures.

**Figure 19.**
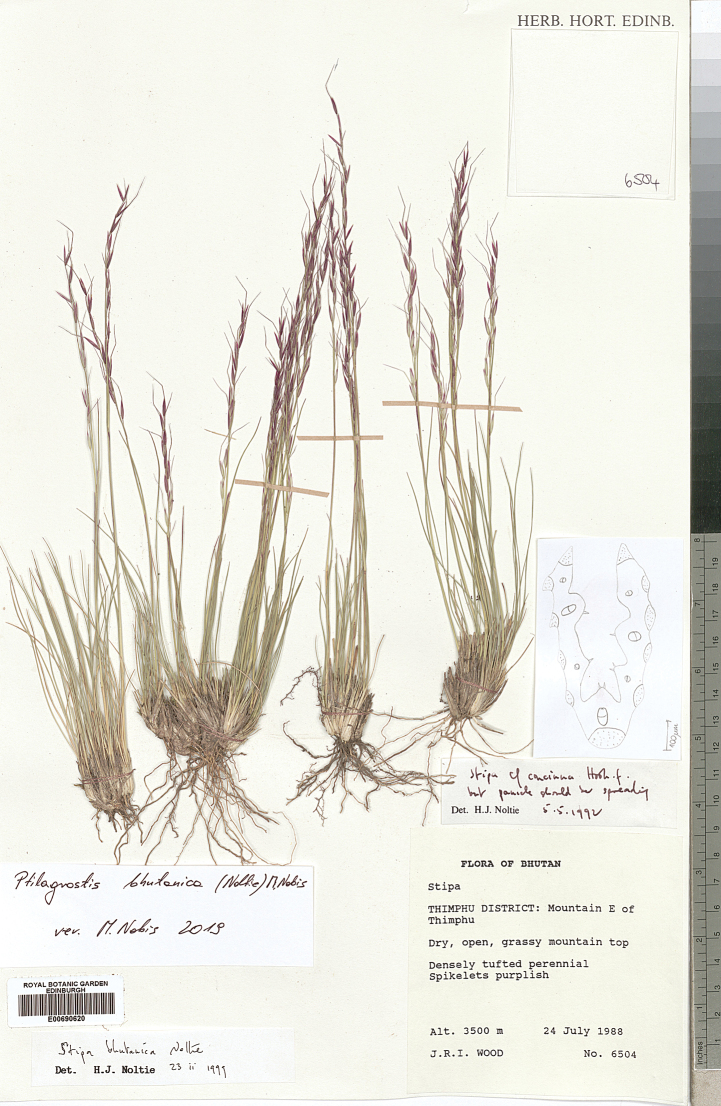
*Ptilagrostisbhutanica*, general habit.

Figs 4d–f, 6i, 19; additional figures in Noltie (1999: 286); https://www.gbif.org/species/12192155; https://powo.science.kew.org/taxon/urn:lsid:ipni.org:names:77211695-1.

##### Distribution.

Bhutan, China: Sichuan, Yunnan, Xizang (Noltie 1999; Nobis et al. 2016, 2020; Zhang et al. 2016b).

##### Habitat.

Alpine grasslands, pastures, thickets and scrubs, forests at tree-line, at 3500–4500 m elev.

##### Selected studied specimens of *P.bhutanica*.

Bhutan • Thimphu District: Mountain E of Thimphu. Dry; open, grassy mountain top; elev. 3500 m; 24 Jul 1988; *J.R.I. Wood 6504* (E00690620) • Gasa (Upper Mo Chu), Rodophu; forb-rich pasture encroached by *Rhododendronlepidotum* on SE-facing slope; elev. 4330 m; 28°2'N, 89°47'E; 16 Aug 2000; *G. & S. Miehe 00-289-31* (E00180486) • Gasa (Upper Pho Chu), Tarina Camp; *Abiesdensa* forest on S-facing lower slope; elev. 4040 m; 28°3'N, 89°57'E; 1 Sep 2001; *G. & S. Miehe 00-342-07* (E00180448). – China • Sichuan: Hanyuan County, Shuajingsi Town northeast mountain; subalpine environment; *Z.X. Tang 1486* (KRA628871) • Kangding County, Jianguan camp; subalpine meadow; *X.W. Tian 165(6)* (KRA628872, KRA628873) • Litang County, Pingdi; environment alpine; elev. 4300 m (KRA528813) • Litang County; alpine meadow, south hillside; *X.W. Tian 149* (KRA628870) • Xizang: E Tibet, Ningjing Shan, Mekong (Lancang) tributary, W of Markham (Gartog); 29°41'N, 98°30'E; elev. 4300 m; subalp.-lower alp. turf, *Rhododendron* dwarf-scrub, *Picea* forest at tree-line; 1 Jul 1994; *B. Dickoré 8628* (MSB-152870, E00132107).

#### ﻿ Ptilagrostis sect. Chenella

Taxon classificationPlantaePoalesPoaceae

M.Nobis, Krzempek & Klichowska sect. nov.

1BF8EA93-0D7E-5A04-90C2-91A791FB78AD

urn:lsid:ipni.org:names:77351832-1

##### Type.

﻿*Ptilagrostisduthiei* (Hook.f.) M.Nobis & P.D.Gudkova.

##### Description.

Species belonging to this section are characterised by having upper awn segments scabrous, culms (40–)50–100 cm tall, leaves of the vegetative shoots with 7–11 veins.

##### Etymology.

The name of the section honours the eminent botanist Professor Wen-Li Chen (Chinese Academy of Sciences, Beijing, China), for her contribution to the knowledge of grasses.

#### ﻿13. Ptilagrostis chingii

Taxon classificationPlantaePoalesPoaceae

(Hitchc.) M.Nobis & Krzempek comb. nov.

90F885FF-65AB-5B4C-9C5A-7B61258DF276

urn:lsid:ipni.org:names:77351833-1

Suppl. material 2: fig. S13

Stipa chingii Hitchc., Proceedings of the Biological Society of Washington 1930, xliii. 94. 1930. **Basionym.**
≡ Achnatherumchingii (Hitchc.) Keng, Claves Generum et Specierum Graminearum Primarum Sinicorum 213. 1957; nom. inval.
≡ Achnatherumchingii (Hitchc.) Keng, Flora Tsinlingensis 1(1): 152. 1976.

##### Type.

China, Kansu Province, vicinity of Labrang, [collected in open woods, 4000 m] up to 3 ft., elev. 3000 to 4000 m, 17–20 Aug 1923, *R.C. Ching 785* (holotype: US-1245799; isotype E00890601!).

##### Description.

***Perennial plants***, densely tufted, with a few culms and numerous vegetative shoots; culms (35–)50–70(–95) cm tall, 2–3-noded distributed usually in the middle part of the culm, exerted from the leaf-sheaths. ***Leaves of vegetative shoots***: sheaths glabrous; ***ligules*** lanceolate, on the external sheaths (0.4–)1.0–1.8(–2.5) mm long, whereas on the internal sheaths, (0.8–)1.3–2.0(–2.5) mm long; ***blades*** filiform, convolute, green to pale green, (15.4–)23.0–28.9(–35.3) cm long, (0.2–)0.3–0.4(–0.5) mm in diameter, with 3–5 vascular bundles, adaxial surface covered by 0.1–0.2 mm long hairs, abaxial surface scabrous or rarely glabrous. ***Cauline leaves***: sheaths glabrous or less frequently minutely scabrous; ***ligules*** on the lower sheaths lanceolate (1.1–)2.0–2.7(–3.3) mm long, on the middle and upper sheaths (1.8–)2.5–3.8(–4.8) and (2.3–)3.0–4.3(–5.1) respectively; ***blades*** convolute, green, pale green or greyish, adaxial surface covered with short hairs, abaxial surface scabrous or glabrous. ***Panicle*** contracted to loosely contracted (sporadically open in var. laxum), (7.5–)12.0–19.5(–25.0) cm long; ***branches*** ascending, usually scabrous, single or paired, lower branch (2.1–)4.7–7.8(–11.3) cm long. ***Glumes*** subequal, the lower 0.1–0.3(–0.6) mm longer than the upper, yellowish, brown, green or purple, lower glume (5.3–)6.4–9.0(–10.2) mm long, upper glume (5.3–)6.4–8.5(–10.2) mm long, lanceolate. ***Floret*** (lemma + callus) (5.2–)5.9–6.7(–7.2) mm long. ***Callus*** (0.4–)0.5–0.7(–0.8) mm long, densely pilose, on ventral part with hairs (0.3–)0.4–0.6(–0.7) mm long, on dorsal with (0.3–)0.4–0.6(–0.8) mm long hairs; callus base 0.4–0.5(–0.6) mm long and 0.3–0.4(–0.5) mm in diameter, obtuse. ***Lemma*** coriaceous, pale-green, purplish or brownish, covered from the bottom up to 1/3 of its length, by dense ascending to appressed hairs (0.3–)0.4–0.5(–0.6) mm long, hairless in the mid-length and with hairs at apex; ***lemma apex*** with unequal hairs (0.2–)0.3–0.4(–0.5) mm long and with two apical lobes (0.5–)0.6–0.9(–1.2) mm long. ***Palea*** equal or slightly, in 0.2–0.4 mm shorter than lemma. ***Awn*** (11–)12–14(–16) mm long, 1-geniculate; ***the lower segment of the awn* (*column*)** (4.0–)5.0–6.0(–7.0) mm long, twisted, with (0.4–)0.5–0.6(–0.7) mm long hairs; ***terminal segment of the awn* (*seta*)** straight, (6–)7–9(–10) mm long, scabrous, at base with 0.2–0.3 mm long hairs, gradually decreasing in length towards the apex. ***Anthers*** ca. 2.0–3.3 mm long, bearded (occasionally glabrous) at the apex.

##### Phenology.

Flowering from July to September.

##### Figures.

**Figure 20.**
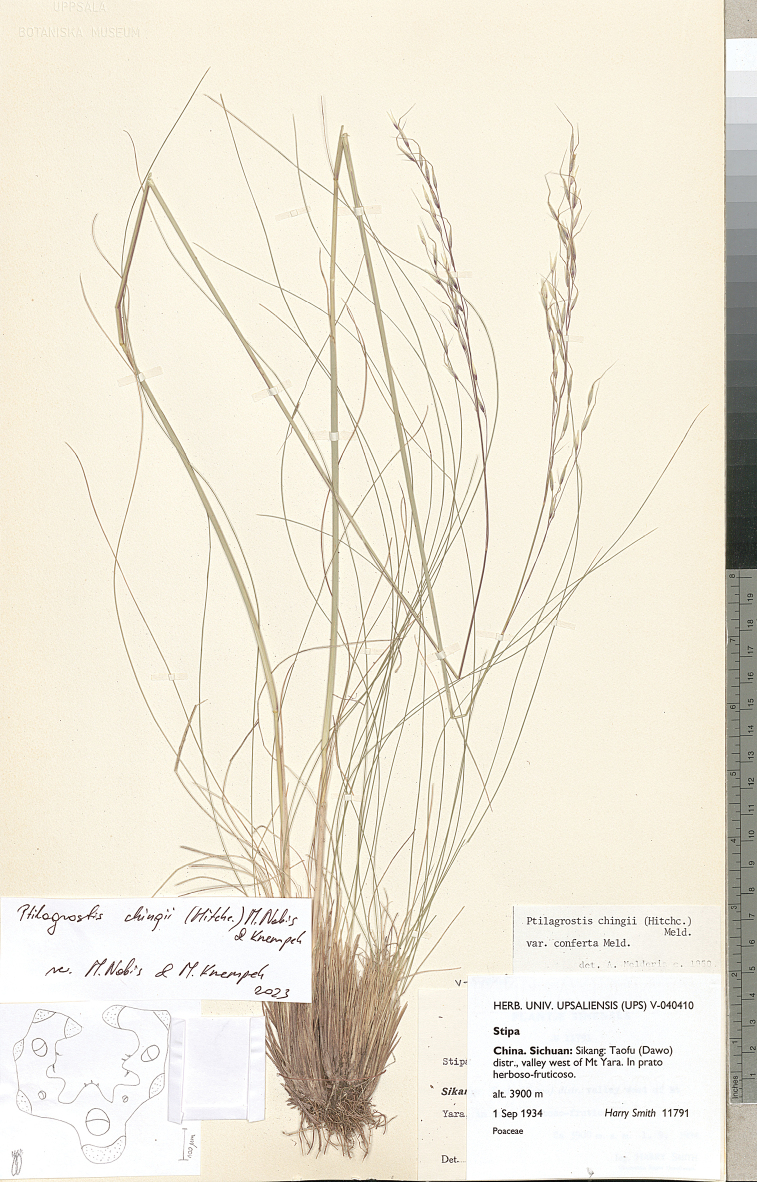
*Ptilagrostischingii*, general habit.

Figs 4g–i, 6g, h, 20; additional figures in Wu et al. (2007: fig. 286); http://www.efloras.org/object_page.aspx?object_id=94971&flora_id=2; https://www.gbif.org/tools/zoom/simple.html?src=//api.gbif.org/v1/image/cache/occurrence/1018988494/media/46df1686e91fd3599853d46b42f1d539.

##### Distribution.

Bhutan, China: Gansu, Qinghai, Shaanxi, Shanxi, Sichuan, Xizang, Yunnan, India (Wu and Phillips 2006).

##### Habitat.

Alpine grasslands, pastures, steep rocky, dry slopes, thickets, coniferous and mixed forests, at 2000–4100 m elev.

##### Selected studied specimens of *P.chingii*.

Bhutan • Gasa (Upper Mo Chu), Thangkaphu Chu/Tsharijathang junction; open *Abies-Betula* forest on NE-fac­ing slop, Takin pasture and rest places; elev. 4070 m; 27°59'N, 89°32'E; 23 Jul 2000; *G.& S.Miehe 00-237-03* (E00180485). – China • Kansu: vicinity of Labrang; elev. 3000 to 4000 m; 17–20 Aug 1923; *R.C. Ching 785* (E00690601) • Qinghai: N of Hushu, Twelve Windings Slopes; mountain slope with *Rhododendron*, *Berberis* and *Potentilla*; 37°01'43.39"N, 102°14'52.93"E; 3064 m; 28 Jul 2010; *B. Paszko 557* (KRA634202) • wild hillside forest; elev. 3800 m; 9 Aug 1983 (KUN0234291) • Shaanxi: Taibai Mountain; hillside wetland; elev. 2400 m; 1 Aug 1956 (KUN0319602) • Xiaoshi Cave, Dongbao, Ningwu; elev. 2000 m; 8 Aug 1984 (MO4741165) • Beside the village of Majia, Ningwu; hillside grassland; 26 Jul 1957; *Shanxi Team* s.n. (MO4486351) • Sichuan: Sikang: Taofu (Dawo) distr., Mt Yara, NW slopes; in silva Larcin; elev. 4000 m; 29 Aug 1934; *H. Smith 11894* (V-040409, MO4365633) • Sikang: Taofu (Dawo) distr., valley west of Mt Yara; in prato herbo­so-fruticoso; elev. 3900 m; 1 Sep 1934; *H. Smith 11791* (V-040410, MO4365640) • Ch’un-ch’e; reg. bor, in silva abietina; elev. 3200 m; 1 Aug 1922; *H. Smith 4129* (V-038567) • inter Mergo et Sankar; reg. bor.-occid. in prato aprico; elev. 3500 m; 3 Sep 1922; *H. Smith 4215* (MO4366937) • Xiangcheng Xian, Reda: Vicinity of the town of Reda; 99°37'55"N, 29°6'11"E; elev. 3450–3650 m; dry slopes with cut over *Quercus*, *Pinus*, *Berberis*, *Cotoneaster*, around *Quercus* in scrubby area; 16 Jul 1998; *D.E. Boufford, B. Bartholomew, C.Y. Chen, M.J. Donoghue, R.H. Ree, H. Sun & S.K. Wu 28773* (MO5308755; E00293189; NY) • Kangding Bridge; hilly terrain; elev. 3600 m; 20 Jul 1963; *K. Guan, W. Wang* s.n. (MO4711607) • Xiangcheng County, Wuming Mountain; elev. 3850 m; 17 Aug 1981 (KUN0234200) • elev. 3500–3600 m; 21 Aug 1985 (KUN0234292) • elev. 4000 m; 31 Aug 1962 (KUN0234195) • elev. 3700–3900 m; 7 Sep 1953 (KUN0234286) • Xizang: Nangqên Xian, Bêca Xiang: along the Ba Qu towards the Xizang border from Bêca Forest Station, SE of Bêcaka; elev. 3600 m; 31°50'N, 96°33'E; *Picealikiangensis* forest, mostly on steep rocky, moss-cov­ered slopes; some areas partially felled, flat areas near river with *Salix* bushland and open disturbed areas, growing among mosses at valley bottom under large *Salix* shrubs; 8 Sep 1996; *T.N. Ho, B. Bartholomew, M. Watson, M. Gilbert 2980* (E00059739) • Yushu Xian: just E of Jiangxi Forest Station on E side of the Zi Qu, SE of Mozhong; elev. 3540 m; 32°5'N, 97°1'E; growing under *Picea*; 27 Aug 1996; *T.N. Ho, B. Bartholomew, M. Watson, M. Gilbert 2479* (E00061514, MO5094288). – India • Kashmir: above Gulmarg; elev. 12000 ft.; Aug 1926; *R.R. Stewart 8788* (NY).

##### Note.

During the revision of the herbarium materials, we found two specimens of *P.chingii* collected from Bhutan that were identified as *P.bhutanica*. Similar misidentification was mentioned by Zhang et al. (2016b) in the case of *P.bhutanica* collected in China (but misidentified as *P.chingii*). The two species, however, differ in the ratio of lemma to palea length, a difference that is apparently more pronounced in *P.bhutanica*. Other features to distinguish among the two species are the number of vascular bundles present in leaf cross-section, where *P.chingii* usually has 5, while *P.bhutanica* 7 and the length of hairs on the seta being 0.2–0.3 and 0.3–0.5 mm, respectively.

Within *P.chingii*, specimens with panicles contracted to loosely contracted and with panicles open are observed. Specimens with contracted to loosely contracted panicles represent the typical variety P.chingiivar.chingii, whereas the second one, with open panicles (Suppl. material 2: fig. S14), is here recognised as a distinct variety.

#### ﻿ Ptilagrostis chingii var. laxum

Taxon classificationPlantaePoalesPoaceae

(S.L.Lu) M.Nobis & Krzempek comb. nov.

7E032474-FD89-5BFA-A0E8-708A4D76AE71

urn:lsid:ipni.org:names:77351834-1

Achnatherum chingii var. laxum S.L. Lu, Acta Biologica Plateau Sinica 2: 19. 1984. **Basionym.**
≡ Achnatherumchingiivar.laxum P. C. Kuo & S. L. Lu, Flora Xizangica 5: 257. 1987. nomen illeg. homonym.

##### Type.

China: Sichuan: Sertara, 10 Sept. 1961, Q.L. Zhang 350163 (holotype: NJU).

##### Selected specimens studied of P.chingiivar.laxum.

China • Sichuan: Prov. Sze-ch’uan; reg. bor.-occid.: inter Mergo et Sankar in prato apricot, reg. bor.-oc­cid; elev. 3500 m; 3 Sep 1922; *H. Smith 4215* (MO4366937) • Xizang: Yushu Xian: just E of Jiangxi Forest Station on E side of the Zi Qu, SE of Mozhong; elev. 3540 m; 32°5'N, 97°1'E; growing under *Picea*; 27 Aug 1996; *T.N. Ho, B. Bartholomew, M. Watson, M. Gilbert 2479* (E00061514, MO5094288).

#### ﻿14. Ptilagrostis duthiei

Taxon classificationPlantaePoalesPoaceae

(Hook.f.) M. Nobis & P.D. Gudkova, PhytoKeys 128: 107. 2019.

163CBB1D-F8CA-52D8-A776-331F6F7F874F

Stipa duthiei Hook.f., Flora of British India 7: 232. 1896. **Basionym.**
≡ Achnatherumduthiei (Hook.f.) Kuo & Lu, Flora Reipublicae Popularis Sinicae 9(3): 322, pl. 80, f. 9–14. 1987.

##### Type.

[India] Tehri Garwhal, Lekhus, below Srikanta, 12000–13000 ft, 11 Aug. 1853, *Duthie 273* (holotype: K 32097!, isotype CAL 2350!).

##### Description.

***Perennial plants***, densely tufted, with a few culms and numerous vegetative shoots; culms (40–)60–100(–110) cm tall, 3-noded, distributed below the middle of the culm, exerted from leaf-sheaths. ***Leaves of vegetative shoots***: sheaths glabrous; ***ligules*** lanceolate, on the external sheaths (0.5–)1.0–1.4(–1.5) mm long, whereas on the internal sheaths, (0.5–)0.9–1.7 mm long; ***blades*** convolute, green, pale green to greyish, (17.4–)31.7–41.4(–52.5) cm long, (0.5–)0.6–1.0 mm in diameter, with (7–)9–11 vascular bundles, adaxial surface covered by 0.15–0.25 mm long hairs, abaxial surface glabrous or less frequently minutely scabrous. ***Cauline leaves***: sheaths glabrous or less often slightly scabrous; ***ligules*** on the lower sheaths lanceolate (0.8–)1.5–2.0(–2.6) mm long, on the middle and upper sheaths (1.9–)2.0–2.2 and 2.2–2.9(–3.9), respectively; ***blades*** convolute, green, pale green or greyish, adaxial surface covered with short hairs, abaxial surface glabrous or less frequently scabrous. ***Panicle*** open, 20.3–22.5(–25.6) cm long; ***branches*** ascending, usually scabrous, single or paired, lower branch (4.8–)5.7–9.0(–10.7) cm long. ***Glumes*** subequal, the lower 0.2–0.5 mm longer than the upper, yellowish, brown, green or purple, lower glume (9.3–)9.5–11.0(–11.8) mm long, upper glume 9.0–11.0(–11.6) mm long, lanceolate. ***Floret*** (lemma + callus) (6.2–)6.5–7.2(–7.5) mm long. ***Callus*** 0.5–0.6(–0.7) mm long, densely pilose, on ventral part with hairs (0.3–)0.4–0.6(–0,8) mm long, on dorsal with (0.3–)0.4–0.5 mm long hairs; callus base (0.3–)0.4–0.6 mm long and 0.3–0.4(–0.5) mm in diameter, obtuse. ***Lemma*** coriaceous, pale-green, purplish or brownish, covered from the bottom up to 1/3 of its length, by dense ascending to appressed hairs 0.3–0.4(–0.5) mm long, hairless in the mid-length and with hairs at apex; ***lemma apex*** with unequal hairs 0.2–0.3(–0.4) mm long and with two minute apical lobes 0.2–0.3(–0.5) mm long. ***Palea*** equal or slightly, 0.1–0.3 mm shorter than lemma in length. ***Awn*** (12–)14–16(–17) mm long, unigeniculate; ***the lower segment of the awn* (*column*)** (4–)5–6(–7) mm long, twisted, with (0.4–)0.5–0.6 mm long hairs; ***terminal segment of the awn* (*seta*)** straight, (8–)9–10(–11) mm long, scabrous, at base with hairs 0.2–0.3 mm long, gradually decreasing in length towards the apex. ***Anthers*** ca. 3–4 mm long, bearded at the apex.

##### Phenology.

Flowering from July to September.

##### Figures.

**Figure 21.**
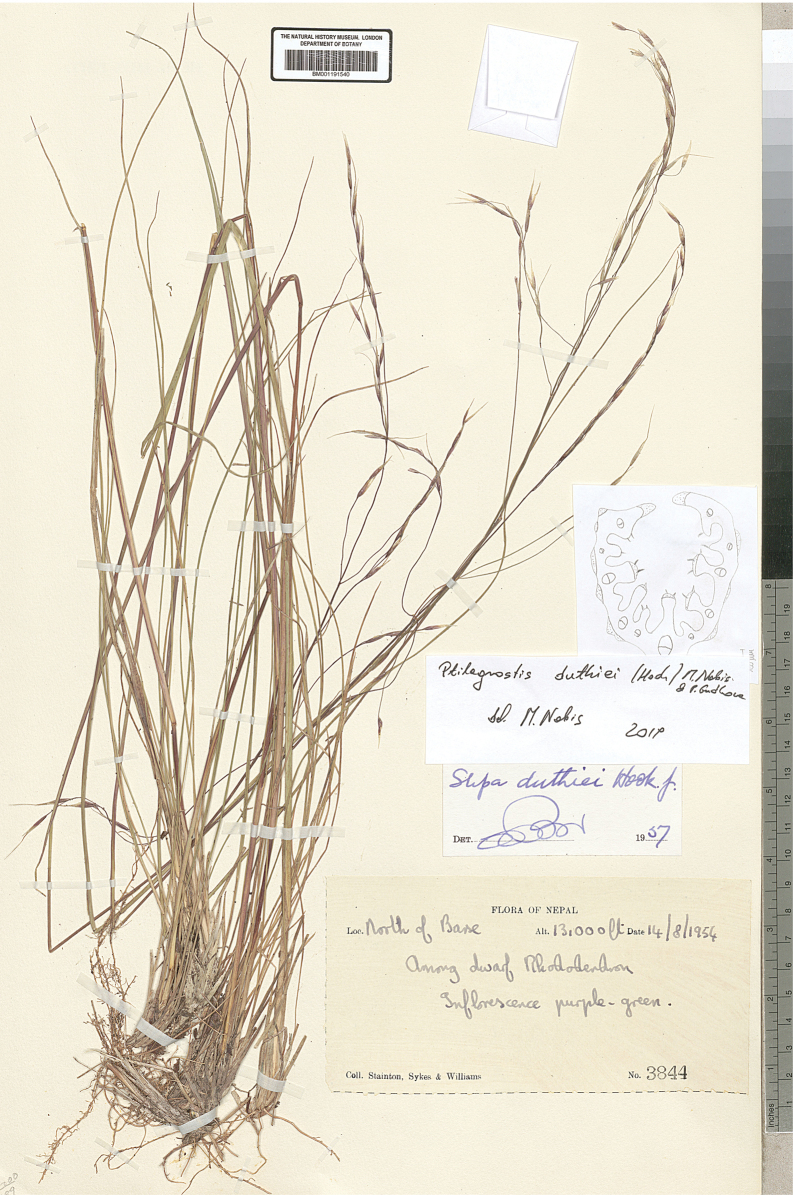
*Ptilagrostisduthiei*, general habit.

Figs 4m-o, 6a, b, 21; additional figures in Wu et al. (2007: fig. 281); http://www.efloras.org/object_page.aspx?object_id=94972&flora_id=2; https://powo.science.kew.org/taxon/urn:lsid:ipni.org:names:77200949-1; https://www.gbif.org/species/10596562.

##### Distribution.

Bhutan, India, Nepal, China: Qinghai, Shaanxi, Sichuan, Xizang, Yunnan (Freitag 1985; Noltie 2000; Wu and Phillips 2006; Nobis et al. 2019b).

##### Habitat.

Alpine grasslands, shrublands, coniferous and mixed forests, at (2500–)3500–4000(–4500) m elev.

##### Selected studied specimens of *P.duthiei*.

China • Shaanxi; elev. 3650 m; 16 Jul 1963 (KUN0323199) • Sichuan; E Tibet, Litang - Batang, Jinsha (Yangtse) tributary, E of Yidun/Yarw; 30°15’ N, 99°25'E; mont. moist *Salix* scrub by stream; elev. 3640 m; 25 Jun 1994; *B. Dickoré 8343* (MSB-152907) • Sikang, between Taining (Ngata) and Taofu (Dawo), Sunglingk; in silva muscosa abietina; elev. 3900 m; 11 Sep 1934; *H. Smith 12034* (V-040408, MO4365639) • Sikang, Kang­ting (Tachienlu) distr., Chungo valley, Mt Yara, NE slope; in silva mixta; elev. 3900 m; 18 Aug 1934; *H. Smith 11145* (V-040407) • Sze-ch’uan, reg. bor., Don­grergo (Hsioeh-pau-ting); in silva mixta; elev. 4000 m; 20 Jul 1922; *H. Smith 3797* (V-040980) • elev. 3650 m; 16 Jul 1963 (KUN0323199) • Sikang, Kangting (Tachienlu) distr.: Chungo valley: in jugo bor.-orient. montis Yara; in silva mix­ta; elev. 3900 m; 18 Aug 1934; *H. Smith 11145* (MO4365638). – Nepal • North of Barse; among dwarf *Rhododendron*; elev. 13,000 ft.; 14 Aug 1954; *Stainton, Sykes & Williams 3844* (BM001191540, E00690624).

#### ﻿15. Ptilagrostis contracta

Taxon classificationPlantaePoalesPoaceae

Z.S. Zhang & W.L. Chen, PlosOne, 12(1): e0166603: 3–4. 2017.

55E7180C-889B-5D21-8254-03C78361FEB0

##### Type.

China. Sichuan: Litang, elev. 3701 m, 26 Sep 2014, *Z.S*. *Zhang & L.L*. *Li 341* (holotype: PE).

##### Description.

***Perennial plants***, densely tufted, with a few culms and numerous vegetative shoots; culms 57–105 cm tall, 3-noded distributed below the middle of the culm, exerted or hidden by the leaf-sheaths. ***Leaves of vegetative shoots***: sheaths glabrous; ***ligules*** lanceolate, on the external sheaths 0.5–0.6 mm long, whereas on the internal sheaths, 1.0–2.1 mm long; ***blades*** convolute, green, pale green to greyish, 27.2–30.3 cm long, (0.6–)0.7–1.2 mm in diameter, with 11–14 vascular bundles, adaxial surface covered by 0.15–0.25 mm long hairs, abaxial surface glabrous and smooth. ***Cauline leaves***: sheaths glabrous; ***ligules*** on the lower sheaths lanceolate 0.6–1.1 mm long, on the middle and upper sheaths 1.5–1.6 and 1.8–2.1, respectively; ***blades*** convolute, green, pale green or greyish, adaxial surface covered with short hairs, abaxial surface glabrous. ***Panicle*** contracted, 13–31 cm long; ***branches*** ascending, glabrous, single or paired, lower branch 2.7–4.5 cm long. ***Glumes*** subequal, the lower slightly 0.2–0.4 mm longer than the upper, yellowish, brown, green or purple, lower glume 10.0–14.0 mm long, upper glume 10.0–14.0 mm long, lanceolate. ***Floret*** (lemma + callus) 7.0–8.3 mm long. ***Callus*** 0.7 mm long, densely pilose, on ventral part with hairs 0.3– 0.5 mm long, on dorsal with 0.4 mm long hairs; callus base 0.5 mm long and 0.3–0.5 mm in diameter, obtuse. ***Lemma*** coriaceous, pale-green, purplish or brownish, covered from the bottom up to 1/3 of its length, by dense ascending to appressed hairs 0.4–0.5 mm long, hairless in the mid-length and with hairs at apex; ***lemma apex*** with unequal hairs 0.5–0.6 mm long and with two apical lobes 0.5–0.6 mm long. ***Palea*** slightly, 0.1–0.2 mm shorter lemma in length. ***Awn*** 12–16(–20) mm long, unigeniculate; ***the lower segment of the awn* (*column*)** 4–7 mm long, twisted, with 0.4–0.6 mm long hairs; ***terminal segment of the awn* (*seta*)** straight, 7–9 mm long, scabrous, at base with 0.2(–0.3) mm long, gradually decreasing in length towards the apex. ***Anthers*** 2.5–3.0 mm long, bearded at the apex.

##### Phenology.

Flowering from July to September.

##### Figures.

**Figure 22.**
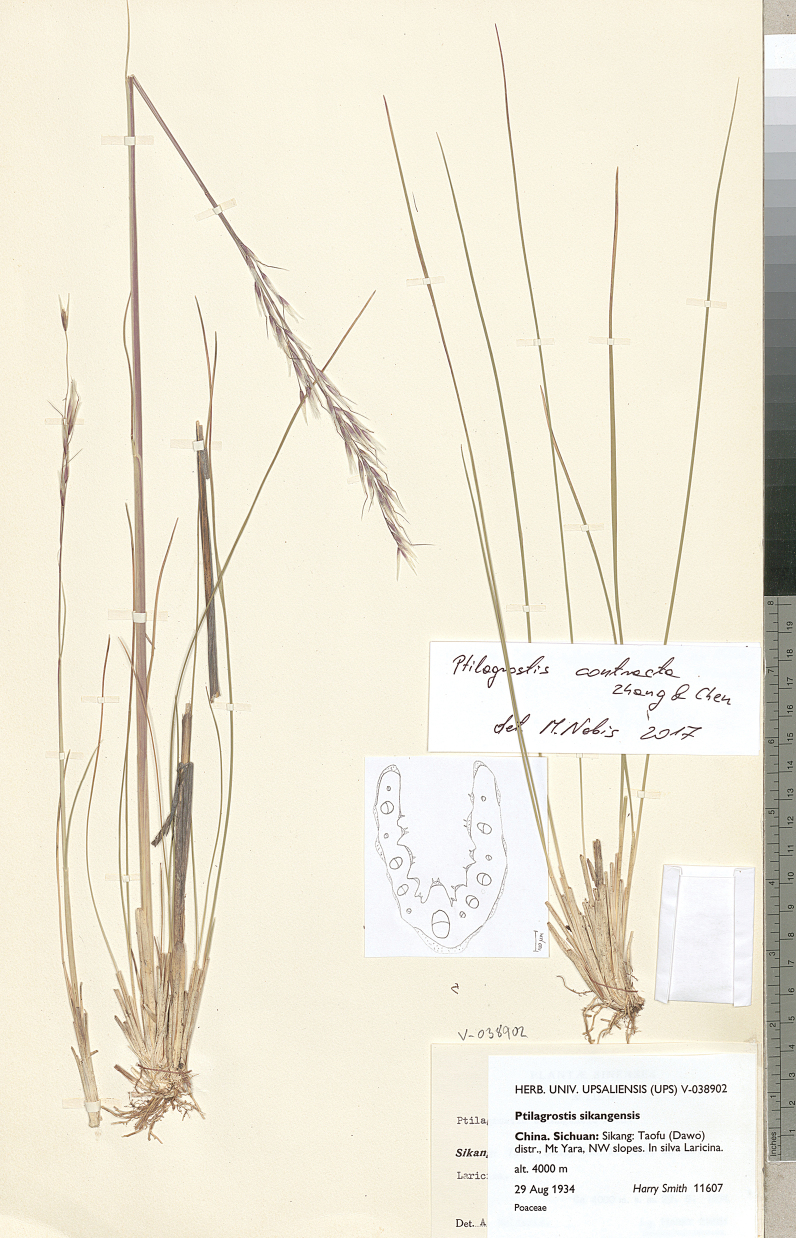
*Ptilagrostiscontracta*, general habit.

Figs 4j–l, 6c, d, 22; additional figures in Zhang et al. (2017: fig. 1, 3).

##### Distribution.

China: Sichuan (Zhang et al. 2017).

##### Habitat.

Alpine grasslands, thickets and forests, at 3500–4300 m elev.

##### Selected studied specimens of *P.contracta*.

China • Sichuan: Sikang: Taofu (Dawo) distr., Haitzeshan; in prato herboso-fruticoso; elev. 3900 m; 26 Aug 1934; *H. Smith 11589* (V-040415) • Sikang: Taofu (Dawo) distr., Mt Yara, NW slopes; in silva *Larcina*; elev. 4000 m; 29 Aug 1934; *H. Smith 11607* (V-038902) • Hei-tze-shan; in the lake side; elev. 4600 m; 29 Aug 1934; *C.S. Liu* s.n. (PE 00052432, PE00052433) • Rangtang County, Peng Du; subalpine shrub meadow in the mid­dle of the valley; elev. 4100 m; 17 Jul 1975 (PE00052429).

### ﻿Species excluded from *Ptilagrostis*

*Ptilagrostiskingii* (Bol.) Barkworth = ***Ptilagrostiellakingii*** (Bol.) Romasch. [basionym *Stipakingii* Bol.; ≡ *Oryzopsiskingii* (Bol.) Beal; Peterson et al. 2019].

*Ptilagrostispurpurea* (Griseb.) Roshev. = ***Stipapurpurea*** Griseb. [Nobis et al. 2020, 2022].

*Ptilagrostispelliotii* (Danguy) Grubov = ***Achnatherumpelliotii*** (Danguy) Röser & H.R. Hamasha [basionym *Stipapelliotii* Danguy; Hamasha et al. 2012].

*Ptilagrostissemenovi* Krasn. = ***Stipatremula*** (Rupr.) M. Nobis [=*Stipasemanowii* Krassn.; Nobis et al. 2022].

*Ptilagrostissubsessiliflora* (Rupr.) Roshev. = ***Stipasubsessiliflora*** (Rupr.) Roshev. [Tzvelev 1976, Freitag 1985, Nobis et al. 2020].

## Supplementary Material

XML Treatment for Ptilagrostis

XML Treatment for Ptilagrostis sect. Ptilagrostis

XML Treatment for Ptilagrostis alpina

XML Treatment for Ptilagrostis arcuata

XML Treatment for Ptilagrostis concinna

XML Treatment for Ptilagrostis concinna var. xizangensis

XML Treatment for Ptilagrostis dichotoma

XML Treatment for Ptilagrostis glabrifolia

XML Treatment for Ptilagrostis glabrifolia var. himalayensis

XML Treatment for Ptilagrostis junatovii

XML Treatment for Ptilagrostis junatovii var. schischkinii

XML Treatment for Ptilagrostis luquensis

XML Treatment for Ptilagrostis malyschevii

XML Treatment for Ptilagrostis mongholica

XML Treatment for Ptilagrostis mongholica subsp. mongholica

XML Treatment for Ptilagrostis mongholica subsp. porteri

XML Treatment for Ptilagrostis tibetica

XML Treatment for Ptilagrostis sect. Barkworthia

XML Treatment for Ptilagrostis yadongensis

XML Treatment for Ptilagrostis bhutanica

XML Treatment for Ptilagrostis sect. Chenella

XML Treatment for Ptilagrostis chingii

XML Treatment for Ptilagrostis chingii var. laxum

XML Treatment for Ptilagrostis duthiei

XML Treatment for Ptilagrostis contracta

## ﻿Additional information

### Author ORCIDs

Marta Krzempek https://orcid.org/0000-0001-9608-7259

Ewelina Klichowska https://orcid.org/0000-0001-9641-5750

Marcin Nobis https://orcid.org/0000-0002-1594-2418

### Data availability

The SNP dataset derived from the DArTseq pipeline in the genlight format is available via Figshare repository, https://figshare.com/s/2f39354c7ead1305e705.

## Supplementary materials

Supplementary material 1 Insights to the taxonomy and phylogeny of the genus *Ptilagrostis* worldwide (Poaceae, Stipeae) with a key to species identification, checklist and outlines for further studies Data type xlsx https://binary.pensoft.net/file/1175128This dataset is made available under the Open Database License (http://opendatacommons.org/licenses/odbl/1.0/). The Open Database License (ODbL) is a license agreement intended to allow users to freely share, modify, and use this Dataset while maintaining this same freedom for others, provided that the original source and author(s) are credited.Marta Krzempek, Ewelina Klichowska, Marcin Nobis

Supplementary material 2 Fourteen additional images Data type docx Explanation note **fig. S1.** Cluster analysis (UPGMA) of all examined specimens of all Ptilagrostis species. List of specimens examined is presented in Suppl. material 1. **fig. S2.** The holotype of *Ptilagrostisalpina* (LE). **fig. S3.** The isotype of *Ptilagrostisarcuata* (K). **fig. S4.** The isotype of *Ptilagrostisconcinna* (LE). **fig. S5.** The holotype of Ptilagrostisconcinnavar.xizangensis (MSB). **fig. S6.** The isotype of *Ptilagrostisdichotoma* (LE). **fig. S7.** The holotype of Ptilagrostisdichotomavar.roshevitsiana (LE). **fig. S8.** The holotype of *Ptilagrostisjunatovii* (LE). **fig. S9.** The holotype of Ptilagrostisconcinnasubsp.schischkinii (LE). **fig. S10.** The holotype of *Ptilagrostismalyschevii* (LE). **fig. S11.** The lectotype of Ptilagrostismongholicasubsp.mongholica (LE). **fig. S12.** The lectotype of *Ptilagrostistibetica* (K). **fig. S13.** The isotype of *Ptilagrostischingii* (E). **fig. S14.**Ptilagrostischingiivar.laxum, general habitat (E). https://binary.pensoft.net/file/1175129This dataset is made available under the Open Database License (http://opendatacommons.org/licenses/odbl/1.0/). The Open Database License (ODbL) is a license agreement intended to allow users to freely share, modify, and use this Dataset while maintaining this same freedom for others, provided that the original source and author(s) are credited.Marta Krzempek, Ewelina Klichowska, Marcin Nobis
