# Supplementary material for: ﻿Insights to the taxonomy and phylogeny of the genus Ptilagrostis worldwide (Poaceae, Stipeae) with a key to species identification, checklist and outlines for further studies
Source: PhytoKeys. 2024 Nov 15;249:115–80. doi: 10.3897/phytokeys.249.128729 (PMC11584906; doi:10.3897/phytokeys.249.128729)
Supplement: Supplementary material 2 — Fourteen additional images [file phytokeys-249-115_article-128729__-s002.docx]

**Insights to the taxonomy and phylogeny of the genus *Ptilagrostis* worldwide (Poaceae, Stipeae) with a key to species identification, checklist and outlines for further studies**

Marta Krzempek*, Ewelina Klichowska, Marcin Nobis*

Institute of Botany, Faculty of Biology, Jagiellonian University, Gronostajowa 3,

Kraków 30–387, Poland.

corresponding authors: Marta Krzempek (marta.krzempek@doctoral.uj.edu.pl), Marcin Nobis (m.nobis@uj.edu.pl)

**Phytokeys 2024**

Appendis 2: Figures 1-14.


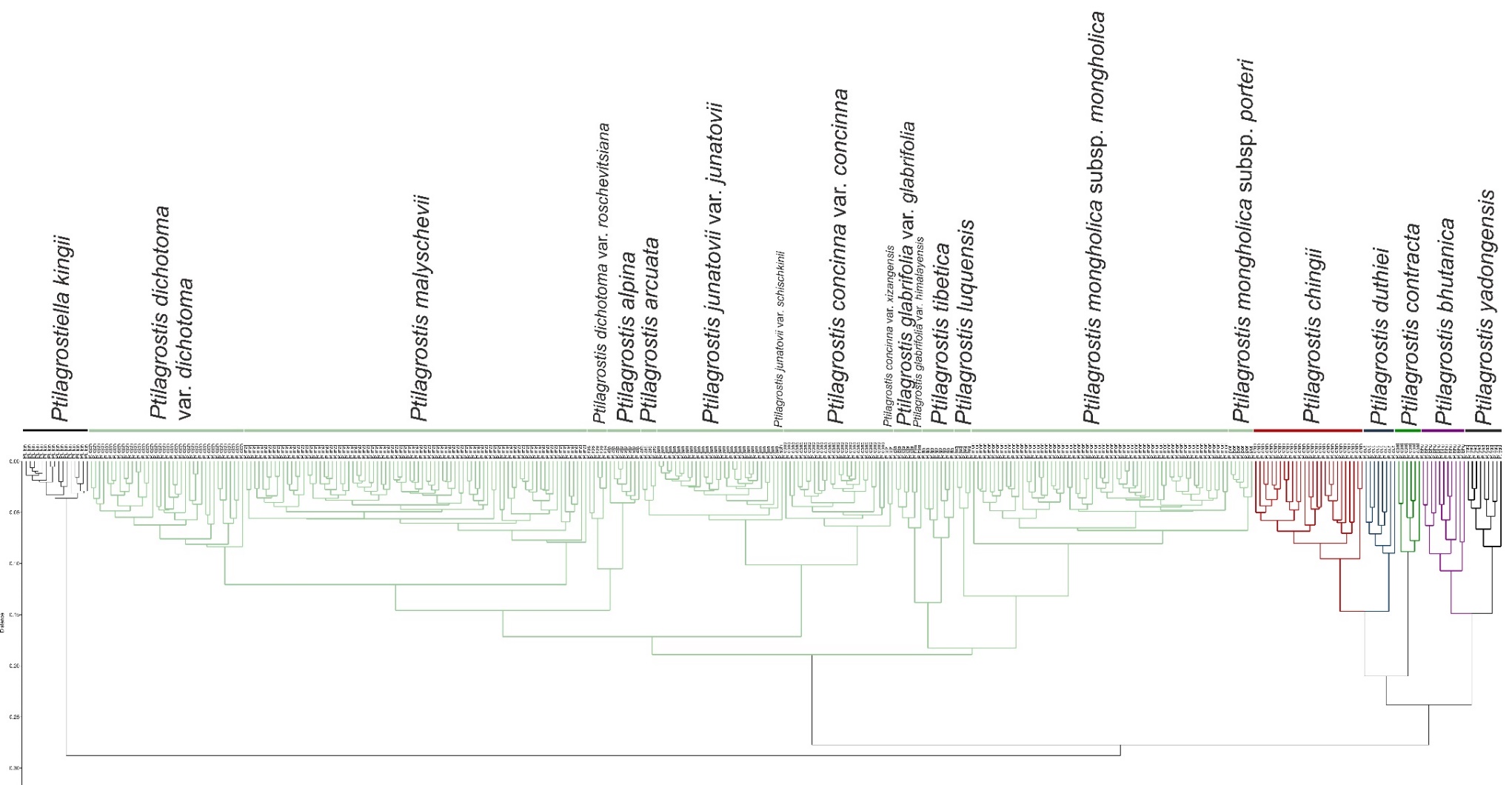


Figure 1. Cluster analysis (UPGMA) of all examined specimens of all *Ptilagrostis* species. List of specimens examined is presented in Appendix 1.


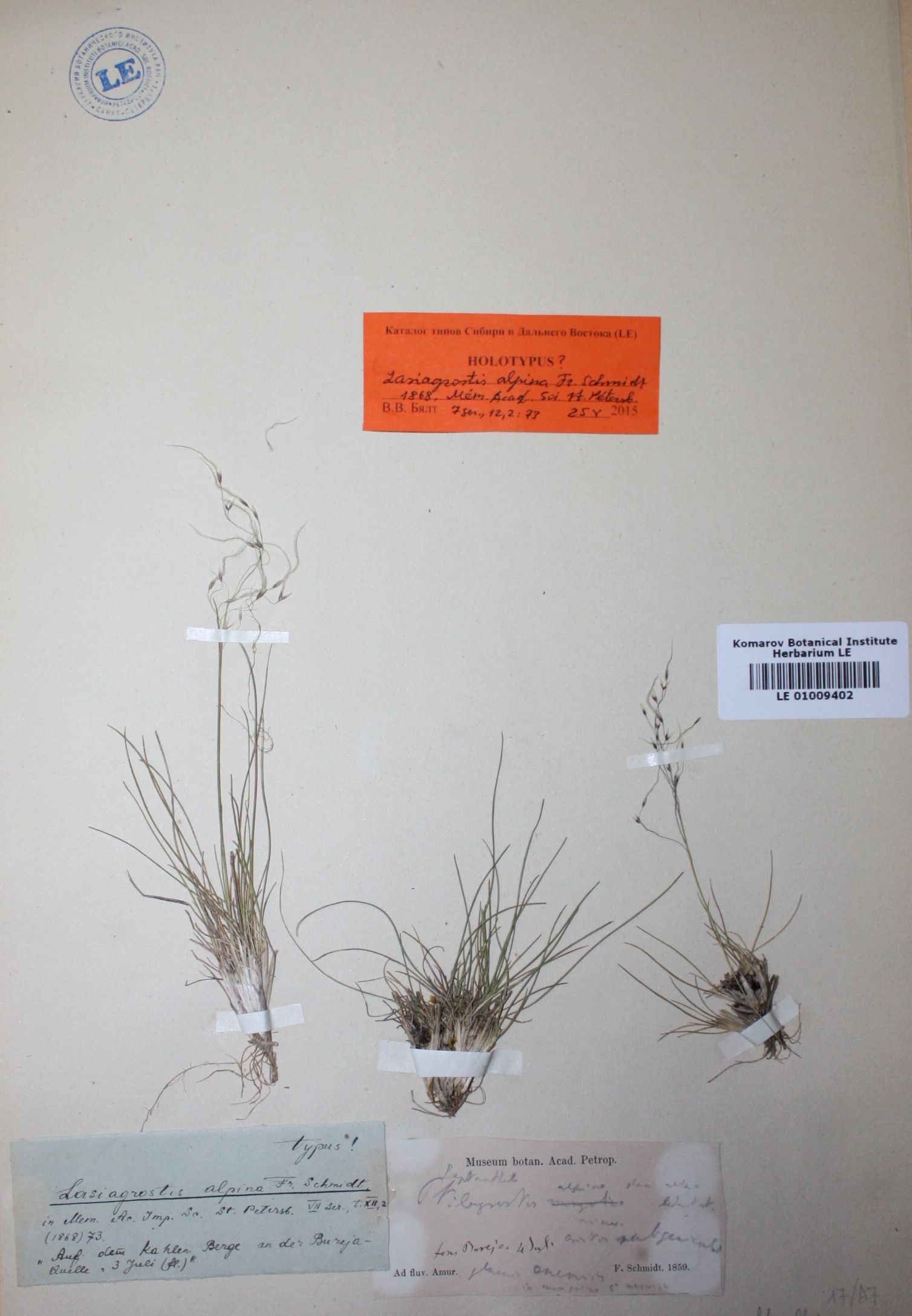


Figure 2. The holotype of *Ptilagrostis alpina* (LE).


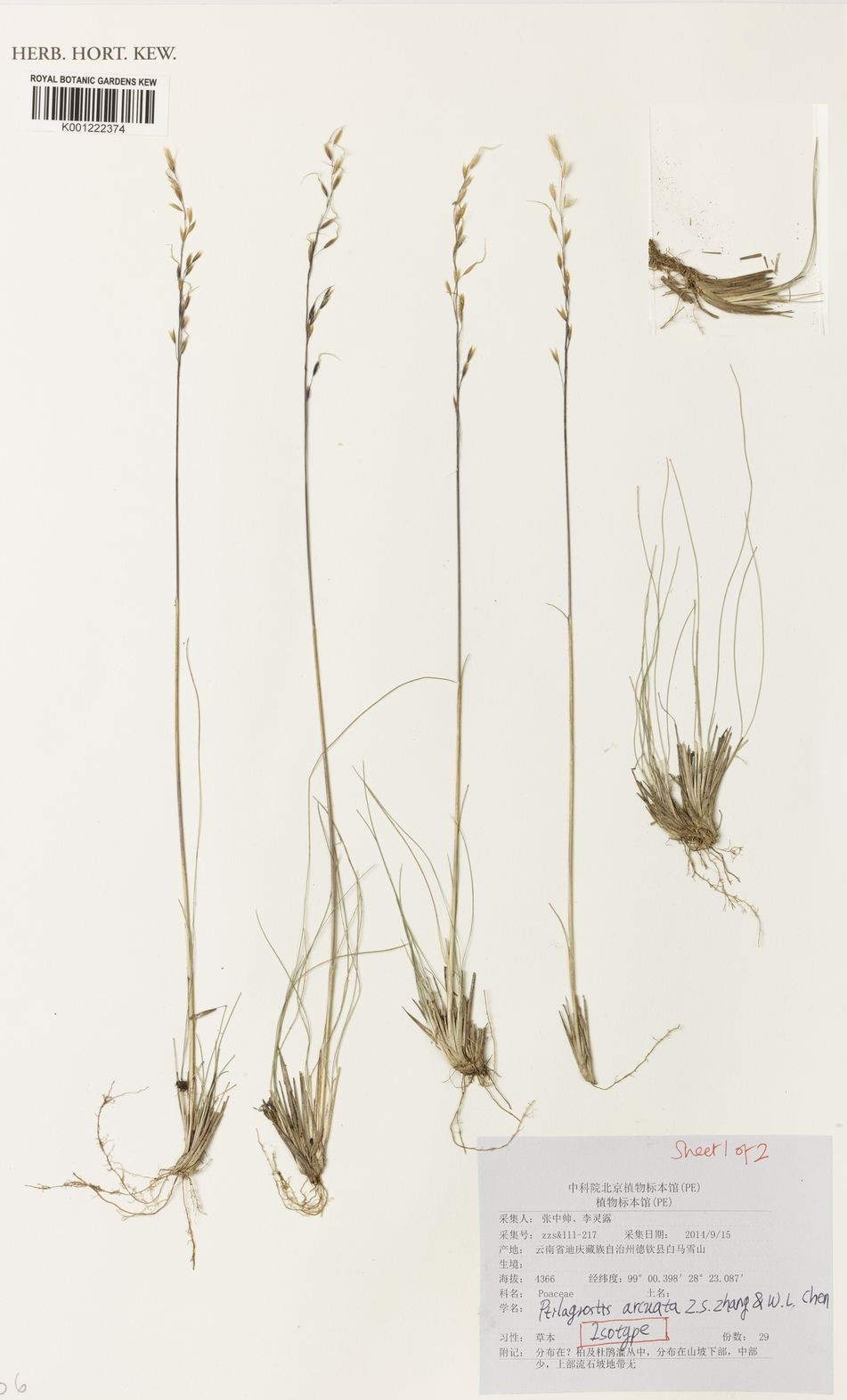


Figure 3. The isotype of *Ptilagrostis arcuata* (K).


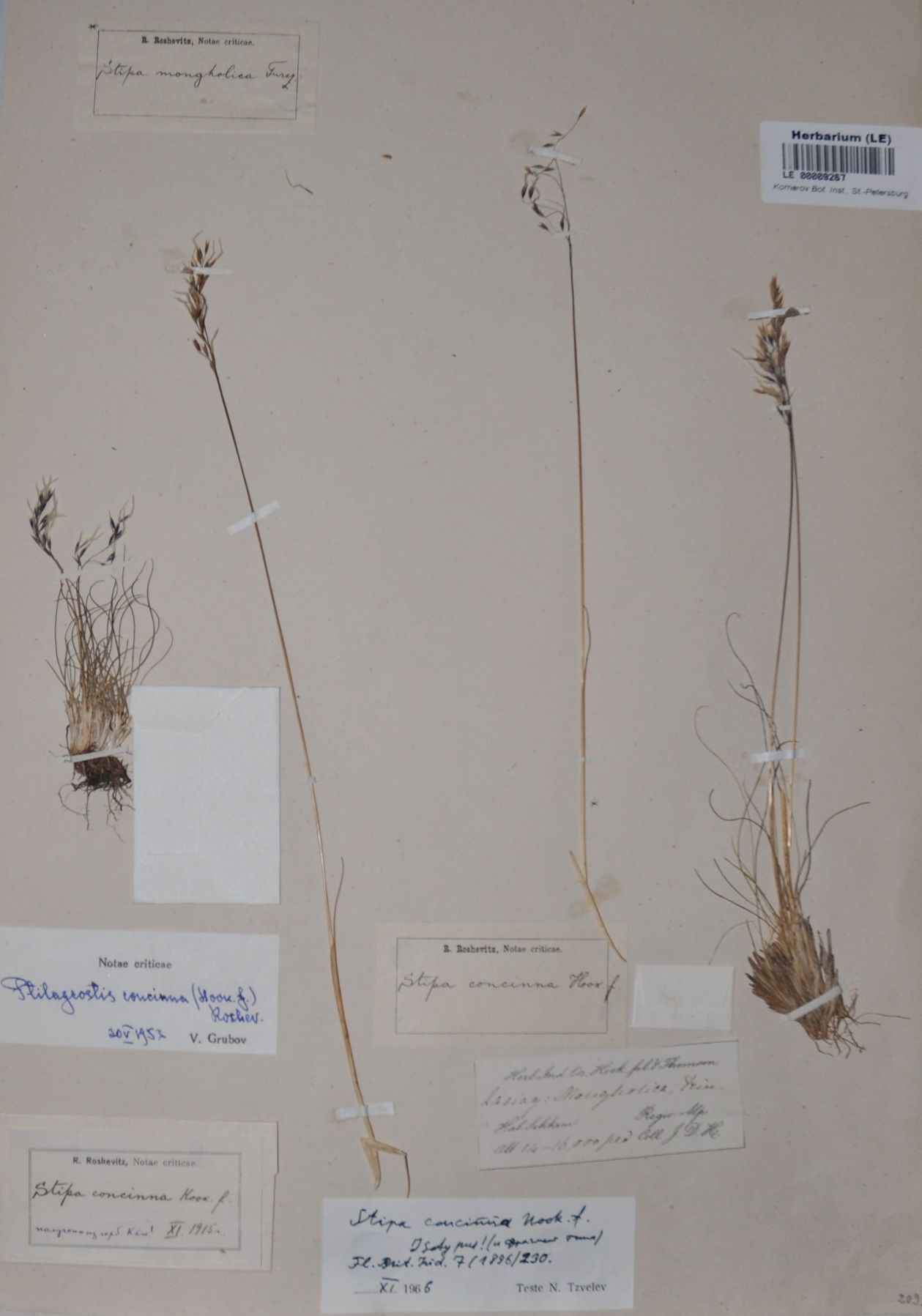


Figure 4. The isotype of *Ptilagrostis concinna* (LE).


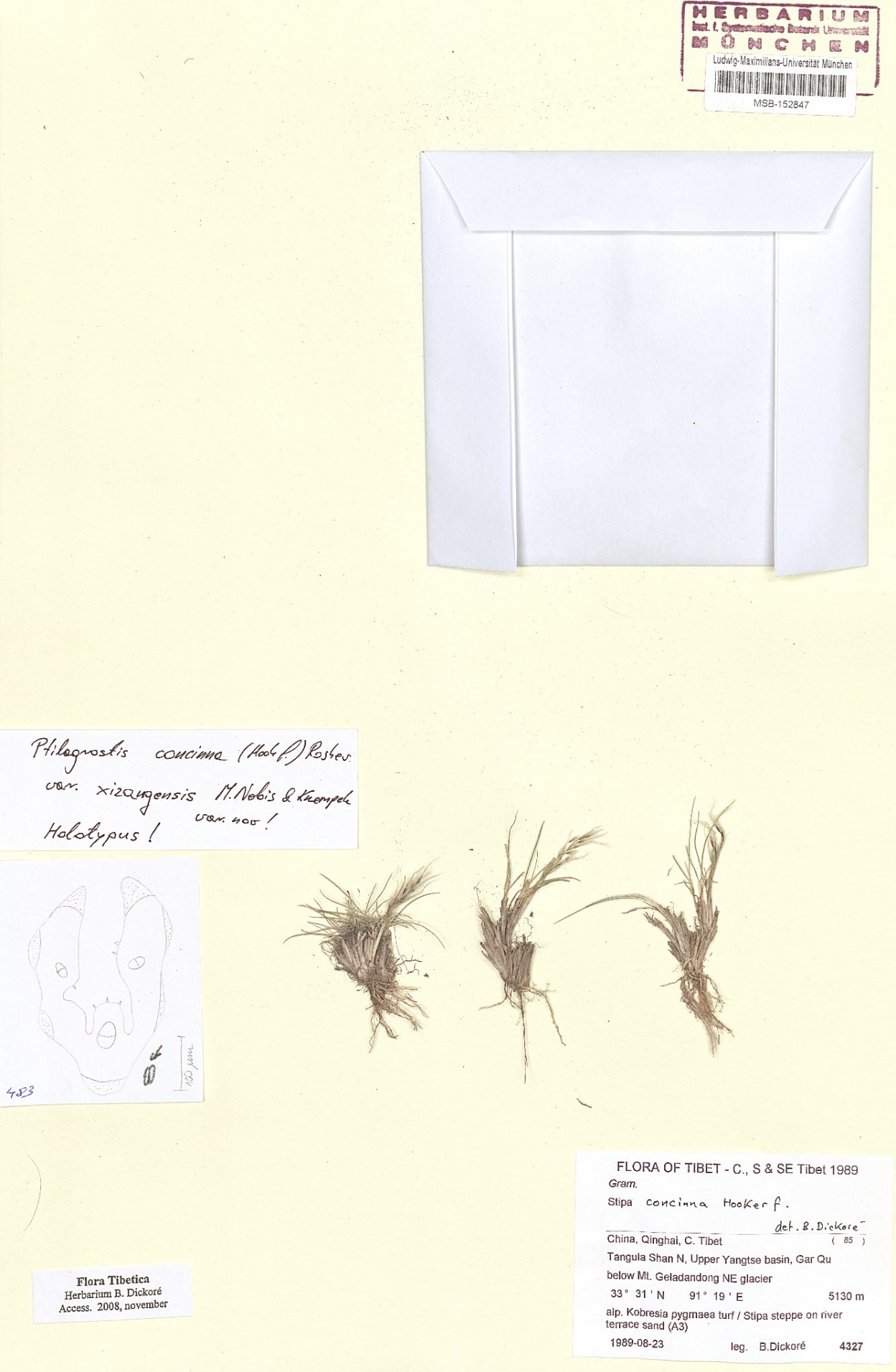


Figure 5. The holotype of *Ptilagrostis concinna* var. *xizangensis* (MSB).


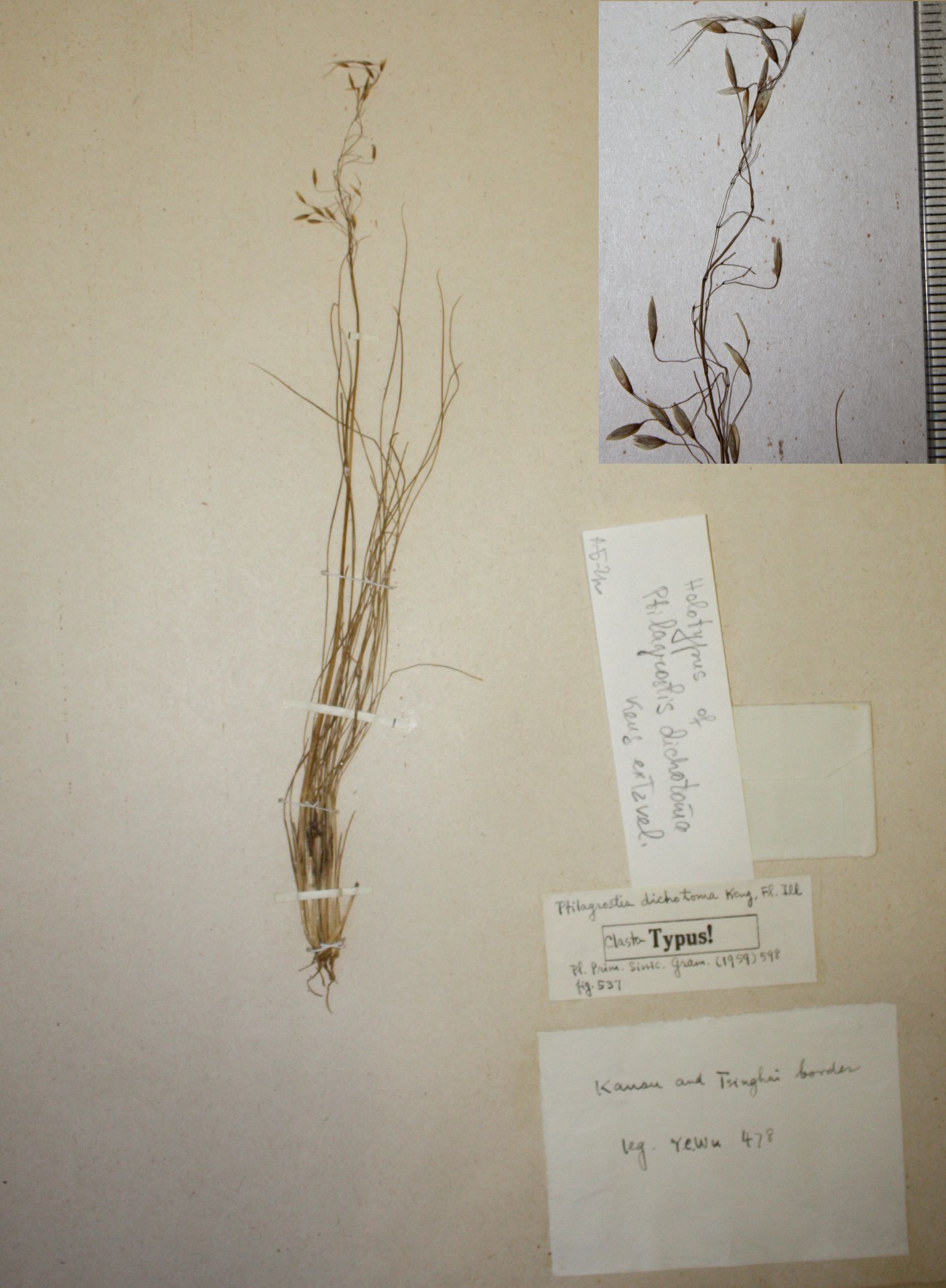


Figure 6. The isotype of *Ptilagrostis dichotoma* (LE).


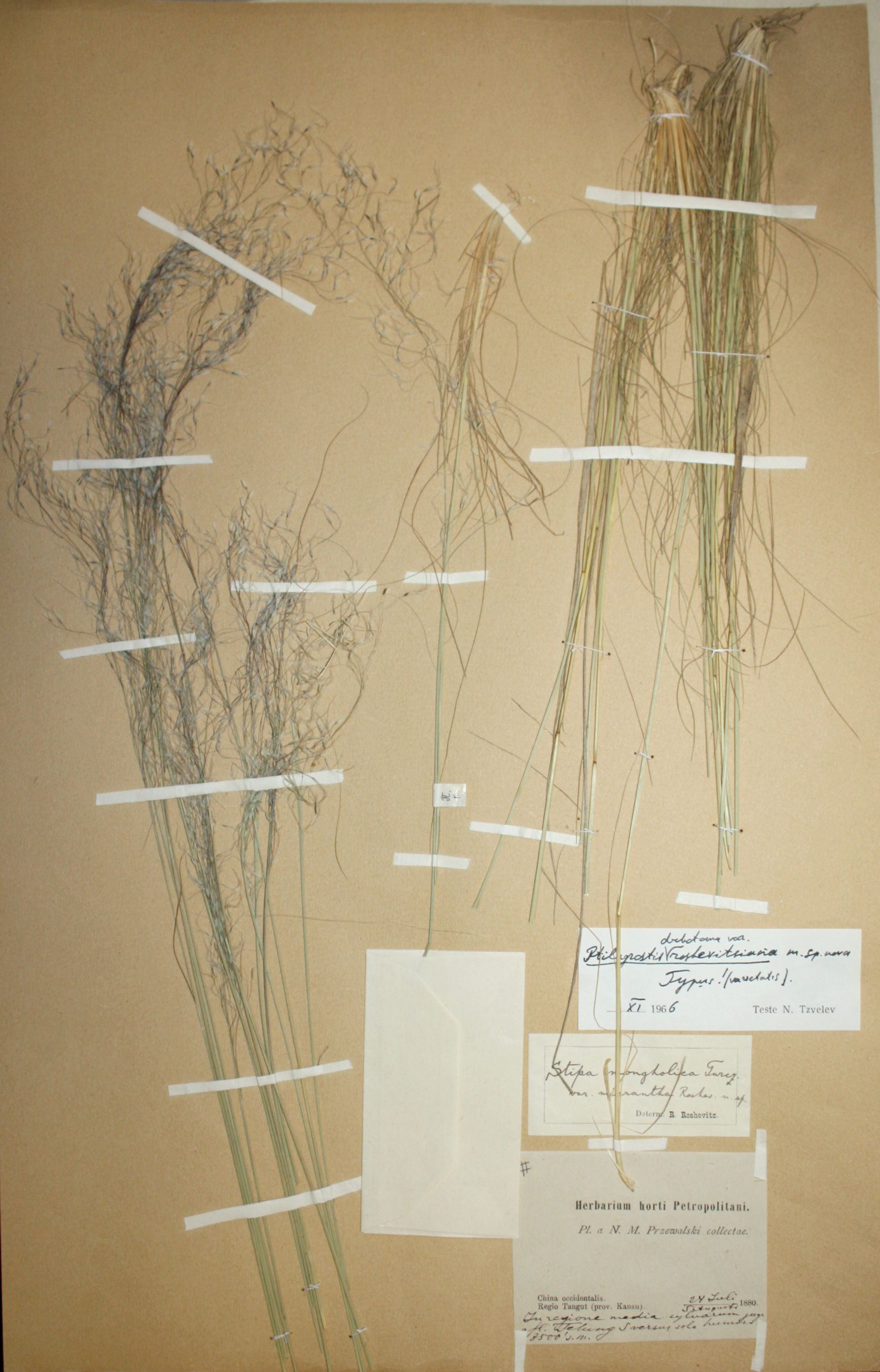


Figure 7. The holotype of *Ptilagrostis dichotoma* var. *roshevitsiana* (LE).


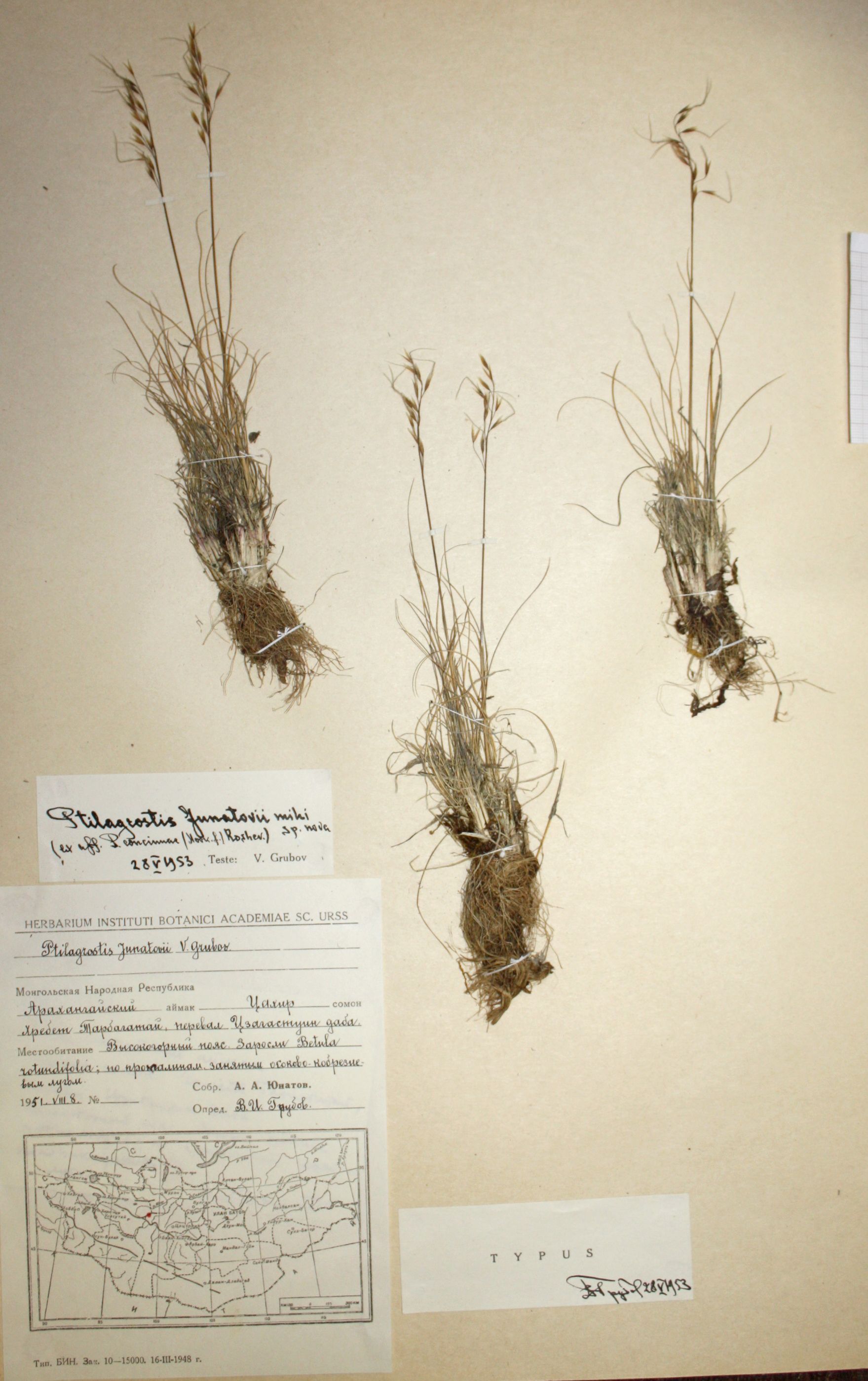


Figure 8. The holotype of *Ptilagrostis junatovii* (LE).


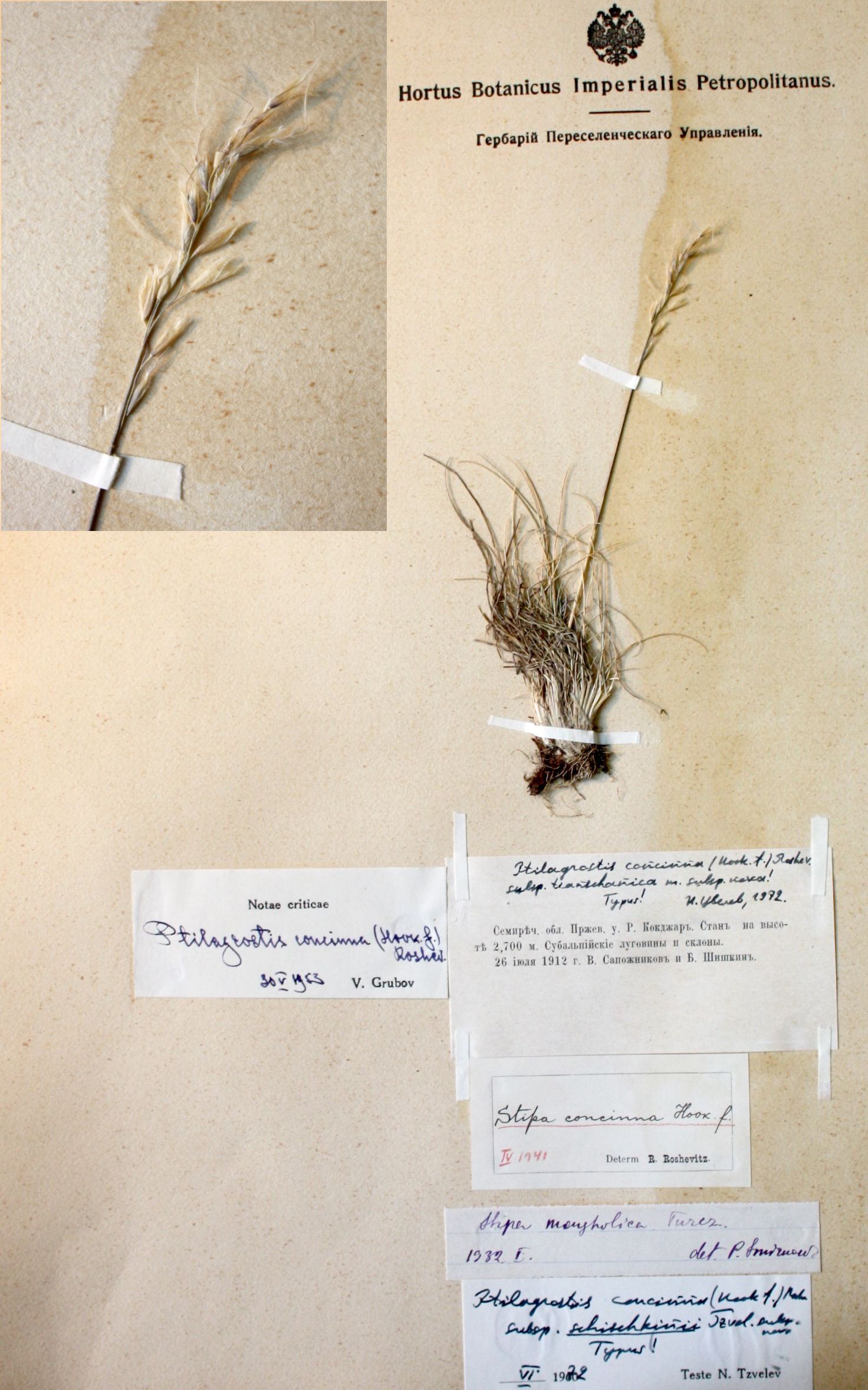


Figure 9. The holotype of *Ptilagrostis concinna* subsp. *schischkinii* (LE).


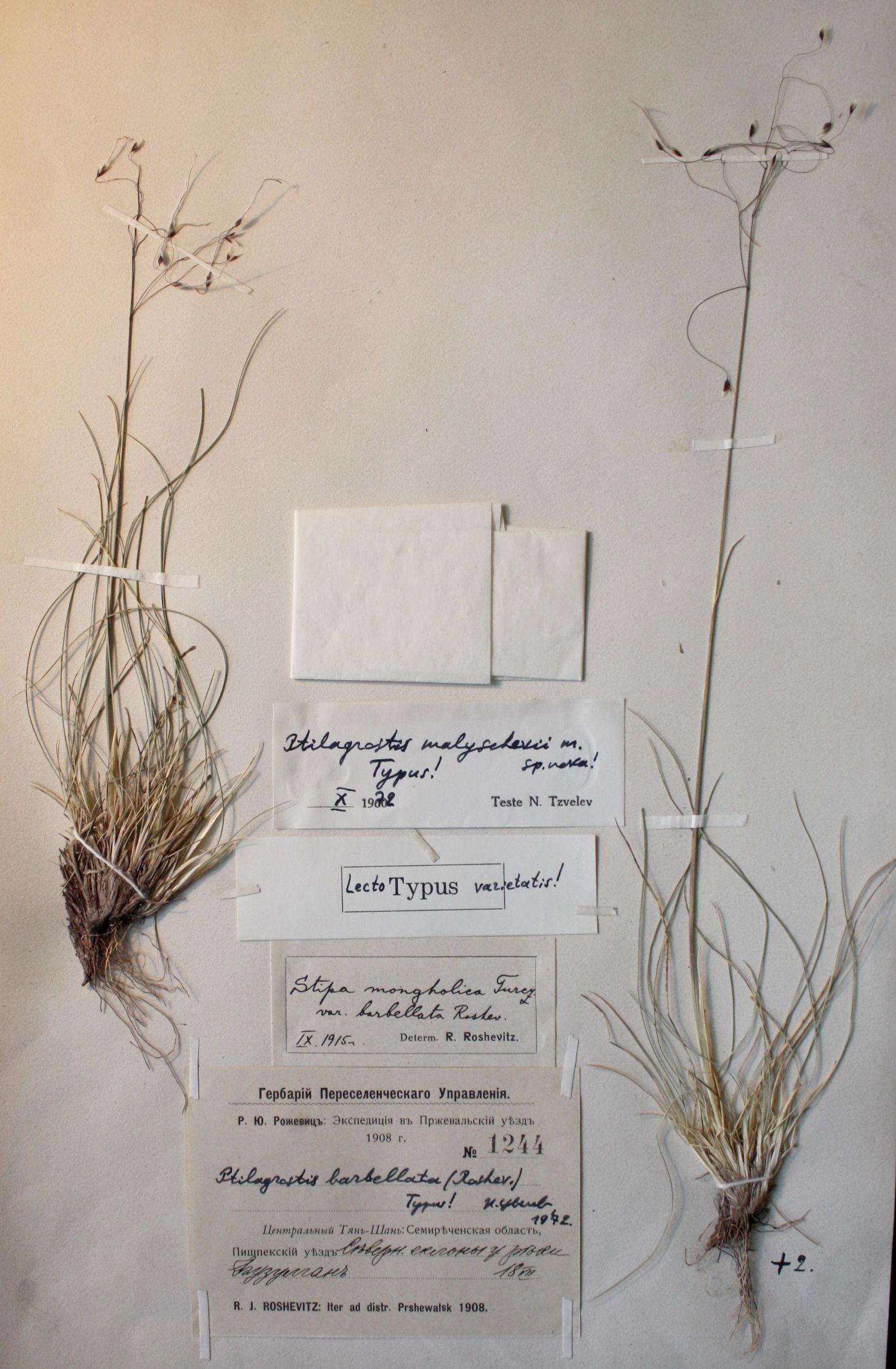


Figure 10. The holotype of *Ptilagrostis malyschevii* (LE).


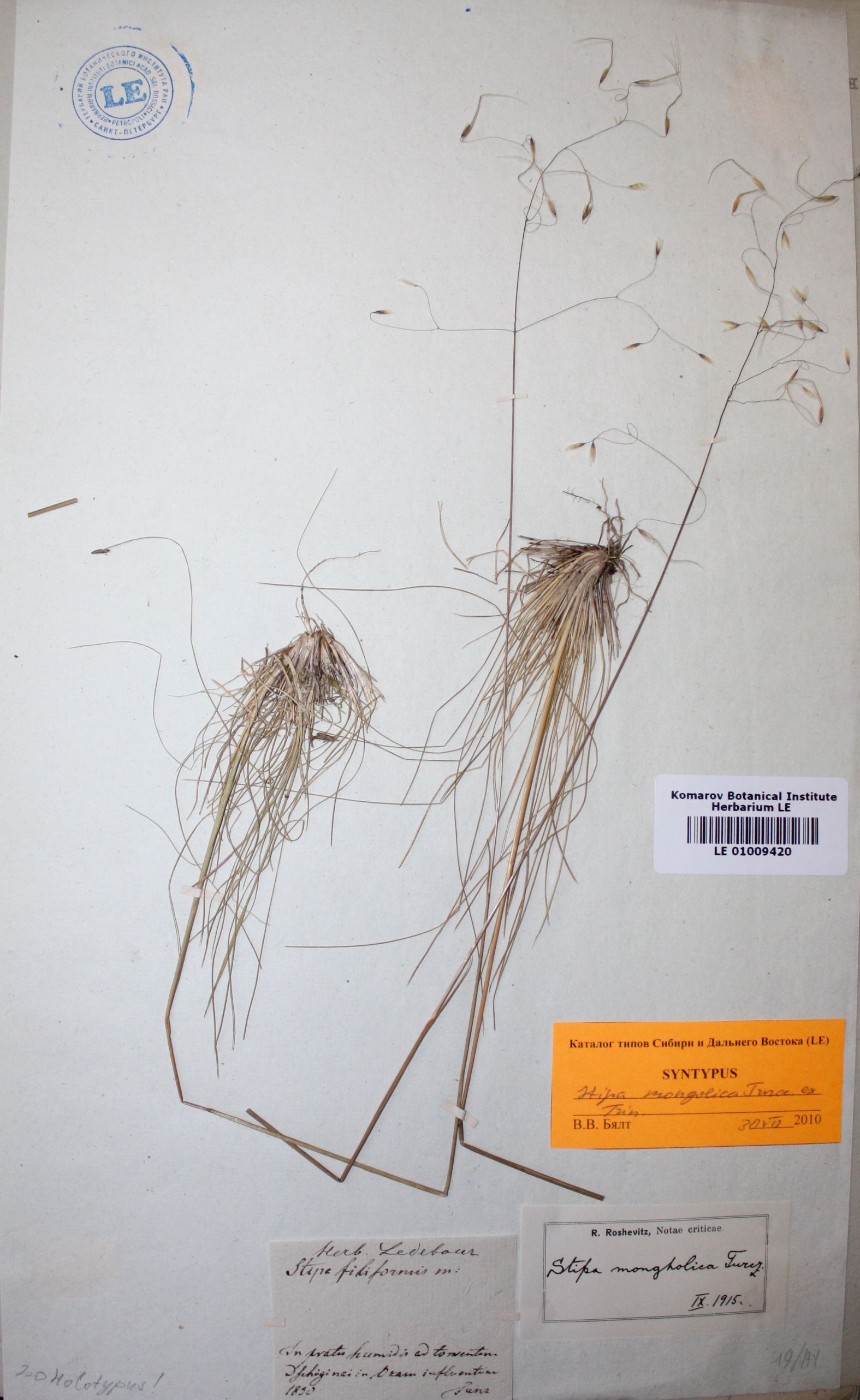


Figure 11. The lectotype of *Ptilagrostis mongholica* subsp. *mongholica* (LE).


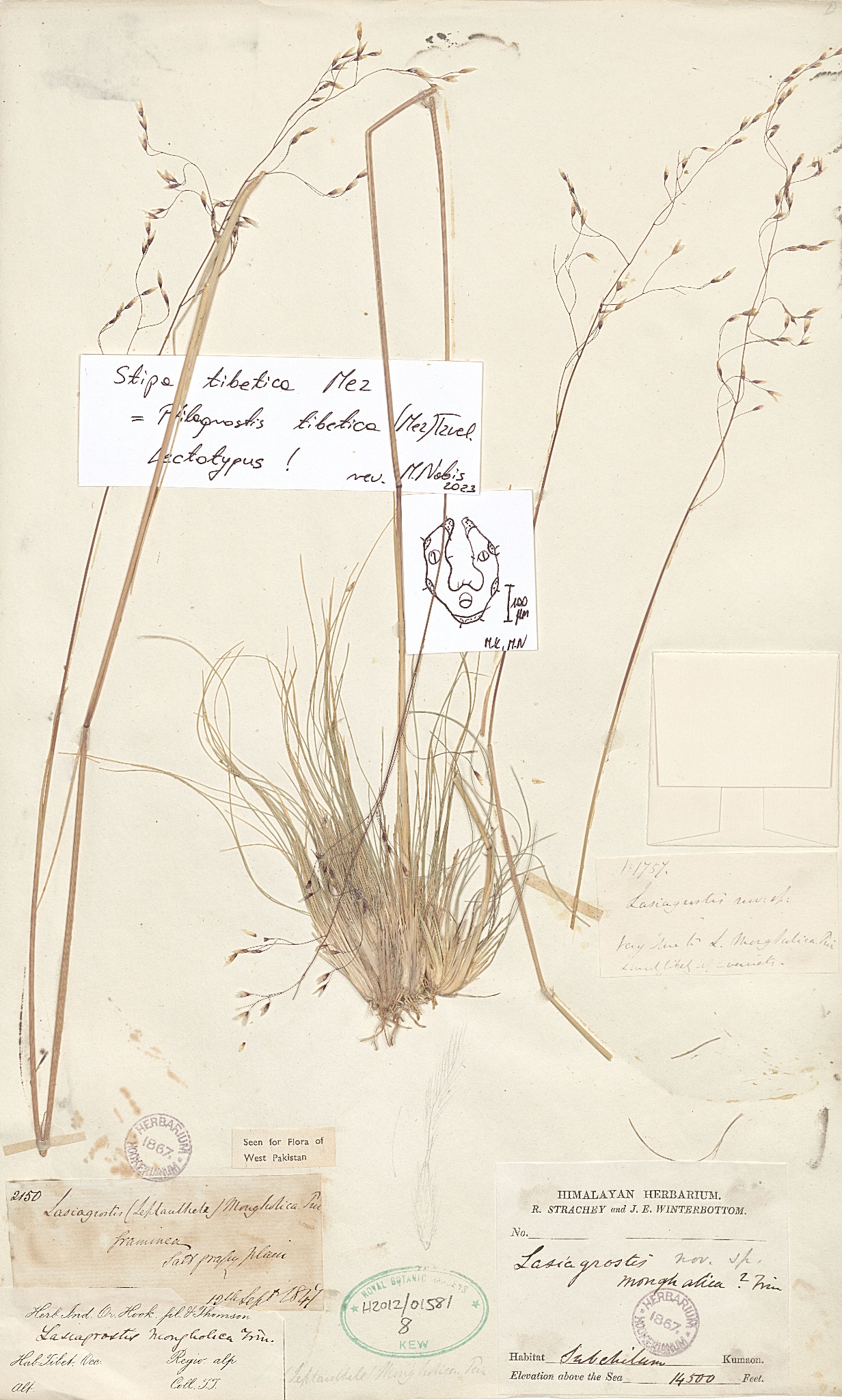


Figure 12. The lectotype of *Ptilagrostis tibetica* (K).


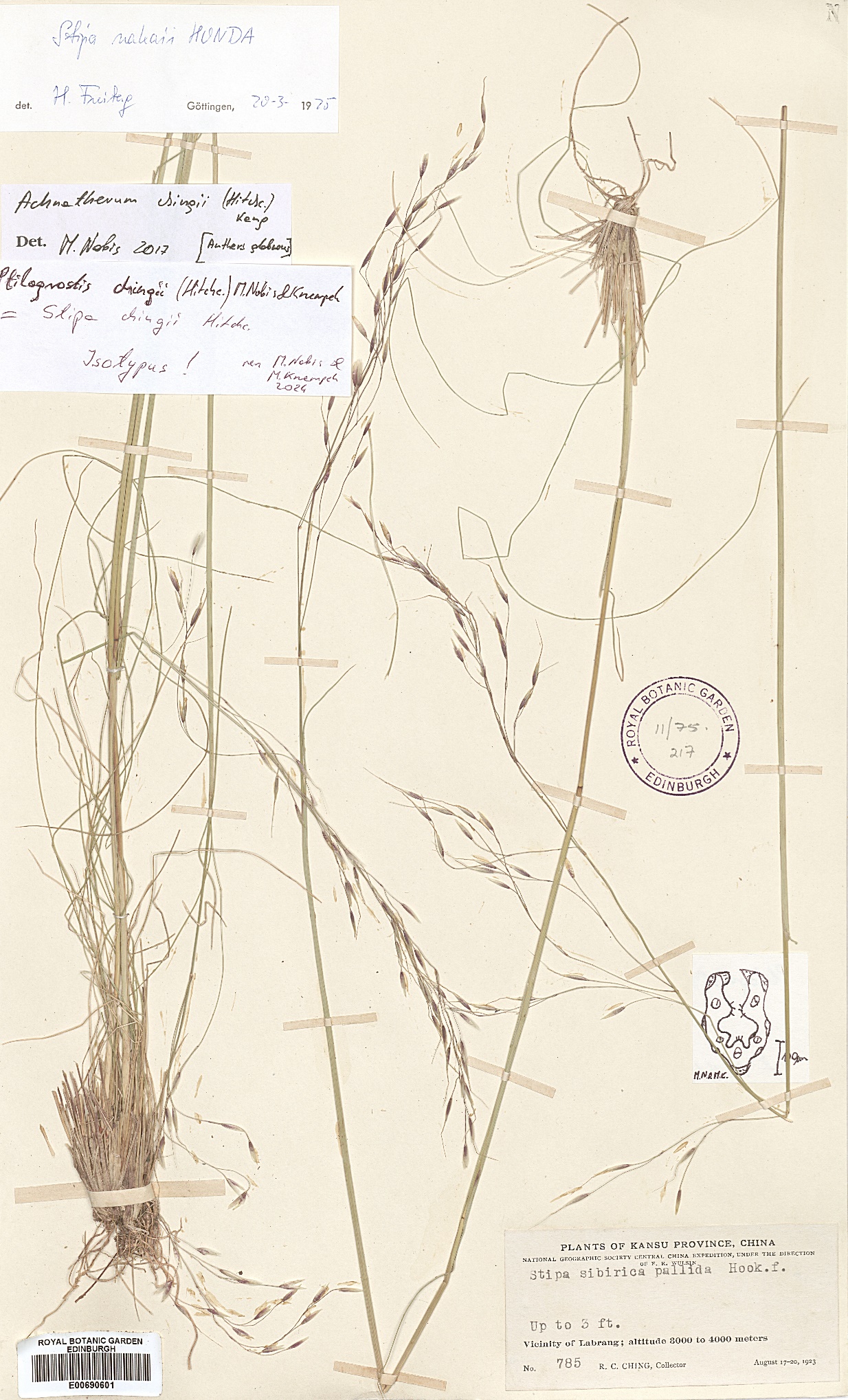


Figure 13. The isotype of *Ptilagrostis chingii* (E).


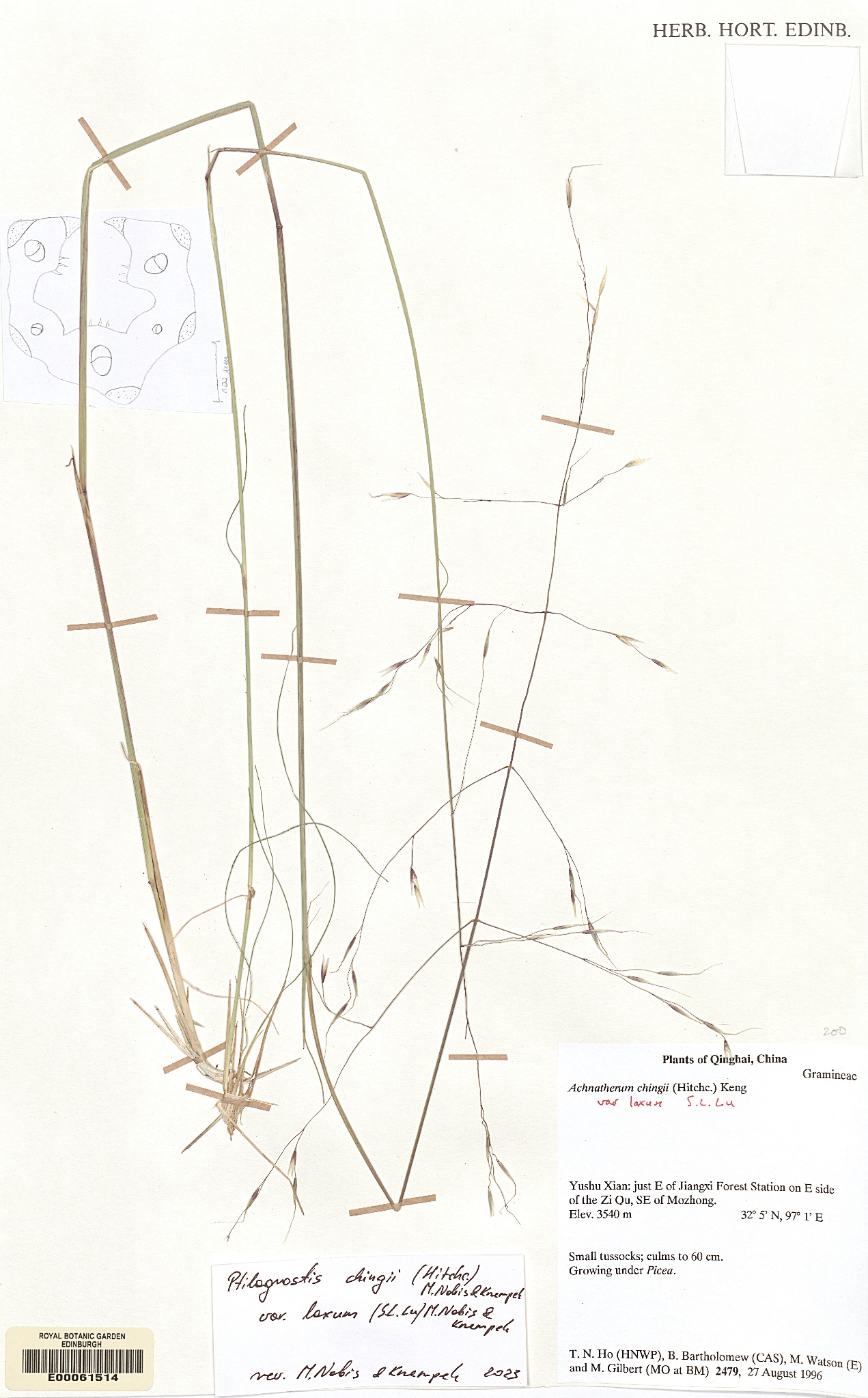


Figure 14. *Ptilagrostis chingii* var. *laxum*, general habitat (E).
